# Stoichiogenomics reveal oxygen usage bias, key proteins and pathways associated with stomach cancer

**DOI:** 10.1038/s41598-019-47533-6

**Published:** 2019-08-05

**Authors:** Xiaoyan Zuo, Bo Li, Chengxu Zhu, Zheng-Wen Yan, Miao Li, Xinyi Wang, Yu-Juan Zhang

**Affiliations:** College of Life Sciences, Chongqing Normal University, Shapingba, Chongqing, 401331 P.R. China

**Keywords:** Molecular evolution, Protein sequence analyses, Cancer microenvironment

## Abstract

Stomach cancer involves hypoxia-specific microenvironments. Stoichiogenomics explores environmental resource limitation on biological macromolecules in terms of element usages. However, the patterns of oxygen usage by proteins and the ways that proteins adapt to a cancer hypoxia microenvironment are still unknown. Here we compared the oxygen and carbon contents ([C]) between proteomes of stomach cancer (hypoxia) and two stomach glandular cells (normal). Key proteins, genome locations, pathways, and functional dissection associated with stomach cancer were also studied. An association of oxygen content ([O]) and protein expression level was revealed in stomach cancer and stomach glandular cells. For differentially expressed proteins (DEPs), oxygen contents in the up regulated proteins were3.2%higherthan that in the down regulated proteins in stomach cancer. A total of 1,062 DEPs were identified; interestingly none of these proteins were coded on Y chromosome. The up regulated proteins were significantly enriched in pathways including regulation of actin cytoskeleton, cardiac muscle contraction, pathway of progesterone-mediated oocyte maturation, etc. Functional dissection of the up regulated proteins with high oxygen contents showed that most of them were cytoskeleton, cytoskeleton associated proteins, cyclins and signaling proteins in cell cycle progression. Element signature of resource limitation could not be detected in stomach cancer for oxygen, just as what happened in plants and microbes. Unsaved use of oxygen by the highly expressed proteins was adapted to the rapid growth and fast division of the stomach cancer cells. In addition, oxygen usage bias, key proteins and pathways identified in this paper laid a foundation for application of stoichiogenomics in precision medicine.

## Introduction

As one of the most common gastrointestinal malignancies in the world, stomach cancer is still a leading cause of death in less developed countries^1^. There are about 400,000 stomach cancer cases a year in China. Statistics showed that the stomach cancer death rate was as high as seventy-two percent in China in 2013^1^. The stomach cancer not only tortures the patients, but also devastates their life with huge economic burdens^2^. Early clinical characteristics of stomach cancer patients are commonly in appetence, emesis and lose weight^3^. Often, none of these symptoms get patients’ attention, and most patients miss the optimal treatment period because they neglect these early warnings. At present, stomach cancer is mainly treated with traditional methods such as surgery and radiotherapy. Because every patient is in different physical conditions, the therapeutic methods cannot meet everyone’s needs. So the survival rate of stomach cancer after treatment is not high. A study of 232 patients in China showed that the post-treatment survival rates after 1, 3 and 5 years were 98.90%, 80.20% and 43.30%, respectively.

Common causes of stomach cancer are (i) unhealthy dietary patterns, smoking, drinking and other unhealthy habits, (ii) pylori infection^4^, and (iii) presence of mold or toxin (such as aflatoxin^5^) –a pathogenic factor of stomach cancer. Metastasis is a complex process that includes multiple steps. After experience, transformation, growth and angiogenesis, new blood vessels are formed to provide nutrition, facilitate subsequent invasion, maintain cancer cells and disseminate them in the circulation system. Stomach cancer cells eventually adhere to and colonized on the secondary organ or tissue^6^, such as lung, bone^7,8^, brain^9,10^ and ovary^11,12^.

Hypoxia is a prominent feature in cancer microenvironments and a major factor affecting the development of cancer proliferation^13^. Stomach cancer cells usually live in a hypoxic microenviroment^14,15^. The occurrence of cancer hypoxia is closely related to cancer cell proliferation, cancer deterioration and resistance therapy^16^. Current research of cancer hypoxia focuses on its effects on morphology^17^, adhesion, mobility, and proangiogenic capacity of cancer cell^18^. In addition, the effect of hypoxia on genes and proteins’ expressions is intensively investigated^19,20^. For example, hypoxia regulates expression of VEGF genes, which further influences the construction of new blood vessels^21^. Hypoxia also induces the expression of transcription factors, the most extensively researched Hypoxia-Inducible Factors (HIFs) including HIF-1^22^, HIF-1α^23,24^, and HIF-2^25^.

Stoichiogenomics aims at investigating element composition and content in biological macromolecules based on genome, transcriptome, proteome, and other omics data^26^. With the development of high-throughput sequencing and assembly technologies^27,28^, more and more sequencing data have been accumulated in the past years. Stoichiogenomics provides a new method for biology sequence analyses, especially on how environmental constraints affect element contents and expression levels of biological macromolecules^29^. In different resource-constrained environments, different organisms and different biological macromolecules experience different natural selections^30,31^. The selective pressures of limited elemental resources are potentially detectable if the limitation persists over an extended period of time^32^. Studies on *Saccharomyces cerevisiae* and *Escherichia coli* have shown that enzymes involved in processing certain chemical elements (carbon, nitrogen, sulfur) contain increased contents of the corresponding elements^33^. In plants, studies have also shown that proteins have increased nitrogen contents in environments where nitrogen is abundant^34^.

As the most important executor of life activities, proteins are directly subjected to natural selective pressures. Stomach cancer is a long-term process, from early stage to terminal stage it takes years to decades^35^. Whether a hypoxic microenvironment lessens the oxygen contents in the expressed proteins in stomach cancer is still unknown. It is interesting to explore the oxygen usage patterns of proteins in stomach cancer cells under hypoxic microenvironments and normal stomach cells. Immunohistochemical method is the most accurate protein quantification method, but it is not widely used for large sets of proteins due to its high costs. At present, huge proteomic data based on immunohistochemical method offered by Human Protein Atlas project (HPA)^36^ are available, which provides us an opportunity to study the relationship between element content and protein expression level in hypoxia stomach cancer, and also offers us a possibility to screen key proteins associated with stomach cancer from the proteome perspective.

This study aims to analyze proteins’ oxygen contents in stomach cancer and normal stomach cells, to test whether an association exists between a protein’s oxygen content and its expression level, and whether a hypoxia environment lessens proteins’ oxygen usageas predicted by the resource limitation theory. In addition, key proteins, genome localization and KEGG enrichment are carried out successively to identify key proteins and determine the closely related pathways, which can potentially facilitate the discovery of biomarkers and novel drug targets for molecular therapeutics of stomach cancer.

## Material and Method

### Data resource

Human genome sequences and annotation (version GRCh38.p7) were downloaded from NCBI (ftp://ftp.ncbi.nih.gov/genomes/H_sapiens/). The proteomes of stomach cancer from 12 individual patients (Table S1,with 13,083 proteins expressed) and two stomach glandular proteomes (with 12,851 proteins expressed in stomach 1-glandular cells and 12,866 proteins expressed in stomach 2-glandular cells) from normal people were retrieved from pathology atlas of Human Protein Atlas project (http://www.proteinatlas.org/). All expression data were obtained using an approach based on immunohistochemical technology. Protein expression evaluation includes the assessment of staining intensity (negative, weak, medium or strong) and staining cells (<25%, 25–75% or >75%) as suggested by HPA. The expression scores of different proteins in different samples were calculated using the according Perl script (Table S2).

### Identification of highly expressed, lowly expressed and DEPs

Initially, we calculated the average expression score of proteins expressed in 12 stomach cancer samples and sorted the proteins by the expression score. The top 3%, top 5% and proteins with a threshold expression score of >=12 were selected as highly expressed proteins, while the bottom 3%, bottom 5% and proteins with a threshold expression score of <=0.1 were referred toas lowly expressed proteins. Then, two normal stomach glandular cells were processed in the same way. Proteins with expression scores marked “−” were deleted and the remaining proteins were sorted. The top 3%, top 5% and proteins with a threshold expression score of >=12 were selected as highly expressed proteins, while the bottom 3%, bottom 5% and proteins with a threshold expression score of <=0.1 as lowly expressed proteins.

Statistical software R (version 3.4.1)^37^ and “limma”^38^ package were used to screen differential expressed proteins. If Log2 ratio >1, proteins were considered as up-regulated proteins. If Log2 ratio <1, proteins were considered as down-regulated proteins. A criterion of *P* < 0.05 was used to determine whether it was statistically significant and a maximum FDR level (q-value) 0.01 was used to reject false positives.

$$\mathrm{Log}2\,{\rm{ratio}}=\,\mathrm{Log}2(\frac{{\rm{Expression}}\,{\rm{score}}\,{\rm{of}}\,{\rm{proteins}}\,{\rm{in}}\,{\rm{stomach}}\,{\rm{cancer}}}{{\rm{Expression}}\,{\rm{score}}\,{\rm{of}}\,{\rm{proteins}}\,{\rm{in}}\,{\rm{normal}}\,{\rm{stomach}}\,{\rm{cells}}})$$

### Calculation method of stoichiogenomics

Element contents in proteins were calculated using algorithms published in the literature^26,39^. The elements content in a protein was estimated as follows:

$$[\ast {\rm{content}}]={\rm{\Sigma }}\,{\rm{wi}}\times {\rm{pi}}/{\rm{L}}{\rm{.}}$$

The * represents one kind of elements, including oxygen, nitrogen, carbon, hydrogen, and sulfur. The wi is the number of * element (or functional group) on the AA side chain (ranging from 0 to 10), pi is the content of the i-th AA, and L is the sequence length. In this paper we considered only oxygen and carbon. The content of an element in multiple sequences was the average value of the element content on the amino acid side chain of all sequences. All calculations were proceeded using Perl scripts developed our lab.

### Functional enrichment

KEGG (Kyoto Encyclopedia of Genes and Genomes) database^40,41^ was widely used to elucidate the gene and pathways’ functions. KEGG annotations and enrichment for human genes were extracted and performed by Gene Set Enrichment Analysis (GSEA) (http://software.broadinstitute.org/gsea/index.jsp)^42^.

### Statistics and visualizations

Statistical software R (version 3.4.1)^37^ was used for statistical analysis. Packages “ggplot2”^43^, “ggrepel”^44^, and “grid”^45^ were used for data visualization. R package “circlize”^46^ were used to locate genes on genome. R package “Clusterprofiler”^47^ was used to show functional enrichment results.

### Human participants and animal rights

This article does not contain any studies with human participants or animals performed by any of the authors.

## Results and Discussion

### Oxygen contents of all proteins expressed in stomach cancer and normal stomach glandular cells

**Figure 1:**
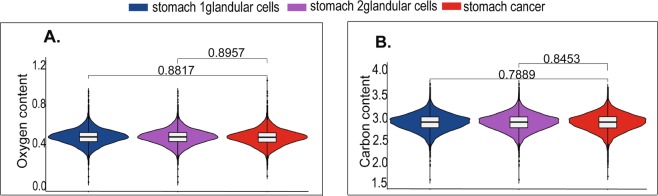
Distribution of oxygen and carbon contents of all expressed proteins in stomach cancer and 2 stomach glandular cells. (**A**) Oxygen contents of all expressed proteins. (**B**) Carbon contents of all expressed proteins. Statistic results of Mann-Whitney U test were shown.

The oxygen contents of all proteins from stomach cancer and stomach normal tissues were calculated and their comparisons were performed. The average oxygen content of all expressed proteins was 0.479 in both stomach cancer cells and two stomach glandular cells (Fig. 1A, Table S3). Also, the average carbon contents in all expressed proteins were almost the same in these 3 groups. No significant differences in oxygen and carbon contents were detected (Fig. 1A, Table S3). Although no difference was noticed in this step, we only analyzed oxygen contents in all proteins. We did not associate it with the protein’s expression level.

### Oxygen content of highly and lowly expressed proteins in stomach cancer and normal stomach glandular cells

To better explore the association between a protein’s oxygen content and its expression level, we further calculated and compared the oxygen contents in the highly and lowly expressed proteins in stomach cancer and 2 kinds of normal stomach glandular cells. In order to ensure the reliability of the results, the highly and lowly expressed proteins were screened out based on the top/bottom 3% expressed proteins, the top/bottom 5% expressed proteins and proteins with threshold expression score of ≥12/≤0.1. Meanwhile the carbon content in each protein was calculated and used as a control for oxygen content comparison. As the principal element of organisms, carbon is a suitable control for element composition analysis^33^.

**Figure 2:**
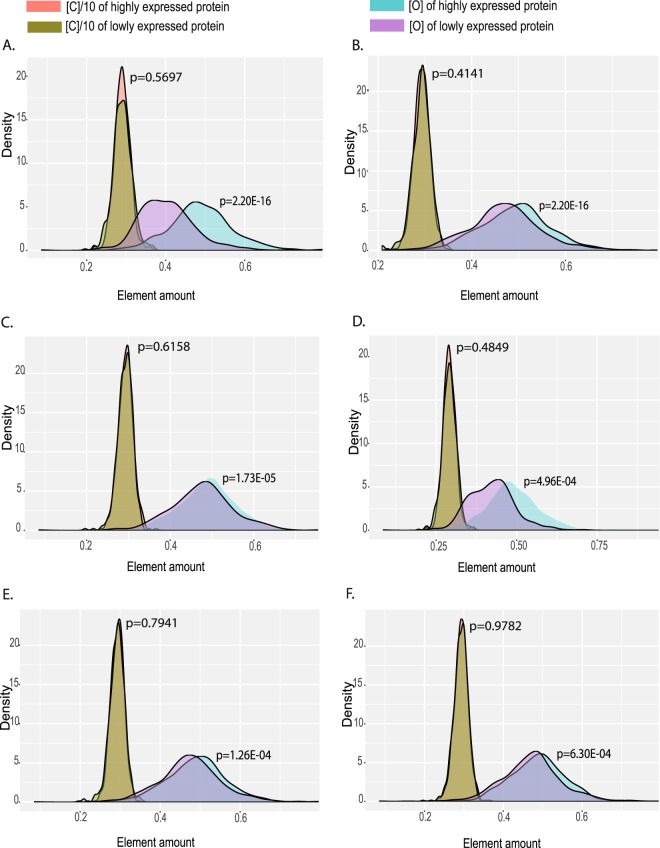
Distribution of oxygen and carbon contents of highly expressed and lowly expressed proteins in stomach cancer and 2 stomach glandular samples. (**A**) Comparison of top 3% highly and lowly expressed proteins in stomach cancer. (**B**) Comparison of top 5% highly and lowly expressed proteins in stomach cancer. (**C**) Comparison of top 3% highly and lowly expressed proteins in stomach 1-glandular cells. (**D**) Comparison of top 5% highly and lowly expressed proteins in stomach 1-glandular cells. (**E**) Comparison of top 3% highly and lowly expressed proteins in stomach 2-glandular cells. (**F**) Comparison of top 5% highly and lowly expressed proteins in stomach 2-glandular cells.

Our results showed that, in stomach cancer, the oxygen contents in the highly expressed proteins were significantly different from that in and the lowly expressed proteins. Average oxygen content in the highly expressed proteins was higher than that in the lowly expressed proteins (Fig. 2A,B), but no significant differences in carbon contents were observed (Fig. 2, Table S4). In addition, in two kinds of normal stomach glandular cells, the average oxygen contents in the highly expressed proteins were also higher than that in the lowly expressed proteins. Similarly, there were no significant carbon content differences between the highly and lowly expressed proteins. All results were agreed for proteins expressed at the top/bottom 3% or 5%, or proteins with a preset threshold score (Figs 2, S1–3).

Furthermore, we found that the oxygen content differences between the highly and the lowly expressed proteins were more pronounced in stomach cancer than that in normal stomach glandular cells (Fig. 2). Similar results were obtained from comparison between the top/bottom3%, the top/bottom5% and proteins with a preset threshold score of ≥12/≤0.1 for the highly/lowly expressed proteins (Figs S1–3). All together, these results suggested that there was an association between proteins’ oxygen contents and their expression levels in stomach cancer and stomach glandular cells, and this association was more pronounced in stomach cancer cells.

### The association between oxygen content and expression ratio of DEPs

**Figure 3:**
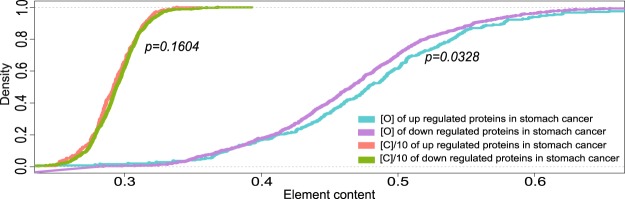
Distribution of oxygen and carbon contents of DEPs between stomach cancer and 2 stomach glandular samples. In order to present results within the same scale, the values of carbon content were reduced by ten times.

After detecting the phenomenon that oxygen content in the highly expressed proteins was higher than that in the lowly expressed proteins in stomach cancer and stomach glandular cells, we checked whether this phenomenon existed between the up and the down regulated proteins. By DEPs screening, 246 up regulated proteins and 816 down regulated proteins were identified in stomach cancer cells (Table S5). Then we calculated and compared the oxygen contents in these DEPs. The average oxygen content in the up regulated proteins was 0.481 and in the down regulated proteins was 0.466 in stomach cancer cells. Statistical analysis showed that the oxygen content in the up regulated proteins was 3.2% higher than that in the lowly expressed proteins (*p* = 3.28E-2, Wilcoxon test, Table S5). The distributions of oxygen in the up and the down regulated proteins were largely separated (Fig. 3).

Meanwhile the carbon content was calculated and used as a control for oxygen content comparison. The average carbon contents in the up regulated proteins and down regulated proteins were not significantly different (*p* = 0.1604, wilcoxon test, Table S5). As it was shown, the distribution of carbon in the up and down regulated proteins virtually overlapped, which indicated no significant differences for carbon contents in the DEPs in stomach normal tissues and stomach cancer tissues (Fig. 3).

### Localization of genes encoding DEPs on genome

**Figure 4:**
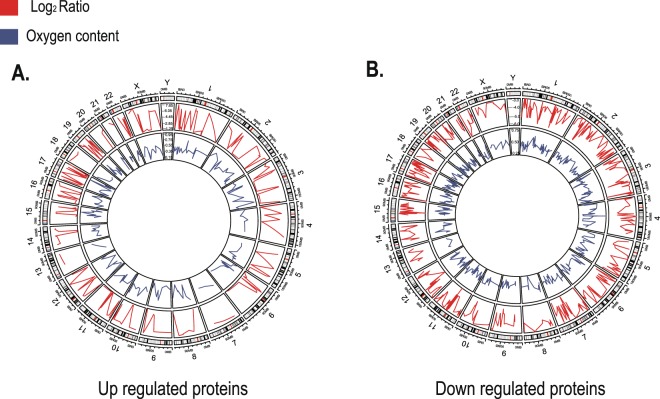
Genome localization of genes encoding DEPs and expression ratio, oxygen content of DEPs. (**A**) Genes encoding up regulated proteins. (**B**) Genes encoding down regulated proteins. The outer circle is the karyogram of human. Red curve reflects the expression ratio (log2ratio) of DEPs in middle circle. Blue curve reflects the oxygen content of DEPs in the inner circle.

After identification of the DEPs, we further determined the genome location of genes encoding the up and down regulated proteins. Two hundred forty four up regulated genes and 582 down regulated genes were located on human genome (Fig. 4). Most differentially expressed genes (DEGs) were distributed on chromosome 1, with 28 up regulated and 64 down regulated genes respectively. Fewest differentially expressed genes were distributed on chromosome 13, with only 2 genes being located. For the up regulated genes, a few were distributed on chromosome 7 and chromosome 20, with 3 being located. And their distribution on chromosome 13 was the least. In addition, for the down regulated genes, a few were distributed on chromosome 13 and 18, with 9 genes being located. And their distribution on chromosome 21 was the least, with only 7 genes being located. It is interesting to note that all differentially expressed genes were absent from Y chromosome.

### Functional dissection of key proteins in stomach cancer

Up regulated proteins commonly possess higher oxygen contents than down regulated proteins. Among the up regulated proteins (246, Table S6), ANP32A, ANP32B and ANP32E are acidic leucine-rich nuclear phosphoproteins. ANP32A is a novel molecular target of sphingoid bases that regulates cellular proliferation, differentiation, caspase-dependent and caspase-independent apoptosis, and plays a role in acetyltransferase inhibitory activity, chromatin remodeling and transcriptional regulation^48^. It is evidenced that ANP32A expressed at a much higher level in advanced cancers than in early-stage cancers^49^. ANP32B protein is a histone chaperone and oncogene playing essential roles in breast carcinogenesis^50^. TPM3 is tropomyosin, which has been proven as a regulator of cancer cell transformation^51^. As a component of a spliceosome, NKTR has functions in protein folding and is reported to play a key role in lung cancer progression^52^. HSP90AB1 encodes a member of the heat shock protein 90 family involved in signal transduction, protein folding and degradation and morphological evolution, which is thought to play roles in gastric apoptosis and inflammation^53^. HDGF is closely related to the occurrence and metastasis of cancer^54^, and could be used as a target for mir-141 to inhibit the proliferation of stomach cancer cells^55^.

Among the down regulated proteins (816, Table S6), we paid special attention to proteins with low oxygen contents. MAL2 and PTGDR2 possess low oxygen contents (<0.32, Table S6). Studies have shown that mal2 gene encoding a four-transmembrane protein of the MAL family, is amplified and over expressed in breast and papillary thyroid carcinoma^56,57^. However, it is interest to see the down regulation of MAL2 on the protein level in stomach cancer. PTGDR2 (Prostaglandin D2 receptor 2) is involved in signal transduction and has the ability to restrict the self‐renewal of GC cells *in vitro* and suppress tumor growth and metastasis *in vivo*^58^. The down regulation of these proteins is closely related to the promotion of occurrence and metastasis of stomach tumor.

**Figure 5:**
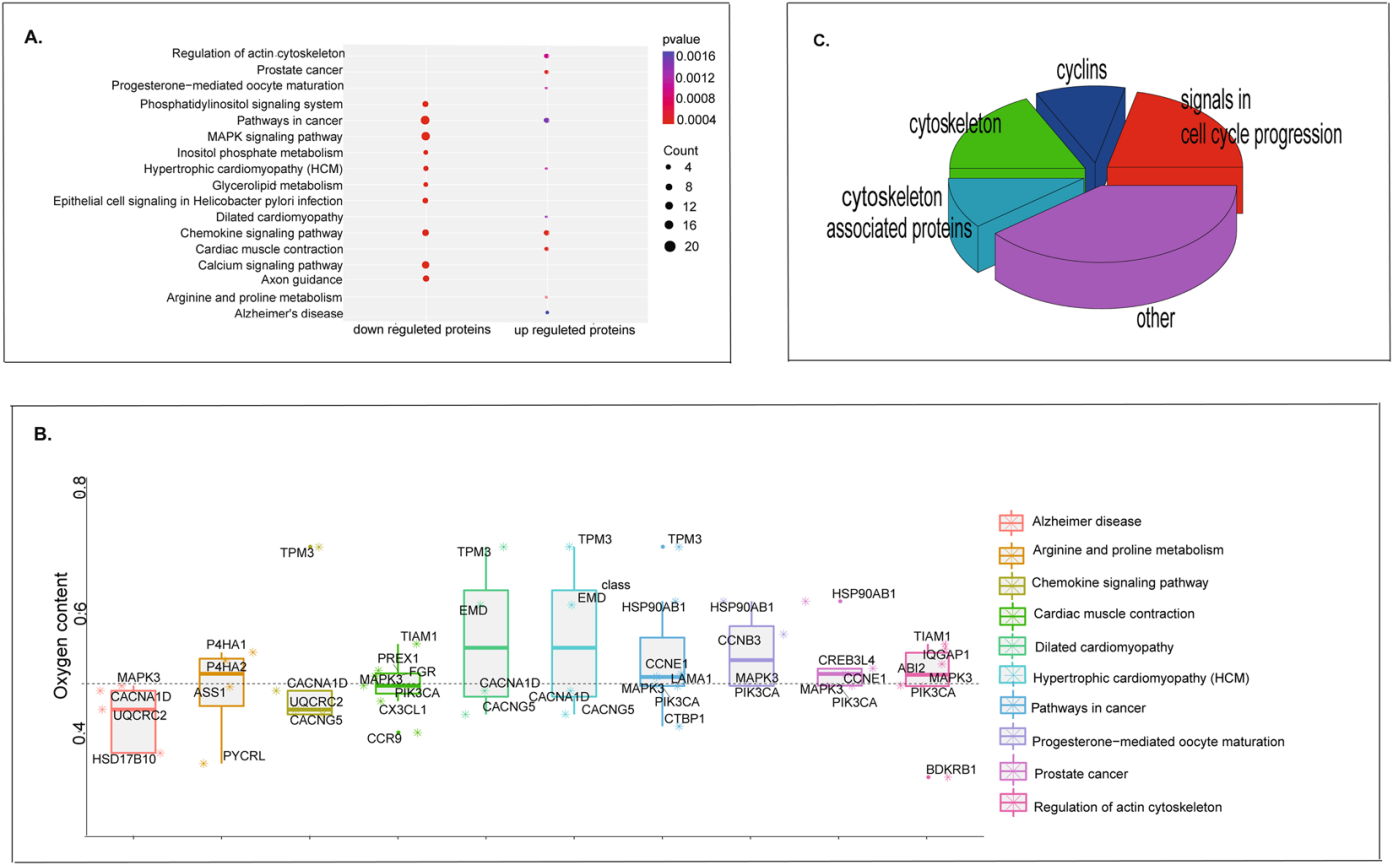
Functional dissections of DEPs. (**A**) KEGG enrichment of up regulated proteins and down regulated proteins. (**B**) Oxygen content of up regulated proteins in ten pathways enriched by up regulated proteins. (**C**) Functional dissection of proteins, with oxygen content equal or higher than 0.478 in up regulated proteins enriched pathways.

The gene-annotation enrichment analysis is a promising systematic strategy to efficiently examine large gene lists in a network context, and assemble a summary of the most enriched and pertinent biology. Therefore, KEGG enrichments were performed for both genes encoding the up-regulated proteins and the down-regulated proteins in our study. Genes of the up regulated proteins in stomach cancer were enriched in ten pathways (Fig. 5A, Table S7), including pathways in cancer, chemokine signaling pathway, regulation of actin cytoskeleton, cardiac muscle contraction, prostate cancer, Alzheimer’s disease, progesterone-mediated oocyte maturation, arginine and proline metabolism, Hypertrophic cardiomyopathy (HCM), and Dilated cardiomyopathy(Fig. 5A). Additionally, genes of the down regulated proteins were also enriched in ten pathways (Fig. 5A, Table S7). Twenty genes were enriched in pathways in cancer, and nineteen genes enriched in MAPK signaling pathway, calcium signaling pathway (15 genes) and chemokine signaling pathway (12 genes). Other genes were enriched in axon guidance (11 genes), phosphatidylinositol signaling system (10genes), hypertrophic cardiomyopathy (HCM) (8 genes), epithelial cell signaling in Helicobacter pylori infection (9 genes), inositol phosphate metabolism (7 genes), glycerolipid metabolism (7 genes). Among them, five pathways were signaling pathways. The enriched pathways identified here provided bases for studying the molecular mechanisms of stomach cancer.

Further wedissected the up-regulated protein enriched pathways in details to understand which kind of up-regulated proteins consumed more oxygen in the hypoxic microenvironment of stomach cancer. The functions of those proteins with oxygen contents higher than 0.478 (the average of three proteomes) were checked one by one manually (Fig. 5B. Table S8). We found that, except some oncoproteins, most of these proteins with high oxygen contents were involved in cell division, including cytoskeleton, cytoskeleton associated proteins, cyclins and signaling proteins in cell cycle progression (Fig. 5C).

## Discussion

In this study, we identified 246 up regulated and 816 down regulated proteins in stomach cancer proteomes. These marker proteins could lead to new ways for stomach cancer diagnosis and treatment. The genome location of key genes laid foundation for genome study of stomach cancer, and allowed studies of the upstream and the downstream regulations of key genes, which could ultimately improve the understanding of the molecular mechanism of stomach cancer.

Through examining the pathway enriched by the up-regulated and the down-regulated proteins, we found that the up regulated proteins were mainly enriched in tumorigenesis, metastasis and cell cycle disorders. The down-regulated proteins were more related to signaling pathways. Interestingly, the chemokine signaling pathway occurred simultaneously in the up-regulated and the down-regulated protein enrichments. In fact, the positive or negative effects of chemokines on tumorigenesis and development are multifaceted and uncertain. Some chemokines inhibit tumor growth and development by activating immunocompetent cytotoxic cells^59^ or inhibiting tumor-related angiogenesis^60^. Other chemokines promote tumor development by promoting tumor cell proliferation and migration, promoting the secretion of proteolytic enzymes and inducing angiogenesis^61^.

The up regulated proteins were mostly enriched in pathways of cancer, degradation of biomolecules (regulation of actin cytoskeleton) and so on. Firstly, 7 proteins were enriched in the pathway of regulation of actin cytoskeleton. As far as we know, alterations in cytoskeletal structure is one of characteristics of malignant transformed cells^62^. Moreover, one of the key steps in tumor metastasis is the migration and invasion of tumor cells. In this process, cell adhesion changes. The cytoskeleton system is directly related to cell adhesion, and abnormal cytoskeleton may be the structural basis of abnormal cell adhesion^63^. This process requires the rearrangement of cytoskeleton^64^, which could explain why this pathway was enriched by the up-regulated proteins. Secondly, Control of cell division cycle is closely related to stomach cancer. Eighty six up regulated proteins were enriched in progesterone-mediated oocyte maturation, which is an important process in cell cycle regulation, as its dysregulation can lead to uncontrolled proliferation of cancer cells^65^. In addition, studies have shown that progesterone-mediated oocyte maturation is related to the pathogenesis of glioma^66^.

The down regulated proteins were mostly enriched in the signal pathways, which play important roles in cell proliferation, division, migration, senescence and apoptosis. Among the five enriched signal pathways, MAPK signaling pathway is the most enriched. In cancer, constitutive activating MAPK signaling pathway often leads to promotion of abnormal cell growth and tumorigenesis. However, suppressing the MAPK signaling pathway has shown to be important in the development and progression of cancer cells^67,68^, which is coinciding with our results. Comparison between pathways enriched by the up and down regulated proteins provide a base to reveal molecular mechanisms of stomach cancer in its occurrence and progression.

An association of oxygen content and protein expression level was detected in both stomach cancer and stomach glandular cells, while this association was more pronounced in stomach cancer cells. However, no carbon content differences between the highly and the lowly expressed proteins were detected. Besides, we found that the oxygen contents in the up regulated proteins were higher than that in the down regulated proteins in stomach cancer proteome, while no differences of carbon contents were observed. For the DEPs, oxygen contents of the up regulated were 3.2% higher than that in the down regulated proteins in stomach cancer cells.

Our results suggested that cancer indeed affected the oxygen contents in macromolecules. However, stomach cancer hypoxia microenvironment didn’t lessen the oxygen contents in the up regulated proteins as predicted by the resource limitation theory^32^. According to the resource limitation theory, the environmental supplies of elements limit organisms’ growth and reproduction. Under conditions of resource limitation for a given element, selection would happen to reduce the use of these limiting elements and facilitate construction of the cellular components that are most needed. Consistent with resource limitation theory, that protein N content declines with gene expression intensity has been confirmed in plants. Work with microorganism has also evidenced the association of element investment with expression level^69^. However, the negative effects of hypoxic microenvironment on oxygen content in the up-regulated proteins were not detected in human stomach cancer cells. On the contrary, we found that the up regulated proteins possessed more oxygen than that in the down regulated proteins, indicating that proteins did not save the use of oxygen element in a hypoxia microenvironment.

Pathways enrichment of the up regulated proteins was used to understand why the up regulated proteins in cancer cells consumed more oxygen element in the hypoxic microenvironment. Among 10 pathways enriched by the up regulated proteins, 3 pathways regulated actin cytoskeleton, cardiac muscle contraction and pathway of progesterone-mediated oocyte maturation, closely associated with the activities of cytoskeletons. Moreover, we found that, except some oncogenes, the up-regulated proteins with high oxygen contents were involved in cell division, including cytoskeleton, cytoskeleton associated proteins, cyclins and signaling proteins in cell cycle progression. Studies have shown that the cytoskeleton proteins are rich of acid amino acids and oxygen^70^.

Resource limitation theory might not be applicable to human cancer cells. However, from the perspective of functional assignment, more oxygen consumed by the up-regulated proteins could be explained by the need for rapid growth and faster division of cancer cells. In these processes, cytoskeleton proteins play a major role. Cell division requires frequent participation of cytoskeletal proteins. In addition, cancer and normal cells are both constantly dividing, and cancer cells are dividing uncontrolled and faster than normal cells. It could be explained now why the up regulated proteins (e.g. TPM3) possessed more oxygen than the down regulated proteins in stomach cancer cells, since functional dissection of the up regulated proteins suggested that these proteins were mainly involved in cytoskeleton and cell division. We expect that the faster the cells divide, the higher the oxygen content in the highly expressed or the up regulated proteins we can observe.

Large amount of oxygen consumption by the up-regulated proteins might be one cause in the formation of a hypoxic microenvironment and further worsen the hypoxic situation. It is accepted that rapid growth of cancer cells leads to vascular disorders, decreased blood supply of cancer cells, and increased consumption of oxygen. This imbalance between supply and consumption of oxygen leads to a hypoxic microenvironment in cancer. In terms of stoichiogenomics, stomach cancer has shaped DEPs’ oxygen usage biases. Deviated from the resource limitation theory^32^, the highly expressed and up regulated proteins did not preserve the use of oxygen in a hypoxic microenvironment of stomach cancer. We speculate that the increased amount of oxygen consumed by the up-regulated proteins is not caused by a hypoxic microenvironment. On the contrary, the oxygen consumption promotes the formation of a hypoxic microenvironment in stomach cancer.

## Conclusion

Based on the comprehensive immunohistochemical data, a total of 1,062 DEPs associated with stomach cancer were screened out, which distributed on all chromosomes except Y. An association of oxygen content and protein expression level was revealed in stomach cancer and stomach glandular cells, which is more pronounced in stomach cancer. Besides, oxygen contents in the up regulated proteins were higher than that in the down regulated proteins in stomach cancer cells. Pathway enrichment results of the up regulated proteins include regulation of actin cytoskeleton, cardiac muscle contraction and pathway of progesterone-mediated oocyte maturation. Moreover, functional dissection of the up-regulated proteins with high oxygen contents showed that most of them are cytoskeleton, cytoskeleton associated proteins, cyclins and signals in cell cycle progression. Our results suggested that the increased amount of oxygen consumed by the up-regulated proteins was driven by the functional need to adapt to rapid growth and fast division of cancer cells. More oxygen used by proteome might be a cause in the formation of a hypoxic microenvironment of stomach cancer and further worsens this situation.

Our findings for the first time revealed that different patterns of oxygen usage could be detected between proteomes of cancer and normal cells, which helps us to better understand protein’s evolutionary adaptation to rapid growth and fast division of stomach cancer in terms of element compositions. It provides new insights in cancer-related biomacromolecules research and lays the foundation for application of stoichiogenomics in precision medicine. In addition, oxygen usage biases, key proteins and pathways associated with stomach cancer initiation and progression can facilitate disease diagnosis and novel drug target discovery for molecular therapeutics of stomach cancer.

## Supplementary information

Supplementary file

## Data Availability

Human stomach cancer and stomach glandular cells proteomes were retrieved from Human Protein Atlas project (http://www.proteinatlas.org/).
