# Supplementary material for: Stoichiogenomics reveal oxygen usage bias, key proteins and pathways associated with stomach cancer
Source: Sci Rep. 2019 Aug 5;9:11344. doi: 10.1038/s41598-019-47533-6 (PMC6683168; doi:10.1038/s41598-019-47533-6)
Supplement: Supplementary file 1 — Supplementary file [file 41598_2019_47533_MOESM1_ESM.docx]

Supplementary files for

**Stoichiogenomics reveal oxygen usage bias, key proteins and pathways associated with stomach cancer**

Xiaoyan Zuo^1^, Bo Li^1^, Chengxu Zhu^1^, Zheng-Wen Yan^1^, Miao Li^1^, Xinyi Wang^1^, Yu-Juan Zhang^1^*

^1^*College of Life Sciences, Chongqing Normal University, Shapingba, Chongqing 401331, PR China*

*Author for Correspondence:

Yu-Juan Zhang, College of Life Sciences, Chongqing Normal University, Shapingba, Chongqing 401331, PR China, telephone number: +86-23-65910315, fax number: +86-23-65910315, e-mail address: zhangyj@cqnu.edu.cn

**This PDF file includes**

Tables. S1 to S8

Figures. S1 to S3.

**Other Supplementary Material for this manuscript includes the following:**

Supplementary files

**Tables**

**Table S1: The specific information of the stomach cancer patients.**

| **Patient id** | **Gender** | **age** | **Sampling position** | **Exclusion of diagnostic results** | **Staining** | **Intensity** | **Quantity** | **Location** |
| --- | --- | --- | --- | --- | --- | --- | --- | --- |
| 2626 | Female | 79 | Stomach (T-63000) | Adenocarcinoma, NOS (M-81403) | Low | Weak | 75%-25% | Cytoplasmic/membranou |
| 2066 | Male | 76 | Stomach (T-63000) | Adenocarcinoma, NOS (M-81403) | Not detected | Negative | Negative | None |
| 3526 | Female | 60 | Stomach (T-63000) | Adenocarcinoma, NOS (M-81403) | Low | Weak | 75%-25% | Cytoplasmic/membranou |
| 2557 | Female | 73 | Stomach (T-63000) | Adenocarcinoma, NOS (M-81403) | Medium | Moderate | >75% | Cytoplasmic/membranous/nuclear |
| 657 | Female | 52 | Stomach, upper (T-62350) | Adenocarcinoma, NOS (M-81403) | Not detected | Weak | <25% | Nuclear |
| 1266 | Male | 47 | Stomach, upper (T-62350) | Adenocarcinoma, NOS (M-81403) | Not detected | Weak | <25% | Nuclear |
| 2473 | Male | 59 | Stomach (T-63000) | Normal tissue, NOS (M-00100) | Not detected | Negative | Negative | None |
| 3063 | Female | 76 | Stomach (T-63000) | Adenocarcinoma, NOS (M-81403) | Medium | Moderate | >75% | Nuclear |
| 148 | Female | 43 | Stomach, lower (T-63700) | Adenocarcinoma, NOS (M-81403) | High | Strong | >75% | Nuclear |
| 2378 | Male | 59 | Stomach (T-63000) | Adenocarcinoma NOS (M-81403) | Medium | Moderat | >75% | Cytoplasmic/membranous/nuclear |
| 2959 | Female | 59 | Stomach (T-63000) | Adenocarcinoma, NOS (M-81403) | Medium | Moderat | >75% | Nuclear |
| 360 | Female | 93 | Stomach, lower T-63700) | Adenocarcinoma, NOS (M-81403) | High | Strong | >75% | Cytoplasmic/membra |

**Table S2: Evaluation of protein expression scores.**

| **Staining intensity** | **Staining cells** | **Degree** | **Expression score** |
| --- | --- | --- | --- |
| Negative | — | Not detected | 0 |
| Weak | <25% | Low | 3*0.625=1.875 |
|  | 25-75% or >75% |  |  |
| Moderate | <25% | Medium | 6*0.625=3.75 |
|  | 25-75% or >75% |  |  |
| Strong | <25% | High | 12*0.625=7.5 |
|  | 25-75% or >75% |  |  |

**Table S3：Oxygen content and carbon content of all proteins expressed in stomach cancer and stomach glandular cells.**

|  |  |  |  | **Comparison of [O]** | | | | | | **Comparison of [C]** | | | | | |  |
| --- | --- | --- | --- | --- | --- | --- | --- | --- | --- | --- | --- | --- | --- | --- | --- | --- |
|  | Number of genes | Mean [O] | Mean [C] | Kolmogorov-Smirnov test | | Mann-Whitney U test | | Wilcoxon test | | Kolmogorov-Smirnov test | | Mann-Whitney U test | | Wilcoxon test | |  |
| Stomach 1-glandular cells | 9495 | 0.479732 | 2.925658 | 1 |  | 0.8817 |  | 0.8817 |  | 0.9956 |  | 0.7889 |  | 0.7889 |  | |
| Stomach cancer | 8967 | 0.479944 | 2.925089 |  | 1 |  | 0.9848 |  | 0.8957 |  | 0.998 |  | 0.9415 |  | 0.8453 | |
| Stomach 2-glandular cells | 9429 | 0.479745 | 2.925830 |  |  |  |  |  |  |  |  |  |  |  |  |  |

**Table S4. Oxygen and carbon content of highly and lowly expressed proteins in stomach cancer and stomach glandular cells.**

| **Scale** | **Sample** | **Number of proteins** | **Number of selected proteins** | **Mean [O] of highly expressed proteins** | **Mean [O] of lowly expressed proteins** | **Comparison of [O] (*P* value) (Kolmogorov-Smirnov test)** | **Comparison of [O] (P value) (Wilcoxon test)** | **Mean [C] of highly expressed proteins** | **Mean [C] of lowly expressed proteins** | **Comparison of [C] (*P* value) (Kolmogorov-Smirnov test)** | **Comparison of [C] (P value) (Wilcoxon test)** |
| --- | --- | --- | --- | --- | --- | --- | --- | --- | --- | --- | --- |
| 3% | stomach cancer | 13083 | 392 | 0.4899 | 0.4028 | 2.2E-16 | 2.20E-16 | 2.901 | 2.8998 | 0.5097 | 0.8687 |
|  | stomach 1-glandular cells | 12851 | 385 | 0.4941 | 0.4715 | 0.0000173 | 0.0000122 | 2.9348 | 2.9231 | 0.6158 | 0.8404 |
|  | stomach 2-glandular cells | 12866 | 385 | 0.4943 | 0.4751 | 0.0001269 | 0.0001953 | 2.9324 | 2.9203 | 0.7941 | 0.6028 |
| 5% | stomach cancer | 13083 | 654 | 0.4897 | 0.4229 | 2.2E-16 | 2.2E-16 | 2.9054 | 2.9073 | 0.4141 | 0.7363 |
|  | stomach 1-glandular cells | 12851 | 642 | 0.4907 | 0.4758 | 0.0004967 | 0.000139 | 2.934 | 2.9189 | 0.4849 | 0.4674 |
|  | stomach 2-glandular cells | 12866 | 643 | 0.4862 | 0.472 | 0.0006305 | 0.0001343 | 2.9261 | 2.9253 | 0.9782 | 0.8947 |
| Threshold | stomach cancer | 13083 | 998 | 0.4887 | 0.4427 | 2.2E-16 | 2.20E-16 | 2.9125 | 2.9181 | 0.08636 | 0.1879 |
|  | stomach 1-glandular cells | 12851 | 2866 | 0.4770 | 0.4742 | 0.02323 | 3.95E-02 | 2.9246 | 2.9151 | 0.04269 (Notes: P value of [C]:[O] =0.3462) | 0.4903 |
|  | stomach 2-glandular cells | 12866 | 2792 | 0.4786 | 0.4753 | 0.01056 | 3.26E-02 | 2.9275 | 2.9171 | 0.2260 | 0.2617 |

**Table S5: Oxygen and carbon contents of differential expressed proteins in stomach cancer.**

|  | **Differential expressed protein** | | |
| --- | --- | --- | --- |
|  | Number of up regulated proteins | Mean [C] | Mean [O] |
|  |  |  |  |
| Stomach | 816 | 2.937294 | 0.4662469 |
| Stomach cancer | 246 | 2.914846 | 0.4807548 |
| P value (Wilcoxon test） | - | 2.20E-16 | 2.20E-16 |
| P value (Kolmogorov-Smirnov test) | - | 1.60E-01 | 3.28E-02 |
|  |  |  |  |

**Table S6: Differential expressed proteins identified between stomach cancer and 2 stomach glandular proteomes.**

| **Gene ID** | **Ensemble ID** | **KO** | **Gene name** | **Chromosome** | **start** | **end** | **logFC** | **logCPM** | **PValue** | **FDR** | **[O]** | **[C]** | **Up or Down** |
| --- | --- | --- | --- | --- | --- | --- | --- | --- | --- | --- | --- | --- | --- |
| 7105 | ENSG00000000003 | K17295 | TSPAN6 | chrX | 100627108 | 100637104 | 1.70095328 | 8.179657537 | 0.000091 | 0.00127008 | 0.408163265 | 3.134693878 | Up |
| 2268 | ENSG00000000938 | K08891 | FGR | chr1 | 27612289 | 27635561 | 5.652753462 | 6.187596075 | 0.000254685 | 0.003259292 | 0.493383743 | 3.015122873 | Up |
| 6376 | ENSG00000006210 | K05508 | CX3CL1 | chr16 | 57372461 | 57385048 | 1.607762959 | 8.091220028 | 0.000257199 | 0.003284012 | 0.451612903 | 2.600496278 | Up |
| 1352 | ENSG00000006695 | K02257 | COX10 | chr17 | 14069402 | 14208679 | 1.775246972 | 8.2503882 | 0.0000263 | 0.000398915 | 0.358916479 | 3.002257336 | Up |
| 1951 | ENSG00000008300 | K04602 | CELSR3 | chr3 | 48636463 | 48662915 | 6.399958505 | 6.929028159 | 4.71E-10 | 1.33E-08 | 0.445954106 | 2.711050725 | Up |
| 23542 | ENSG00000008735 | K04435 | MAPK8IP2 | chr22 | 50600685 | 50613978 | 6.334928135 | 6.864374614 | 2.04E-09 | 5.73E-08 | 0.577669903 | 2.565533981 | Up |
| 1185 | ENSG00000011021 | K05015 | CLCN6 | chr1 | 11806096 | 11843144 | 7.19824637 | 7.722707285 | 6.78E-30 | 2.59E-26 | 0.421825813 | 3.008394544 | Up |
| 54903 | ENSG00000011143 | K19332 | MKS1 | chr17 | 58205436 | 58219605 | 1.622607776 | 8.105281353 | 0.000242105 | 0.003135256 | 0.540250447 | 3.114490161 | Up |
| 9052 | ENSG00000013588 | K08468 | GPRC5A | chr12 | 12891022 | 12913666 | 6.322679526 | 6.852467845 | 0.00000325 | 0.0000563 | 0.394957983 | 3.204481793 | Up |
| 27032 | ENSG00000017260 | K01537 | ATP2C1 | chr3 | 130850500 | 131016712 | 1.418205398 | 7.912299976 | 0.000820336 | 0.009190091 | 0.441932169 | 2.907502569 | Up |
| 7431 | ENSG00000026025 | K07606 | VIM | chr10 | 17227935 | 17237593 | 6.718280752 | 7.245329156 | 6.68E-19 | 4.29E-16 | 0.654506438 | 2.924892704 | Up |
| 11119 | ENSG00000026950 | K06712 | BTN3A1 | chr6 | 26402237 | 26415216 | 2.413383467 | 7.865127089 | 3.69E-08 | 0.000000943 | 0.452241715 | 3.033138402 | Up |
| 54477 | ENSG00000052126 | - | PLEKHA5 | chr12 | 19129680 | 19376400 | 6.571859389 | 7.099721885 | 8.24E-14 | 4.13E-12 | 0.569422777 | 2.972698908 | Up |
| 2928 | ENSG00000063515 | K09325 | GSC2 | chr22 | 19148991 | 19150283 | 6.761186284 | 7.287794938 | 3.65E-19 | 2.62E-16 | 0.27804878 | 2.52195122 | Up |
| 54763 | ENSG00000065371 | - | ROPN1 | chr3 | 123969006 | 123992170 | 6.496791697 | 7.025533135 | 0.000000411 | 0.00000982 | 0.462264151 | 3.033018868 | Up |
| 3028 | ENSG00000072506 | K08683 | HSD17B10 | chrX | 53431258 | 53434376 | 1.622607826 | 8.105281353 | 0.000242267 | 0.003135256 | 0.363984674 | 2.54789272 | Up |
| 8974 | ENSG00000072682 | K00472 | P4HA2 | chr5 | 132190147 | 132227863 | 2.814386348 | 7.218960683 | 0.000811646 | 0.009186899 | 0.519626168 | 3.059813084 | Up |
| 3993 | ENSG00000073350 | K06094 | LLGL2 | chr17 | 75525080 | 75575209 | 1.418205423 | 7.912299976 | 0.000820688 | 0.009190091 | 0.448039216 | 2.932352941 | Up |
| 27091 | ENSG00000075429 | K04870 | CACNG5 | chr17 | 66877273 | 66885278 | 6.322679729 | 6.852467845 | 0.00000324 | 0.0000562 | 0.429090909 | 3.087272727 | Up |
| 23505 | ENSG00000075568 | - | TMEM131 | chr2 | 97756336 | 97996182 | 2.417859481 | 7.869389482 | 0.000000155 | 0.0000038 | 0.481678173 | 2.842272969 | Up |
| 2191 | ENSG00000078098 | K08674 | FAP | chr2 | 162170684 | 162243535 | 6.756510335 | 7.282920267 | 2.19E-18 | 1.32E-15 | 0.513157895 | 3.255263158 | Up |
| 51150 | ENSG00000078808 | K19934 | SDF4 | chr1 | 1216908 | 1232067 | 1.847547824 | 7.320918262 | 0.000264101 | 0.003357188 | 0.591160221 | 3.063535912 | Up |
| 29843 | ENSG00000079387 | K08592 | SENP1 | chr12 | 48042893 | 48106308 | 2.26320809 | 7.719833801 | 6.92E-08 | 0.00000173 | 0.548136646 | 2.98757764 | Up |
| 51006 | ENSG00000080189 | K15280 | SLC35C2 | chr20 | 46349528 | 46364461 | 1.451844116 | 7.943939812 | 0.000920325 | 0.009936393 | 0.335025381 | 3.121827411 | Up |
| 55183 | ENSG00000080345 | K11138 | RIF1 | chr2 | 151409883 | 151534200 | 1.775247023 | 8.2503882 | 0.0000263 | 0.000398915 | 0.578478964 | 2.825242718 | Up |
| 1361 | ENSG00000080618 | K01300 | CPB2 | chr13 | 46053186 | 46105076 | 6.144248322 | 6.675180249 | 0.0000118 | 0.000191716 | 0.475177305 | 3.177304965 | Up |
| 174 | ENSG00000081051 | K16144 | AFP | chr4 | 73436219 | 73455785 | 5.576334612 | 6.111868284 | 0.000364594 | 0.004514514 | 0.522508039 | 2.998392283 | Up |
| 4986 | ENSG00000082556 | K04214 | OPRK1 | chr8 | 53225716 | 53251697 | 6.588038667 | 7.116247275 | 0.000000119 | 0.00000293 | 0.392105263 | 3.128947368 | Up |
| 29957 | ENSG00000085491 | K14684 | SLC25A24 | chr1 | 108134722 | 108200358 | 1.516810361 | 8.005212138 | 0.000474619 | 0.005737696 | 0.471698113 | 3.01048218 | Up |
| 6564 | ENSG00000088386 | K14206 | SLC15A1 | chr13 | 98683801 | 98752675 | 6.492350535 | 7.020885579 | 1E-11 | 2.91E-10 | 0.405367232 | 3.09180791 | Up |
| 1795 | ENSG00000088538 | K05727 | DOCK3 | chr3 | 50674969 | 51384198 | 6.301279446 | 6.830929145 | 4.3E-09 | 0.000000119 | 0.49408867 | 3.112315271 | Up |
| 54822 | ENSG00000092439 | K04982 | TRPM7 | chr15 | 50557158 | 50686835 | 1.451843895 | 7.943939812 | 0.000917121 | 0.009911134 | 0.474530831 | 3.105093834 | Up |
| 3326 | ENSG00000096384 | K04079 | HSP90AB1 | chr6 | 44246166 | 44253888 | 1.607762593 | 8.091220028 | 0.000255932 | 0.003271485 | 0.620165746 | 3.070441989 | Up |
| 27328 | ENSG00000099715 | K16498 | PCDH11X | chrY | 5000044 | 5742228 | 6.399958716 | 6.929028159 | 4.68E-10 | 1.32E-08 | 0.530597015 | 2.835820896 | Up |
| 4242 | ENSG00000100060 | K05948 | MFNG | chr22 | 37469063 | 37486440 | 1.418205448 | 7.912299976 | 0.00082104 | 0.009190091 | 0.38317757 | 3.040498442 | Up |
| 23464 | ENSG00000100116 | K00639 | GCAT | chr22 | 37807893 | 37817177 | 6.087972512 | 6.6192953 | 0.0000188 | 0.000300271 | 0.375280899 | 2.759550562 | Up |
| 25809 | ENSG00000100271 | K16599 | TTLL1 | chr22 | 43039516 | 43089428 | 1.775245757 | 8.2503882 | 0.0000259 | 0.000393306 | 0.508274232 | 3.191489362 | Up |
| 55007 | ENSG00000100376 | - | FAM118A | chr22 | 45309200 | 45341955 | 6.244182653 | 6.77447263 | 0.0000064 | 0.000108336 | 0.509803922 | 2.960784314 | Up |
| 161394 | ENSG00000100583 | - | SAMD15 | chr14 | 77377419 | 77391244 | 1.487617083 | 7.977683044 | 0.000444139 | 0.005414853 | 0.740356083 | 2.96735905 | Up |
| 623 | ENSG00000100739 | K03915 | BDKRB1 | chr14 | 96256210 | 96264763 | 6.322299742 | 6.851514067 | 2.45E-09 | 6.86E-08 | 0.322946176 | 3.354107649 | Up |
| 28316 | ENSG00000101542 | K06807 | CDH20 | chr18 | 61333582 | 61555773 | 6.408936026 | 6.937770834 | 1.43E-10 | 4.07E-09 | 0.553058677 | 2.907615481 | Up |
| 284217 | ENSG00000101680 | K05637 | LAMA1 | chr18 | 6941744 | 7117814 | 6.785568827 | 7.3118627 | 6.74E-19 | 4.29E-16 | 0.492357724 | 2.735934959 | Up |
| 2010 | ENSG00000102119 | K12569 | EMD | chrX | 154379237 | 154381523 | 1.703045751 | 7.737122081 | 0.00007 | 0.000991691 | 0.614173228 | 3.023622047 | Up |
| 5595 | ENSG00000102882 | K04371 | MAPK3 | chr16 | 30114105 | 30123309 | 1.516810389 | 8.005212138 | 0.000474811 | 0.005737696 | 0.477572559 | 3.09762533 | Up |
| 100507577 | ENSG00000102910 | - | LOC100507577 | chr16 | 48355685 | 48362999 | 5.927193098 | 6.459712583 | 0.0000534 | 0.000759321 | 0.460093897 | 2.91314554 | Up |
| 80004 | ENSG00000103067 | K14947 | ESRP2 | chr16 | 68228547 | 68236233 | 1.418205485 | 7.912299976 | 0.000821546 | 0.009190091 | 0.412654746 | 2.800550206 | Up |
| 10273 | ENSG00000103266 | K09561 | STUB1 | chr16 | 680111 | 682768 | 2.220172401 | 8.232505304 | 0.000000892 | 0.0000207 | 0.561056106 | 2.98019802 | Up |
| 50615 | ENSG00000103522 | K05075 | IL21R | chr16 | 27402162 | 27452045 | 6.399959057 | 6.929028159 | 4.63E-10 | 1.31E-08 | 0.488847584 | 2.916356877 | Up |
| 65263 | ENSG00000104524 | K00286 | PYCRL | chr8 | 143603913 | 143609614 | 2.067582894 | 8.085492466 | 0.0000124 | 0.000200494 | 0.346153846 | 2.534965035 | Up |
| 7993 | ENSG00000104691 | - | UBXN8 | chr8 | 30729131 | 30767006 | 1.622607017 | 8.105281353 | 0.000239653 | 0.003120225 | 0.496774194 | 3.009677419 | Up |
| 79852 | ENSG00000105131 | - | EPHX3 | chr19 | 15226919 | 15233047 | 6.468072876 | 6.996541626 | 1.29E-11 | 3.73E-10 | 0.375 | 3.213888889 | Up |
| 898 | ENSG00000105173 | K06626 | CCNE1 | chr19 | 29811994 | 29824317 | 5.658157865 | 6.19284195 | 0.0000365 | 0.000548399 | 0.497560976 | 3.085365854 | Up |
| 27113 | ENSG00000105327 | K10132 | BBC3 | chr19 | 47220822 | 47232998 | 3.027747098 | 7.427673732 | 0.0000388 | 0.000578676 | 0.218390805 | 2.325670498 | Up |
| 83743 | ENSG00000105447 | K14848 | GRWD1 | chr19 | 48445773 | 48453907 | 6.179184555 | 6.710140096 | 0.000154699 | 0.00205536 | 0.51793722 | 2.83632287 | Up |
| 23770 | ENSG00000105701 | K09574 | FKBP8 | chr19 | 18531752 | 18544077 | 1.418205273 | 7.912299976 | 0.000818617 | 0.009190091 | 0.491525424 | 2.755447942 | Up |
| 27077 | ENSG00000108641 | K16744 | B9D1 | chr17 | 19335372 | 19378193 | 1.539751282 | 8.026863556 | 0.000514032 | 0.005942872 | 0.281690141 | 2.690140845 | Up |
| 40 | ENSG00000108684 | K04828 | ASIC2 | chr17 | 33013087 | 34156806 | 7.202970114 | 7.727673401 | 2.53E-24 | 3.23E-21 | 0.440497336 | 2.969804618 | Up |
| 5007 | ENSG00000110048 | K20456 | OSBP | chr11 | 59574398 | 59616144 | 1.451794547 | 7.943939812 | 0.000422374 | 0.005194355 | 0.505576208 | 2.828996283 | Up |
| 28960 | ENSG00000110063 | K12584 | DCPS | chr11 | 126303301 | 126345754 | 1.590014037 | 8.074423544 | 0.000253166 | 0.003259292 | 0.507418398 | 3.112759644 | Up |
| 79080 | ENSG00000110104 | K14822 | CCDC86 | chr11 | 60841956 | 60851088 | 1.273272687 | 7.776570932 | 0.000810365 | 0.009186899 | 0.477777778 | 2.813888889 | Up |
| 2305 | ENSG00000111206 | K09406 | FOXM1 | chr12 | 2857681 | 2877155 | 2.095071856 | 7.557797848 | 0.000000111 | 0.00000275 | 0.489388265 | 2.885143571 | Up |
| 2967 | ENSG00000111358 | K03143 | GTF2H3 | chr12 | 123633739 | 123662604 | 1.853847992 | 7.940202425 | 0.000441178 | 0.005398657 | 0.444805195 | 2.99025974 | Up |
| 2651 | ENSG00000111846 | K00742 | GCNT2 | chr6 | 10521283 | 10629368 | 1.418205322 | 7.912299976 | 0.000819285 | 0.009190091 | 0.462686567 | 3.176616915 | Up |
| 222658 | ENSG00000112078 | - | KCTD20 | chr6 | 36442767 | 36491143 | 6.391928652 | 6.921278639 | 0.00000158 | 0.0000317 | 0.548926014 | 2.971360382 | Up |
| 4224 | ENSG00000112818 | K01395 | MEP1A | chr6 | 46793357 | 46845987 | 6.027606651 | 6.559092149 | 0.00000029 | 0.00000703 | 0.515503876 | 3.059431525 | Up |
| 55374 | ENSG00000113119 | - | TMCO6 | chr5 | 140596529 | 140647411 | 1.590014103 | 8.074423544 | 0.000253394 | 0.003259292 | 0.436873747 | 2.885771543 | Up |
| 9421 | ENSG00000113196 | K09071 | HAND1 | chr5 | 154474972 | 154478264 | 5.924705635 | 6.457426848 | 0.000478667 | 0.005771187 | 0.372093023 | 2.865116279 | Up |
| 735 | ENSG00000113600 | K04000 | C9 | chr5 | 39284275 | 39364553 | 6.337654267 | 6.867377057 | 0.000003 | 0.0000521 | 0.5509839 | 2.932021467 | Up |
| 152015 | ENSG00000114547 | - | ROPN1B | chr3 | 125969144 | 125983454 | 6.496791967 | 7.025533135 | 0.000000409 | 0.00000981 | 0.45754717 | 3.075471698 | Up |
| 4820 | ENSG00000114857 | K12740 | NKTR | chr3 | 42600655 | 42648741 | 1.896798098 | 7.921790452 | 0.0000452 | 0.00067185 | 0.603283174 | 2.755813953 | Up |
| 54760 | ENSG00000115257 | K08671 | PCSK4 | chr19 | 1481428 | 1490874 | 7.302295596 | 7.826657813 | 6.2E-28 | 1.42E-24 | 0.417218543 | 2.814569536 | Up |
| 64427 | ENSG00000115282 | - | TTC31 | chr2 | 74483032 | 74494564 | 2.516460821 | 7.96501676 | 8.64E-08 | 0.00000215 | 0.458574181 | 2.791907514 | Up |
| 55825 | ENSG00000115425 | K07753 | PECR | chr2 | 216029088 | 216081823 | 1.487617111 | 7.977683044 | 0.00044432 | 0.005414853 | 0.409240924 | 2.801980198 | Up |
| 8809 | ENSG00000115604 | K05173 | IL18R1 | chr2 | 102355817 | 102398777 | 1.516810417 | 8.005212138 | 0.000475004 | 0.005737696 | 0.487985213 | 3.171903882 | Up |
| 51253 | ENSG00000116221 | K17418 | MRPL37 | chr1 | 54200167 | 54225489 | 1.4182051 | 7.912299976 | 0.000816222 | 0.009190091 | 0.395445135 | 3.126293996 | Up |
| 79971 | ENSG00000116729 | - | WLS | chr1 | 68098459 | 68233000 | 6.144239204 | 6.675180249 | 0.0000138 | 0.000223297 | 0.370165746 | 3.314917127 | Up |
| 78989 | ENSG00000118004 | K10066 | COLEC11 | chr2 | 3594832 | 3644644 | 6.146521403 | 6.677150399 | 4.46E-08 | 0.00000113 | 0.438596491 | 2.571929825 | Up |
| 6909 | ENSG00000121068 | K10176 | TBX2 | chr17 | 61399896 | 61409466 | 1.539751326 | 8.026863556 | 0.000514354 | 0.005942872 | 0.386235955 | 2.657303371 | Up |
| 9227 | ENSG00000121207 | K00678 | LRAT | chr4 | 154740841 | 154753119 | 6.745469981 | 7.272843403 | 1.04E-08 | 0.000000269 | 0.430434783 | 3.047826087 | Up |
| 5290 | ENSG00000121879 | K00922 | PIK3CA | chr3 | 179148114 | 179240093 | 1.516810446 | 8.005212138 | 0.000475196 | 0.005737696 | 0.47659176 | 3.200374532 | Up |
| 9956 | ENSG00000122254 | K07808 | HS3ST2 | chr16 | 22814162 | 22916338 | 6.091386424 | 6.622852149 | 0.000248236 | 0.003208882 | 0.395095368 | 3.073569482 | Up |
| 127495 | ENSG00000122477 | - | LRRC39 | chr1 | 100148448 | 100178273 | 6.448305597 | 6.977077733 | 3.45E-11 | 9.93E-10 | 0.507374631 | 3.147492625 | Up |
| 170685 | ENSG00000122824 | K07766 | NUDT10 | chrX | 51332231 | 51337525 | 5.927193218 | 6.459712583 | 0.0000534 | 0.0007591 | 0.530487805 | 2.945121951 | Up |
| 5033 | ENSG00000122884 | K00472 | P4HA1 | chr10 | 73007217 | 73096974 | 1.622607017 | 8.105281353 | 0.000239651 | 0.003120225 | 0.533707865 | 3.108614232 | Up |
| 9559 | ENSG00000122958 | K18466 | VPS26A | chr10 | 69124153 | 69174416 | 1.607762234 | 8.091220028 | 0.000254694 | 0.003259292 | 0.525993884 | 3.281345566 | Up |
| 976 | ENSG00000123146 | K08446 | ADGRE5 | chr19 | 14381144 | 14408725 | 6.017998498 | 6.549996626 | 0.000330192 | 0.004128659 | 0.445508982 | 2.841916168 | Up |
| 4778 | ENSG00000123405 | K09039 | NFE2 | chr12 | 54292107 | 54301037 | 5.978154336 | 6.50999974 | 0.000000513 | 0.0000121 | 0.54155496 | 2.879356568 | Up |
| 64756 | ENSG00000123472 | K07555 | ATPAF1 | chr1 | 46635039 | 46668427 | 6.01744101 | 6.548983531 | 0.000000609 | 0.0000142 | 0.478632479 | 2.891737892 | Up |
| 6906 | ENSG00000123561 | K20734 | SERPINA7 | chrX | 106032439 | 106038727 | 5.862142385 | 6.394967786 | 0.00000298 | 0.0000519 | 0.472289157 | 3.031325301 | Up |
| 203447 | ENSG00000123572 | K16313 | NRK | chrX | 105821944 | 105958610 | 6.156354189 | 6.686931574 | 2.96E-08 | 0.000000757 | 0.538558786 | 2.928571429 | Up |
| 164781 | ENSG00000123977 | K19760 | DAW1 | chr2 | 227871054 | 227924347 | 6.404725951 | 6.934000898 | 0.00000143 | 0.0000288 | 0.506024096 | 2.853012048 | Up |
| 57580 | ENSG00000124126 | K12365 | PREX1 | chr20 | 48624252 | 48827883 | 5.658157896 | 6.19284195 | 0.0000364 | 0.000547716 | 0.50331525 | 2.927667269 | Up |
| 1088 | ENSG00000124469 | K06499 | CEACAM8 | chr19 | 42580243 | 42595016 | 7.144046452 | 7.67036493 | 0.00000369 | 0.0000634 | 0.501432665 | 2.808022923 | Up |
| 54210 | ENSG00000124731 | K14362 | TREM1 | chr6 | 41267385 | 41286745 | 6.32478642 | 6.854542553 | 0.00000328 | 0.0000567 | 0.488 | 3.032 | Up |
| 9271 | ENSG00000125207 | K02156 | PIWIL1 | chr12 | 130337887 | 130412708 | 5.734885391 | 6.268998612 | 0.000154002 | 0.002048471 | 0.459930314 | 3.083623693 | Up |
| 6662 | ENSG00000125398 | K18435 | SOX9 | chr17 | 72121020 | 72126420 | 2.160847768 | 8.234632346 | 0.00000332 | 0.0000573 | 0.532416503 | 2.823182711 | Up |
| 128408 | ENSG00000125533 | K09086 | BHLHE23 | chr20 | 63005979 | 63007035 | 6.304077617 | 6.834001846 | 0.00000414 | 0.0000707 | 0.365145228 | 2.502074689 | Up |
| 10236 | ENSG00000125944 | K13161 | HNRNPR | chr1 | 23304688 | 23344650 | 1.685602099 | 8.165085747 | 0.0000868 | 0.001220798 | 0.518867925 | 2.919811321 | Up |
| 23769 | ENSG00000126500 | K16362 | FLRT1 | chr11 | 64035970 | 64119183 | 5.827986524 | 6.36129966 | 0.000105443 | 0.001442735 | 0.482195846 | 2.841246291 | Up |
| 84286 | ENSG00000127419 | - | TMEM175 | chr4 | 932387 | 958656 | 5.968214407 | 6.500591737 | 0.000402386 | 0.004961034 | 0.33531746 | 3.061507937 | Up |
| 7429 | ENSG00000127831 | K05761 | VIL1 | up regulated proteins |  |  | 6.040404912 | 6.572241501 | 0.000321432 | 0.004027913 | 0.500604595 | 2.978234583 | Up |
| 8854 | ENSG00000128918 | K07249 | ALDH1A2 | chr15 | 57953424 | 58065923 | 6.144239386 | 6.675180249 | 0.0000138 | 0.000222917 | 0.445945946 | 2.88996139 | Up |
| 25983 | ENSG00000129460 | K14765 | NGDN | chr14 | 23469689 | 23478193 | 5.652753536 | 6.187596075 | 0.000254402 | 0.003259292 | 0.526984127 | 2.946031746 | Up |
| 10038 | ENSG00000129484 | K10798 | PARP2 | chr14 | 20343041 | 20357904 | 6.204874325 | 6.735570678 | 0.0001514 | 0.002016207 | 0.492281304 | 2.982847341 | Up |
| 547 | ENSG00000130294 | K10392 | KIF1A | chr2 | 240713764 | 240821025 | 6.573910419 | 7.102272984 | 0.000000117 | 0.00000288 | 0.546621999 | 2.938581798 | Up |
| 25796 | ENSG00000130313 | K01057 | PGLS | chr19 | 17511623 | 17521291 | 1.451794591 | 7.943939812 | 0.00042267 | 0.005194355 | 0.379844961 | 2.80620155 | Up |
| 64793 | ENSG00000130695 | K16766 | CEP85 | chr1 | 26234153 | 26278810 | 6.231241083 | 6.761810607 | 0.000129401 | 0.001749656 | 0.577427822 | 2.830708661 | Up |
| 445 | ENSG00000130707 | K01940 | ASS1 | chr9 | 130444707 | 130501274 | 6.949243867 | 7.475839235 | 4.04E-09 | 0.000000113 | 0.475728155 | 3.067961165 | Up |
| 84106 | ENSG00000133246 | K19994 | PRAM1 | chr19 | 8490055 | 8502654 | 6.304077727 | 6.834001846 | 0.00000413 | 0.0000706 | 0.425373134 | 2.958208955 | Up |
| 84953 | ENSG00000133808 | - | MICALCL | chr11 | 12286900 | 12359144 | 6.156354562 | 6.686931574 | 2.93E-08 | 0.000000752 | 0.53381295 | 2.827338129 | Up |
| 27255 | ENSG00000134115 | K06764 | CNTN6 | chr3 | 978021 | 1403610 | 6.151134682 | 6.682228769 | 0.000162062 | 0.002148217 | 0.500972763 | 2.944552529 | Up |
| 10956 | ENSG00000135506 | K10088 | OS9 | chr12 | 57693955 | 57721557 | 1.673984781 | 8.154059542 | 0.0000882 | 0.001236909 | 0.5982009 | 2.932533733 | Up |
| 2016 | ENSG00000135638 | K09317 | EMX1 | chr2 | 72911147 | 72936691 | 6.576633127 | 7.104705157 | 1.66E-13 | 7.37E-12 | 0.351724138 | 2.789655172 | Up |
| 64114 | ENSG00000135926 | K06890 | TMBIM1 | chr2 | 218274192 | 218292577 | 1.418205138 | 7.912299976 | 0.000816757 | 0.009190091 | 0.363344051 | 3.196141479 | Up |
| 26122 | ENSG00000135999 | K11322 | EPC2 | chr2 | 148644780 | 148788491 | 1.685601655 | 8.165085747 | 0.0000863 | 0.001217422 | 0.542750929 | 2.861214374 | Up |
| 10643 | ENSG00000136231 | K13197 | IGF2BP3 | chr7 | 23310209 | 23470674 | 6.914250894 | 7.439473038 | 5.98E-26 | 9.8E-23 | 0.469775475 | 2.865284974 | Up |
| 57538 | ENSG00000136383 | K08868 | ALPK3 | chr15 | 84816631 | 84873482 | 2.622261608 | 8.067815926 | 4.95E-08 | 0.00000126 | 0.473518616 | 2.520713162 | Up |
| 10541 | ENSG00000136938 | K18647 | ANP32B | chr9 | 97983207 | 98015943 | 2.357892395 | 7.811314164 | 0.0000003 | 0.00000723 | 0.892430279 | 2.848605578 | Up |
| 27283 | ENSG00000137251 | - | TINAG | chr6 | 54307792 | 54390152 | 6.337665481 | 6.867377057 | 0.00000232 | 0.0000417 | 0.50210084 | 3.021008403 | Up |
| 8870 | ENSG00000137331 | - | IER3 | chr6 | 30743199 | 30744550 | 1.703181601 | 7.737122081 | 0.000376394 | 0.004655595 | 0.365384615 | 2.852564103 | Up |
| 51050 | ENSG00000137558 | - | PI15 | chr8 | 74824537 | 74855044 | 6.130377395 | 6.661629243 | 0.000208893 | 0.002746757 | 0.426356589 | 3.007751938 | Up |
| 676 | ENSG00000137948 | K11724 | BRDT | chr1 | 91949371 | 92014428 | 6.98382126 | 7.508904127 | 6.55E-25 | 9.38E-22 | 0.575184017 | 2.94637224 | Up |
| 5861 | ENSG00000138069 | K07874 | RAB1A | chr2 | 65086854 | 65130301 | 1.622606125 | 8.105281353 | 0.000236799 | 0.003092411 | 0.536585366 | 2.87804878 | Up |
| 10152 | ENSG00000138443 | K05751 | ABI2 | chr2 | 203327874 | 203432173 | 6.07564446 | 6.607046607 | 0.0000181 | 0.000290705 | 0.514619883 | 2.77582846 | Up |
| 144193 | ENSG00000139344 | K01468 | AMDHD1 | chr12 | 95943293 | 95968592 | 7.352192777 | 7.876699933 | 1.3E-22 | 1.35E-19 | 0.424882629 | 2.814553991 | Up |
| 83851 | ENSG00000139973 | K19328 | SYT16 | chr14 | 61812156 | 62112825 | 6.492350814 | 7.020885579 | 9.9E-12 | 2.88E-10 | 0.595348837 | 2.810852713 | Up |
| 8816 | ENSG00000139990 | K11800 | DCAF5 | chr14 | 69050920 | 69153393 | 1.418205177 | 7.912299976 | 0.000817292 | 0.009190091 | 0.579617834 | 2.715498938 | Up |
| 8125 | ENSG00000140350 | K18646 | ANP32A | chr15 | 68778535 | 68820922 | 2.357892413 | 7.811314164 | 0.0000003 | 0.00000723 | 0.895582329 | 2.843373494 | Up |
| 8826 | ENSG00000140575 | K16848 | IQGAP1 | chr15 | 90388241 | 90502243 | 1.775244349 | 8.2503882 | 0.0000254 | 0.000387053 | 0.53952927 | 3.056125528 | Up |
| 7385 | ENSG00000140740 | K00415 | UQCRC2 | chr16 | 21953064 | 21983660 | 1.903789381 | 7.375029389 | 0.0000244 | 0.000372423 | 0.437086093 | 2.748344371 | Up |
| 6760 | ENSG00000141380 | K15623 | SS18 | chr18 | 26016253 | 26091217 | 6.411360952 | 6.940624335 | 0.00000128 | 0.0000258 | 0.523923445 | 2.677033493 | Up |
| 402665 | ENSG00000142549 | K06773 | IGLON5 | chr19 | 51311848 | 51330848 | 5.956595412 | 6.488953942 | 0.0000759 | 0.001074502 | 0.443452381 | 2.803571429 | Up |
| 387597 | ENSG00000143195 | - | ILDR2 | chr1 | 166913204 | 166975324 | 6.090322727 | 6.621342101 | 0.000000122 | 0.00000301 | 0.491392801 | 2.849765258 | Up |
| 81611 | ENSG00000143401 | K18648 | ANP32E | chr1 | 150218417 | 150236156 | 2.357892424 | 7.811314164 | 0.0000003 | 0.00000723 | 0.940298507 | 2.809701493 | Up |
| 7170 | ENSG00000143549 | K09290 | TPM3 | chr1 | 154155304 | 154192135 | 1.700953326 | 8.179657537 | 0.0000911 | 0.00127008 | 0.712280702 | 2.943859649 | Up |
| 148327 | ENSG00000143578 | K09048 | CREB3L4 | chr1 | 153967679 | 153974364 | 1.418205216 | 7.912299976 | 0.000817828 | 0.009190091 | 0.506329114 | 2.794936709 | Up |
| 1944 | ENSG00000143590 | K05462 | EFNA3 | chr1 | 155078872 | 155087538 | 6.811304624 | 7.337061587 | 2.91E-21 | 2.78E-18 | 0.382352941 | 3.008403361 | Up |
| 9027 | ENSG00000144035 | K20838 | NAT8 | chr2 | 73640723 | 73642410 | 6.204874463 | 6.735570678 | 0.000151032 | 0.002013638 | 0.374449339 | 3.145374449 | Up |
| 7220 | ENSG00000144935 | K04964 | TRPC1 | chr3 | 142724032 | 142807888 | 1.694574641 | 8.173603826 | 0.0000869 | 0.001220798 | 0.469104666 | 3.187894073 | Up |
| 64839 | ENSG00000145743 | K10283 | FBXL17 | chr5 | 107859033 | 108382098 | 6.658682634 | 7.186331686 | 1.11E-15 | 2.7E-13 | 0.410841655 | 2.67189729 | Up |
| 114898 | ENSG00000145861 | - | C1QTNF2 | chr5 | 160347558 | 160370656 | 1.700953372 | 8.179657537 | 0.0000912 | 0.00127008 | 0.366666667 | 2.651515152 | Up |
| 85417 | ENSG00000147082 | K05868 | CCNB3 | chrX | 50202713 | 50351914 | 5.658157925 | 6.19284195 | 0.0000363 | 0.000547147 | 0.564157706 | 3.035842294 | Up |
| 84675 | ENSG00000147573 | K10654 | TRIM55 | chr8 | 66112667 | 66175485 | 6.340515723 | 6.870373272 | 0.0000797 | 0.001124841 | 0.580291971 | 2.770072993 | Up |
| 4288 | ENSG00000148773 | K17582 | MKI67 | chr10 | 128096659 | 128126405 | 2.539395559 | 7.98727545 | 0.000000107 | 0.00000266 | 0.536855037 | 2.749078624 | Up |
| 83707 | ENSG00000149743 | K10669 | TRPT1 | chr11 | 64223799 | 64226254 | 1.451843721 | 7.943939812 | 0.000914601 | 0.009893222 | 0.368627451 | 2.843137255 | Up |
| 27287 | ENSG00000151650 | - | VENTX | chr10 | 133237904 | 133241930 | 6.658682822 | 7.186331686 | 1.09E-15 | 2.7E-13 | 0.38372093 | 2.651162791 | Up |
| 9425 | ENSG00000153046 | K00653 | CDYL | chr6 | 4706159 | 4955544 | 1.451843497 | 7.943939812 | 0.000911383 | 0.009867724 | 0.486622074 | 2.851170569 | Up |
| 154043 | ENSG00000153721 | - | CNKSR3 | chr6 | 154405299 | 154510619 | 1.45184327 | 7.943939812 | 0.000908118 | 0.009841661 | 0.529477197 | 2.901001112 | Up |
| 123872 | ENSG00000154099 | K19750 | DNAAF1 | chr16 | 84145260 | 84178761 | 6.766140269 | 7.293010449 | 2.62E-19 | 2.27E-16 | 0.604137931 | 2.762758621 | Up |
| 23043 | ENSG00000154310 | K08840 | TNIK | chr3 | 171058414 | 171460408 | 1.638726503 | 8.120466352 | 0.000827134 | 0.009243587 | 0.552941176 | 2.948529412 | Up |
| 115362 | ENSG00000154451 | K20898 | GBP5 | chr1 | 89258950 | 89272861 | 1.836092886 | 7.310304875 | 0.00028289 | 0.003570862 | 0.544368601 | 3.008532423 | Up |
| 118813 | ENSG00000155256 | K19368 | ZFYVE27 | chr10 | 97737121 | 97760907 | 6.304077837 | 6.834001846 | 0.00000412 | 0.0000706 | 0.5 | 2.896634615 | Up |
| 57188 | ENSG00000156218 | - | ADAMTSL3 | chr15 | 83654086 | 84039842 | 5.695739126 | 6.230182532 | 0.000208083 | 0.002742391 | 0.46481372 | 2.813719692 | Up |
| 29104 | ENSG00000156239 | K19589 | N6AMT1 | chr21 | 28571095 | 28885373 | 6.571859559 | 7.099721885 | 8.2E-14 | 4.13E-12 | 0.425233645 | 2.803738318 | Up |
| 7074 | ENSG00000156299 | K05731 | TIAM1 | chr21 | 31118418 | 31559977 | 6.475675717 | 7.004612975 | 0.000000614 | 0.0000143 | 0.548082967 | 2.857950974 | Up |
| 159090 | ENSG00000156504 | - | FAM122B | chrX | 134769566 | 134797232 | 5.918898554 | 6.451498269 | 0.0000536 | 0.000761296 | 0.546816479 | 2.666666667 | Up |
| 5.75E+04 | ENSG00000156564 | K16355 | LRFN2 | chr6 | 40391589 | 40587464 | 7.089953352 | 7.61E+00 | 2.08E-31 | 2.39E-27 | 0.434727503 | 2.730038023 | Up |
| 491 | ENSG00000157087 | K05850 | ATP2B2 | chr3 | 10324023 | 10707962 | 5.47831626 | 6.014786728 | 0.000566408 | 0.006520517 | 0.477876106 | 2.871279163 | Up |
| 776 | ENSG00000157388 | K04851 | CACNA1D | chr3 | 53495049 | 53813151 | 1.877124863 | 7.349486626 | 0.000156166 | 0.002072455 | 0.469050894 | 3.079779917 | Up |
| 909 | ENSG00000158477 | K06448 | CD1A | up regulated proteins |  |  | 6.083095547 | 6.614446438 | 0.0000179 | 0.000288006 | 0.418960245 | 3.137614679 | Up |
| 29980 | ENSG00000159147 | - | DONSON | chr21 | 33577551 | 33588708 | 6.373414533 | 6.90292782 | 0.00000209 | 0.0000389 | 0.454063604 | 2.931095406 | Up |
| 1487 | ENSG00000159692 | K04496 | CTBP1 | chr4 | 1211440 | 1250329 | 1.418205255 | 7.912299976 | 0.000818365 | 0.009190091 | 0.409090909 | 2.752272727 | Up |
| 27151 | ENSG00000160111 | - | CPAMD8 | chr19 | 16892951 | 17026818 | 6.027854328 | 6.559838416 | 0.000296093 | 0.003726681 | 0.44047619 | 2.861283644 | Up |
| 6285 | ENSG00000160307 | - | S100B | chr21 | 46598618 | 46605242 | 5.71062871 | 6.245159976 | 0.000857262 | 0.009561637 | 0.457446809 | 2.787234043 | Up |
| 115352 | ENSG00000160856 | K06727 | FCRL3 | chr1 | 157676481 | 157700985 | 6.57185973 | 7.099721885 | 8.15E-14 | 4.13E-12 | 0.490566038 | 2.834231806 | Up |
| 9794 | ENSG00000161021 | K06061 | MAML1 | chr5 | 179732850 | 179777286 | 1.775244467 | 8.2503882 | 0.0000254 | 0.000387143 | 0.475393701 | 2.572834646 | Up |
| 114770 | ENSG00000161031 | K01446 | PGLYRP2 | chr19 | 15468645 | 15479504 | 3.572206814 | 8.127699122 | 0.00000535 | 0.0000907 | 0.397476341 | 2.749211356 | Up |
| 246 | ENSG00000161905 | K00460 | ALOX15 | chr17 | 4630919 | 4641665 | 6.147529699 | 6.678602321 | 0.000181664 | 0.002402491 | 0.429003021 | 3.080060423 | Up |
| 81570 | ENSG00000162129 | K03695 | CLPB | chr11 | 72292425 | 72434684 | 1.451843042 | 7.943939812 | 0.000904858 | 0.009815611 | 0.473314607 | 2.912921348 | Up |
| 65260 | ENSG00000162377 | K18180 | COA7 | chr1 | 52686342 | 52698366 | 1.67398485 | 8.154059542 | 0.0000882 | 0.001236909 | 0.502164502 | 2.822510823 | Up |
| 116362 | ENSG00000162444 | - | RBP7 | chr1 | 9997197 | 10016020 | 6.228209677 | 6.758580738 | 0.00000655 | 0.000110729 | 0.514925373 | 3.111940299 | Up |
| 81493 | ENSG00000162520 | K10377 | SYNC | chr1 | 32679906 | 32702760 | 2.15241831 | 7.613362257 | 0.00000062 | 0.0000145 | 0.668049793 | 2.919087137 | Up |
| 6282 | ENSG00000163191 | - | S100A11 | chr1 | 152032506 | 152037035 | 1.513371464 | 7.61689979 | 0.000776118 | 0.008810863 | 0.523809524 | 2.933333333 | Up |
| 2560 | ENSG00000163288 | K05181 | GABRB1 | chr4 | 47031278 | 47438409 | 2.14442211 | 7.605335985 | 5.99E-08 | 0.0000015 | 0.481012658 | 3.158227848 | Up |
| 29851 | ENSG00000163600 | K06713 | ICOS | chr2 | 203936748 | 203961577 | 5.695739159 | 6.230182532 | 0.000207971 | 0.002742391 | 0.376884422 | 3.211055276 | Up |
| 29083 | ENSG00000163607 | - | GTPBP8 | chr3 | 112989275 | 113001966 | 1.451842824 | 7.943939812 | 0.000901739 | 0.009791041 | 0.407166124 | 3.153094463 | Up |
| 23001 | ENSG00000163625 | - | WDFY3 | chr4 | 84669537 | 84966391 | 1.487617138 | 7.977683044 | 0.0004445 | 0.005414853 | 0.478729438 | 2.979580261 | Up |
| 9204 | ENSG00000163867 | - | ZMYM6 | chr1 | 34986165 | 35031968 | 1.961495552 | 7.429589374 | 0.000048 | 0.000699486 | 0.489811321 | 2.918490566 | Up |
| 23272 | ENSG00000163946 | - | FAM208A | chr3 | 56620132 | 56683242 | 1.607761861 | 8.091220028 | 0.000253419 | 0.003259292 | 0.579241765 | 2.983219391 | Up |
| 91942 | ENSG00000164182 | K18160 | NDUFAF2 | chr5 | 60945129 | 61153037 | 1.451842604 | 7.943939812 | 0.000898625 | 0.009774872 | 0.562130178 | 3.218934911 | Up |
| 3360 | ENSG00000164270 | K04160 | HTR4 | chr5 | 148451032 | 148654527 | 6.704943118 | 7.232579484 | 2.69E-08 | 0.000000693 | 0.380841121 | 3.121495327 | Up |
| 115004 | ENSG00000164430 | K17834 | MB21D1 | chr6 | 73423711 | 73452330 | 1.700953419 | 8.179657537 | 0.0000912 | 0.00127008 | 0.455938697 | 2.982758621 | Up |
| 79870 | ENSG00000164929 | - | BAALC | chr8 | 103140693 | 103230305 | 6.729830361 | 7.256825487 | 2.95E-19 | 2.27E-16 | 0.55 | 2.516666667 | Up |
| 318 | ENSG00000164978 | K01518 | NUDT2 | chr9 | 34329506 | 34343713 | 6.241254824 | 6.771270514 | 1.22E-08 | 0.000000315 | 0.489795918 | 3.102040816 | Up |
| 80318 | ENSG00000165113 | - | GKAP1 | chr9 | 83739421 | 83817837 | 6.301279871 | 6.830929145 | 4.25E-09 | 0.000000118 | 0.620218579 | 2.909836066 | Up |
| 65268 | ENSG00000165238 | K08867 | WNK2 | chr9 | 93184156 | 93327581 | 2.516460328 | 7.96501676 | 8.48E-08 | 0.00000211 | 0.436221158 | 2.652154985 | Up |
| 139324 | ENSG00000165259 | - | HDX | chrX | 84317874 | 84502578 | 1.700953465 | 8.179657537 | 0.0000913 | 0.00127008 | 0.595652174 | 2.789855072 | Up |
| 7253 | ENSG00000165409 | K04249 | TSHR | chr14 | 80954989 | 81146302 | 5.576334697 | 6.111868284 | 0.000363951 | 0.004511415 | 0.460732984 | 3.158376963 | Up |
| 5015 | ENSG00000165588 | K18490 | OTX2 | chr14 | 56800707 | 56810476 | 6.087972737 | 6.6192953 | 0.0000187 | 0.000299624 | 0.501683502 | 2.744107744 | Up |
| 23203 | ENSG00000165688 | K01412 | PMPCA | chr9 | 136410573 | 136423761 | 1.418205294 | 7.912299976 | 0.000818903 | 0.009190091 | 0.455238095 | 2.899047619 | Up |
| 4745 | ENSG00000165973 | - | NELL1 | chr11 | 20669551 | 21575686 | 6.42291017 | 6.951838985 | 1.21E-10 | 3.47E-09 | 0.479713604 | 2.690930788 | Up |
| 11245 | ENSG00000166073 | K08442 | GPR176 | chr15 | 39795049 | 39920933 | 1.574916462 | 8.060056865 | 0.00089117 | 0.009774872 | 0.40776699 | 3.033009709 | Up |
| 8642 | ENSG00000166341 | K16507 | DCHS1 | chr11 | 6621324 | 6655849 | 2.145902299 | 8.160869841 | 0.00000364 | 0.0000625 | 0.467252881 | 2.61552456 | Up |
| 50810 | ENSG00000166503 | - | HDGFRP3 | chr15 | 83112738 | 83208018 | 1.914796174 | 7.38517521 | 0.0000336 | 0.000507392 | 0.620689655 | 2.807881773 | Up |
| 4632 | ENSG00000168530 | K05738 | MYL1 | chr2 | 210290144 | 210315171 | 5.970630698 | 6.502811204 | 0.00004 | 0.000595286 | 0.505154639 | 2.809278351 | Up |
| 55308 | ENSG00000168872 | K18655 | DDX19A | chr16 | 70346829 | 70373383 | 6.431609518 | 6.960716872 | 0.00000128 | 0.0000259 | 0.516736402 | 2.958158996 | Up |
| 4144 | ENSG00000168906 | K00789 | MAT2A | chr2 | 85538978 | 85545280 | 6.233338674 | 6.763667215 | 0.00000535 | 0.0000907 | 0.473417722 | 2.908860759 | Up |
| 9758 | ENSG00000169933 | - | FRMPD4 | chrX | 12137329 | 12724523 | 6.658682978 | 7.186331686 | 1.08E-15 | 2.7E-13 | 0.54311649 | 2.76172466 | Up |
| 2298 | ENSG00000170122 | K09397 | FOXD4 | chr9 | 116231 | 118417 | 6.260493157 | 6.790377599 | 6.55E-09 | 0.000000169 | 0.391799544 | 2.708428246 | Up |
| 3851 | ENSG00000170477 | K07605 | KRT4 | chr12 | 52806543 | 52814116 | 6.658683048 | 7.186331686 | 1.07E-15 | 2.7E-13 | 0.528846154 | 2.655769231 | Up |
| 238 | ENSG00000171094 | K05119 | ALK | chr2 | 29192774 | 29921611 | 5.494635627 | 6.030937994 | 0.000556252 | 0.006410041 | 0.432716049 | 2.799382716 | Up |
| 392465 | ENSG00000171433 | - | GLOD5 | chrX | 48761572 | 48773648 | 1.590014169 | 8.074423544 | 0.000253622 | 0.003259292 | 0.44375 | 3.09375 | Up |
| 253152 | ENSG00000172031 | - | EPHX4 | chr1 | 92029976 | 92063536 | 6.415939477 | 6.94540954 | 0.0000472 | 0.00069757 | 0.400552486 | 3.378453039 | Up |
| 926 | ENSG00000172116 | K06459 | CD8B | chr2 | 86815337 | 86861924 | 5.658157963 | 6.19284195 | 0.0000362 | 0.000546126 | 0.378600823 | 3.008230453 | Up |
| 220136 | ENSG00000172361 | - | CFAP53 | chr18 | 50227193 | 50266522 | 6.075644557 | 6.607046607 | 0.0000181 | 0.000290658 | 0.608949416 | 3.167315175 | Up |
| 150353 | ENSG00000172404 | K09513 | DNAJB7 | chr22 | 40859549 | 40862126 | 5.808974808 | 6.342290851 | 0.00000689 | 0.000116426 | 0.637540453 | 3.061488673 | Up |
| 408263 | ENSG00000172568 | - | FNDC9 | chr5 | 157341599 | 157345721 | 1.847547897 | 7.320918262 | 0.000263512 | 0.003353413 | 0.450892857 | 3.035714286 | Up |
| 91683 | ENSG00000173227 | K19912 | SYT12 | chr11 | 67006778 | 67050863 | 6.658683326 | 7.186331686 | 1.05E-15 | 2.7E-13 | 0.496437055 | 2.914489311 | Up |
| 122651 | ENSG00000173464 | K16633 | RNASE11 | chr14 | 20582892 | 20590258 | 6.334928218 | 6.864374614 | 2.03E-09 | 5.73E-08 | 0.537688442 | 2.859296482 | Up |
| 1464 | ENSG00000173546 | K08115 | CSPG4 | chr15 | 75674322 | 75712848 | 1.516810474 | 8.005212138 | 0.000475389 | 0.005737696 | 0.453057709 | 2.787252369 | Up |
| 10803 | ENSG00000173585 | K04184 | CCR9 | chr3 | 45886504 | 45903177 | 7.192922786 | 7.717724028 | 6.87E-23 | 7.88E-20 | 0.398373984 | 3.227642276 | Up |
| 9416 | ENSG00000174243 | K12858 | DDX23 | chr12 | 48829756 | 48852174 | 1.487617165 | 7.977683044 | 0.000444681 | 0.005414853 | 0.545121951 | 3.029268293 | Up |
| 9975 | ENSG00000174738 | K08531 | NR1D2 | chr3 | 23945260 | 23980618 | 6.399959459 | 6.929028159 | 3.07E-10 | 8.75E-09 | 0.506044905 | 2.792746114 | Up |
| 80851 | ENSG00000175137 | - | SH3BP5L | chr1 | 248810446 | 248826285 | 1.329114152 | 7.82873103 | 0.000766963 | 0.008715553 | 0.562340967 | 2.671755725 | Up |
| 22843 | ENSG00000175175 | K17501 | PPM1E | chr17 | 58755869 | 58985617 | 6.571860273 | 7.099721885 | 8.01E-14 | 4.11E-12 | 0.541721854 | 2.860927152 | Up |
| 131408 | ENSG00000175182 | - | FAM131A | chr3 | 184335924 | 184346275 | 1.487617193 | 7.977683044 | 0.000444862 | 0.005414853 | 0.543715847 | 2.636612022 | Up |
| 11007 | ENSG00000175602 | K16758 | CCDC85B | chr11 | 65890404 | 65891635 | 1.590014236 | 8.074423544 | 0.00025385 | 0.003259292 | 0.495049505 | 2.603960396 | Up |
| 3.40E+05 | ENSG00000175946 | K13959 | KLHL38 | chr8 | 123644442 | 123653829 | 7.040779632 | 7.57E+00 | 1.22E-30 | 7.01E-27 | 0.452667814 | 2.989672978 | Up |
| 79898 | ENSG00000176024 | K09228 | ZNF613 | chr19 | 51927147 | 51946147 | 2.083949912 | 7.547074131 | 0.000039 | 0.000580987 | 0.471636953 | 2.901134522 | Up |
| 9699 | ENSG00000176406 | K15297 | RIMS2 | chr8 | 103500026 | 104254430 | 6.875348785 | 7.400619776 | 5.49E-26 | 9.8E-23 | 0.577176298 | 2.859546452 | Up |
| 79324 | ENSG00000176879 | K04257 | OR51G1 | chr11 | 4923374 | 4924339 | 6.571860991 | 7.099721885 | 7.83E-14 | 4.07E-12 | 0.302180685 | 3.264797508 | Up |
| 84942 | ENSG00000177082 | - | WDR73 | chr15 | 84640682 | 84654343 | 1.451792967 | 7.943939812 | 0.000412069 | 0.005074961 | 0.468253968 | 2.875661376 | Up |
| 219770 | ENSG00000177291 | K07630 | GJD4 | chr10 | 35605410 | 35608935 | 6.658683514 | 7.186331686 | 1.03E-15 | 2.7E-13 | 0.367567568 | 2.775675676 | Up |
| 222183 | ENSG00000177679 | - | SRRM3 | chr7 | 76201893 | 76287292 | 6.660923736 | 7.188048855 | 3.64E-16 | 1.07E-13 | 0.450229709 | 2.517611026 | Up |
| 285525 | ENSG00000177752 | - | YIPF7 | chr4 | 44619578 | 44656868 | 6.040405016 | 6.572241501 | 0.000320962 | 0.004026418 | 0.442857143 | 2.95 | Up |
| 1370 | ENSG00000178772 | K13023 | CPN2 | chr3 | 194339765 | 194351387 | 5.968214428 | 6.500591737 | 0.000402281 | 0.004961034 | 0.442201835 | 3.005504587 | Up |
| 727866 | ENSG00000179304 | - | FAM156B | chrX | 52891599 | 52908560 | 5.728305981 | 6.262459201 | 0.000190174 | 0.002512137 | 0.502347418 | 2.915492958 | Up |
| 1104 | ENSG00000180198 | K11493 | RCC1 | chr1 | 28505943 | 28539196 | 1.53975137 | 8.026863556 | 0.000514676 | 0.005942872 | 0.433628319 | 2.615044248 | Up |
| 119467 | ENSG00000180745 | - | CLRN3 | chr10 | 127877841 | 127892947 | 6.402549295 | 6.931899293 | 0.00000169 | 0.000034 | 0.446902655 | 3.181415929 | Up |
| 163183 | ENSG00000181392 | - | SYNE4 | chr19 | 36003299 | 36008793 | 1.273272741 | 7.776570932 | 0.000811071 | 0.009186899 | 0.425742574 | 2.725247525 | Up |
| 1366 | ENSG00000181885 | K06087 | CLDN7 | chr17 | 7259903 | 7263193 | 2.496186501 | 7.945254811 | 0.00000336 | 0.0000579 | 0.317535545 | 2.748815166 | Up |
| 3838 | ENSG00000182481 | K15043 | KPNA2 | chr17 | 68035602 | 68046859 | 1.49702034 | 7.542152329 | 0.0000891 | 0.001247957 | 0.495274102 | 2.827977316 | Up |
| 116729 | ENSG00000182676 | K17566 | PPP1R27 | chr17 | 81833492 | 81835050 | 6.44830578 | 6.977077733 | 3.42E-11 | 9.89E-10 | 0.487012987 | 2.954545455 | Up |
| 51118 | ENSG00000183520 | K14769 | UTP11 | chr1 | 38012712 | 38024825 | 1.569255899 | 8.054796961 | 0.000232624 | 0.003041358 | 0.486166008 | 3.351778656 | Up |
| 11187 | ENSG00000184363 | - | PKP3 | chr11 | 392599 | 404908 | 6.72983027 | 7.256825487 | 2.97E-19 | 2.27E-16 | 0.460476788 | 2.747804266 | Up |
| 3751 | ENSG00000184408 | K04892 | KCND2 | chr7 | 120273635 | 120750333 | 6.26337181 | 6.793537687 | 0.00000501 | 0.0000852 | 0.461904762 | 2.977777778 | Up |
| 349334 | ENSG00000184659 | K09397 | FOXD4L4 | chr9 | 65737146 | 65738396 | 6.260493483 | 6.790377599 | 6.49E-09 | 0.000000168 | 0.355769231 | 2.838942308 | Up |
| 2865 | ENSG00000185897 | K04326 | FFAR3 | chr19 | 35358106 | 35360491 | 2.451451601 | 7.901974193 | 3.88E-08 | 0.000000988 | 0.36416185 | 3.11849711 | Up |
| 342035 | ENSG00000186417 | K16364 | GLDN | chr15 | 51341516 | 51413365 | 6.130377543 | 6.661629243 | 0.000208533 | 0.002745169 | 0.431941924 | 2.686025408 | Up |
| 51616 | ENSG00000187325 | K03133 | TAF9B | chrX | 78129748 | 78139682 | 2.022341904 | 7.488250101 | 0.0000171 | 0.00027483 | 0.458167331 | 2.844621514 | Up |
| 341947 | ENSG00000187581 | K02273 | COX8C | chr14 | 93347191 | 93348354 | 6.429673427 | 6.959095097 | 0.0000471 | 0.00069757 | 0.194444444 | 3.125 | Up |
| 51393 | ENSG00000187688 | K04971 | TRPV2 | chr17 | 16415542 | 16437003 | 6.864274347 | 7.391197769 | 2.56E-09 | 7.16E-08 | 0.45026178 | 3.086387435 | Up |
| 643161 | ENSG00000188100 | - | FAM25A | chr10 | 87020289 | 87024730 | 6.654207956 | 7.181615958 | 4.41E-16 | 1.23E-13 | 0.505617978 | 2.494382022 | Up |
| 26155 | ENSG00000188976 | K14833 | NOC2L | chr1 | 944203 | 959299 | 5.882236546 | 6.415289425 | 0.000532685 | 0.006144635 | 0.52870494 | 3.001335113 | Up |
| 100133093 | ENSG00000189090 | - | FAM25G | chr10 | 47487236 | 47491700 | 6.654208469 | 7.181615958 | 4.32E-16 | 1.23E-13 | 0.505617978 | 2.494382022 | Up |
| 170062 | ENSG00000189132 | - | FAM47B | chrX | 34942796 | 34944917 | 7.491823797 | 8.015836978 | 1.86E-28 | 5.32E-25 | 0.491472868 | 3.048062016 | Up |
| 54467 | ENSG00000001629 | K11967 | ANKIB1 | chr7 | 92246216 | 92401773 | -6.128887369 | 4.964945407 | 5.66E-17 | 1.92E-14 | 0.561983471 | 2.838383838 | Down |
| 3382 | ENSG00000003147 | K19863 | ICA1 | chr7 | 8113184 | 8262687 | -2.961929329 | 3.698629645 | 0.000491483 | 0.00591586 | 0.552734375 | 2.951171875 | Down |
| 23028 | ENSG00000004487 | K11450 | KDM1A | chr1 | 23019443 | 23083691 | -3.33846241 | 4.089072688 | 0.00013543 | 0.001828784 | 0.470319635 | 2.799086758 | Down |
| 64063 | ENSG00000005001 | K09626 | PRSS22 |  |  |  | -4.136903232 | 3.326189908 | 1.07E-12 | 4.46E-11 | 0.356466877 | 2.690851735 | Down |
| 55013 | ENSG00000005059 | - | CCDC109B | chr4 | 109560199 | 109688719 | -4.688411511 | 3.739046202 | 4.82E-14 | 3.1E-12 | 0.44047619 | 3.273809524 | Down |
| 25998 | ENSG00000005700 | - | IBTK |  |  |  | -5.131963798 | 4.096541259 | 5.28E-15 | 8.93E-13 | 0.486326681 | 2.924611973 | Down |
| 55049 | ENSG00000006015 | - | C19orf60 | chr19 | 18588685 | 18592337 | -3.095888921 | 2.674084864 | 0.00000183 | 0.0000366 | 0.457711443 | 2.900497512 | Down |
| 114881 | ENSG00000006025 | K20463 | OSBPL7 |  |  |  | -5.131959137 | 4.096541259 | 5.33E-15 | 8.93E-13 | 0.503562945 | 2.923990499 | Down |
| 54972 | ENSG00000006118 | K17599 | TMEM132A |  |  |  | -4.136903284 | 3.326189908 | 1.07E-12 | 4.46E-11 | 0.426757813 | 2.748046875 | Down |
| 64847 | ENSG00000006282 | - | SPATA20 | chr17 | 50547089 | 50555852 | -4.688406278 | 3.739046202 | 4.86E-14 | 3.11E-12 | 0.457605985 | 2.956359102 | Down |
| 5080 | ENSG00000007372 | K08031 | PAX6 | chr11 | 31784792 | 31817961 | -3.262616678 | 2.765916931 | 0.000000939 | 0.0000217 | 0.513761468 | 2.747706422 | Down |
| 1870 | ENSG00000007968 | K09389 | E2F2 | chr1 | 23505696 | 23531266 | -4.136903336 | 3.326189908 | 1.07E-12 | 4.46E-11 | 0.514874142 | 2.819221968 | Down |
| 1534 | ENSG00000008283 | K08360 | CYB561 | chr17 | 63432304 | 63446361 | -3.179033749 | 4.148862116 | 0.000342896 | 0.004281642 | 0.323899371 | 3.062893082 | Down |
| 10157 | ENSG00000008311 | K14157 | AASS | chr7 | 122073544 | 122144290 | -5.131953934 | 4.096541259 | 5.39E-15 | 8.93E-13 | 0.450323974 | 2.914686825 | Down |
| 55200 | ENSG00000008323 | - | PLEKHG6 |  |  |  | -4.747950039 | 3.785577849 | 7.75E-14 | 4.06E-12 | 0.486075949 | 2.941772152 | Down |
| 23221 | ENSG00000008853 | K07868 | RHOBTB2 |  |  |  | -3.262612664 | 2.765916931 | 0.000000942 | 0.0000218 | 0.465954606 | 3.029372497 | Down |
| 29954 | ENSG00000009830 | K00728 | POMT2 | chr14 | 77274956 | 77320885 | -3.784375718 | 4.376575437 | 0.0000216 | 0.000343343 | 0.346666667 | 3.165333333 | Down |
| 9902 | ENSG00000011028 | K06560 | MRC2 | chr17 | 62627401 | 62693601 | -4.136903389 | 3.326189908 | 1.07E-12 | 4.46E-11 | 0.497633536 | 2.943204868 | Down |
| 51099 | ENSG00000011198 | K13699 | ABHD5 |  |  |  | -4.688401049 | 3.739046202 | 4.9E-14 | 3.12E-12 | 0.444126074 | 3.025787966 | Down |
| 8491 | ENSG00000011566 | K04406 | MAP4K3 | chr2 | 39249266 | 39437312 | -4.136903441 | 3.326189908 | 1.06E-12 | 4.46E-11 | 0.475391499 | 3.032438479 | Down |
| 57146 | ENSG00000011638 | - | TMEM159 | chr16 | 21158349 | 21180616 | -3.784377822 | 4.376575437 | 0.0000216 | 0.000343343 | 0.394594595 | 2.913513514 | Down |
| 4057 | ENSG00000012223 | K17283 | LTF |  |  |  | -3.262608651 | 2.765916931 | 0.000000945 | 0.0000218 | 0.452112676 | 2.828169014 | Down |
| 23357 | ENSG00000013523 | K18729 | ANGEL1 |  |  |  | -4.136903493 | 3.326189908 | 1.06E-12 | 4.46E-11 | 0.491044776 | 2.974626866 | Down |
| 1774 | ENSG00000013563 | K11995 | DNASE1L1 | chrX | 154401236 | 154412101 | -4.74794375 | 3.785577849 | 7.84E-14 | 4.07E-12 | 0.486754967 | 3.046357616 | Down |
| 10046 | ENSG00000013619 | K19512 | MAMLD1 | chrX | 150361422 | 150514178 | -3.517005432 | 4.026762593 | 0.0000471 | 0.00069757 | 0.46492986 | 2.649298597 | Down |
| 4190 | ENSG00000014641 | K00025 | MDH1 | chr2 | 63588609 | 63607197 | -5.047541394 | 4.031698835 | 9.72E-11 | 2.79E-09 | 0.460227273 | 2.894886364 | Down |
| 10903 | ENSG00000014914 | K18085 | MTMR11 | chr1 | 149928651 | 149936898 | -3.963183149 | 4.325612745 | 0.00000699 | 0.000117822 | 0.441466855 | 2.990126939 | Down |
| 22902 | ENSG00000018189 | - | RUFY3 | chr4 | 70704204 | 70808622 | -5.13194948 | 4.096541259 | 5.44E-15 | 8.93E-13 | 0.567741935 | 2.914516129 | Down |
| 477 | ENSG00000018625 | K01539 | ATP1A2 | chr1 | 160115730 | 160143591 | -5.13194382 | 4.096541259 | 5.5E-15 | 8.93E-13 | 0.460784314 | 2.881372549 | Down |
| 57586 | ENSG00000019505 | K19913 | SYT13 |  |  |  | -3.095884804 | 2.674084864 | 0.00000184 | 0.0000367 | 0.457746479 | 2.830985915 | Down |
| 4719 | ENSG00000023228 | K03934 | NDUFS1 | chr2 | 206121971 | 206159519 | -3.963184801 | 4.325612745 | 0.000007 | 0.000117822 | 0.457489879 | 2.777327935 | Down |
| 50619 | ENSG00000023892 | - | DEF6 | chr6 | 35297818 | 35321771 | -4.136903545 | 3.326189908 | 1.06E-12 | 4.46E-11 | 0.589540412 | 3.047543582 | Down |
| 6642 | ENSG00000028528 | K17917 | SNX1 | chr15 | 64094071 | 64144234 | -4.064745818 | 3.280835385 | 5.53E-09 | 0.000000153 | 0.560143627 | 3.017953321 | Down |
| 28990 | ENSG00000034533 | - | ASTE1 | chr3 | 131013873 | 131026854 | -5.683914305 | 4.568749497 | 1.84E-15 | 3.58E-13 | 0.455965909 | 3.059659091 | Down |
| 6570 | ENSG00000036565 | K08155 | SLC18A1 |  |  |  | -4.136903597 | 3.326189908 | 1.06E-12 | 4.46E-11 | 0.344761905 | 2.893333333 | Down |
| 54455 | ENSG00000037637 | K10317 | FBXO42 | chr1 | 16246840 | 16352491 | -3.480509545 | 4.47349386 | 0.000100564 | 0.00139765 | 0.447698745 | 2.760111576 | Down |
| 79567 | ENSG00000039523 | - | FAM65A |  |  |  | -4.68839508 | 3.739046202 | 4.95E-14 | 3.14E-12 | 0.497982244 | 2.714285714 | Down |
| 26064 | ENSG00000039560 | - | RAI14 | chr5 | 34656328 | 34832612 | -5.131938166 | 4.096541259 | 5.57E-15 | 8.93E-13 | 0.611393693 | 2.84231943 | Down |
| 23035 | ENSG00000040199 | K16340 | PHLPP2 | chr16 | 71644924 | 71724701 | -3.095880691 | 2.674084864 | 0.00000184 | 0.0000367 | 0.516200295 | 2.834315169 | Down |
| 84883 | ENSG00000042286 | - | AIFM2 |  |  |  | -4.747937466 | 3.785577849 | 7.93E-14 | 4.1E-12 | 0.423592493 | 2.833780161 | Down |
| 56918 | ENSG00000042304 | - | C2orf83 |  |  |  | -3.095876289 | 2.674084864 | 0.00000185 | 0.0000368 | 0.446666667 | 2.813333333 | Down |
| 54802 | ENSG00000043514 | K00791 | TRIT1 |  |  |  | -5.131932515 | 4.096541259 | 5.63E-15 | 8.93E-13 | 0.488222698 | 2.976445396 | Down |
| 22884 | ENSG00000047056 | - | WDR37 | chr10 | 1056836 | 1132297 | -3.603617881 | 4.976015926 | 0.00021624 | 0.002835563 | 0.528340081 | 2.767206478 | Down |
| 51606 | ENSG00000047249 | K02144 | ATP6V1H | chr8 | 53715543 | 53843311 | -4.179256469 | 4.813376398 | 0.00000441 | 0.0000752 | 0.519668737 | 3.109730849 | Down |
| 4052 | ENSG00000049323 | K19559 | LTBP1 | chr2 | 32947129 | 33399509 | -4.136903649 | 3.326189908 | 1.06E-12 | 4.46E-11 | 0.489250436 | 2.619988379 | Down |
| 9603 | ENSG00000050344 | K09040 | NFE2L3 | chr7 | 26152227 | 26187137 | -4.238383217 | 3.40286445 | 5.5E-09 | 0.000000152 | 0.553314121 | 2.749279539 | Down |
| 55733 | ENSG00000054392 | - | HHAT | chr1 | 210327873 | 210676298 | -3.745210802 | 4.990898398 | 0.000248551 | 0.003209328 | 0.368421053 | 3.307692308 | Down |
| 6646 | ENSG00000057252 | K00637 | SOAT1 | chr1 | 179293714 | 179358680 | -3.399126043 | 4.125804243 | 0.000125448 | 0.001712361 | 0.392727273 | 3.534545455 | Down |
| 54432 | ENSG00000058799 | - | YIPF1 | chr1 | 53851719 | 53889831 | -3.262604641 | 2.765916931 | 0.000000948 | 0.0000218 | 0.437908497 | 3.156862745 | Down |
| 55359 | ENSG00000060140 | K17510 | STYK1 |  |  |  | -5.131926869 | 4.096541259 | 5.7E-15 | 8.93E-13 | 0.41943128 | 3.068720379 | Down |
| 10188 | ENSG00000061938 | K08886 | TNK2 | chr3 | 195863364 | 195909009 | -3.663004504 | 5.396202697 | 0.000127647 | 0.001730028 | 0.423572744 | 2.863720074 | Down |
| 22998 | ENSG00000064042 | - | LIMCH1 | chr4 | 41359607 | 41700044 | -4.136903701 | 3.326189908 | 1.06E-12 | 4.46E-11 | 0.591002045 | 2.783912747 | Down |
| 8839 | ENSG00000064205 | - | WISP2 | chr20 | 44714204 | 44728041 | -4.136903753 | 3.326189908 | 1.06E-12 | 4.46E-11 | 0.344 | 2.552 | Down |
| 1265 | ENSG00000064666 | - | CNN2 | chr19 | 1026275 | 1039068 | -3.095871891 | 2.674084864 | 0.00000185 | 0.0000368 | 0.496969697 | 2.727272727 | Down |
| 23536 | ENSG00000065457 | K15440 | ADAT1 | chr16 | 75596879 | 75623323 | -4.136903805 | 3.326189908 | 1.06E-12 | 4.46E-11 | 0.432270916 | 2.848605578 | Down |
| 51665 | ENSG00000065802 | K10323 | ASB1 |  |  |  | -3.963186453 | 4.325612745 | 0.00000701 | 0.000117822 | 0.388059701 | 2.865671642 | Down |
| 8621 | ENSG00000065883 | K08819 | CDK13 | chr7 | 39949501 | 40097134 | -3.480510338 | 4.47349386 | 0.000100694 | 0.001397765 | 0.492063492 | 2.721560847 | Down |
| 6688 | ENSG00000066336 | K09438 | SPI1 |  |  |  | -4.136903857 | 3.326189908 | 1.06E-12 | 4.46E-11 | 0.520295203 | 3.118081181 | Down |
| 30836 | ENSG00000067334 | - | DNTTIP2 | chr1 | 93869458 | 93879206 | -5.131921228 | 4.096541259 | 5.76E-15 | 8.93E-13 | 0.654761905 | 2.753968254 | Down |
| 10235 | ENSG00000068831 | K12361 | RASGRP2 | chr11 | 64726911 | 64745456 | -2.961932782 | 3.698629645 | 0.000492153 | 0.00591586 | 0.463934426 | 3.03442623 | Down |
| 602 | ENSG00000069399 | K09258 | BCL3 | chr19 | 44742621 | 44760044 | -3.229785636 | 4.563637906 | 0.00089293 | 0.009774872 | 0.365638767 | 2.59030837 | Down |
| 7049 | ENSG00000069702 | K05843 | TGFBR3 | chr1 | 91680343 | 91906002 | -3.095868716 | 2.674084864 | 0.00000186 | 0.0000368 | 0.479435958 | 2.890716804 | Down |
| 10681 | ENSG00000069966 | K04539 | GNB5 | chr15 | 52120926 | 52191368 | -4.688389116 | 3.739046202 | 5.01E-14 | 3.15E-12 | 0.493670886 | 2.77721519 | Down |
| 27071 | ENSG00000070190 | K12229 | DAPP1 | chr4 | 99816824 | 99872290 | -4.747931187 | 3.785577849 | 8.02E-14 | 4.11E-12 | 0.510714286 | 3.142857143 | Down |
| 3340 | ENSG00000070614 | K02576 | NDST1 | chr5 | 150485783 | 150558211 | -3.78437974 | 4.376575437 | 0.0000217 | 0.000343343 | 0.43877551 | 3.227891156 | Down |
| 815 | ENSG00000070808 | K04515 | CAMK2A | chr5 | 150219491 | 150289840 | -4.130257893 | 5.263652978 | 0.0000504 | 0.000733591 | 0.474437628 | 3.024539877 | Down |
| 9448 | ENSG00000071054 | K04407 | MAP4K4 |  |  |  | -5.131915591 | 4.096541259 | 5.83E-15 | 8.93E-13 | 0.556060606 | 2.965909091 | Down |
| 54556 | ENSG00000071243 | K11319 | ING3 |  |  |  | -3.262600634 | 2.765916931 | 0.000000951 | 0.0000219 | 0.61722488 | 2.73923445 | Down |
| 23503 | ENSG00000072121 | K19027 | ZFYVE26 | chr14 | 67728892 | 67817293 | -4.238378875 | 3.40286445 | 5.53E-09 | 0.000000153 | 0.490744388 | 2.933044506 | Down |
| 100996693 | ENSG00000072195 | - | LOC100996693 | chr2 | 219493737 | 219498287 | -3.095865545 | 2.674084864 | 0.00000186 | 0.0000368 | 0.452708907 | 2.723905724 | Down |
| 224 | ENSG00000072210 | K00128 | ALDH3A2 | chr17 | 19648136 | 19677596 | -3.624670387 | 4.425833018 | 0.0000521 | 0.000757196 | 0.427165354 | 3.147637795 | Down |
| 2213 | ENSG00000072694 | K12560 | FCGR2B |  |  |  | -3.262596628 | 2.765916931 | 0.000000954 | 0.0000219 | 0.474193548 | 2.887096774 | Down |
| 6793 | ENSG00000072786 | K08837 | STK10 | chr5 | 172042070 | 172188342 | -3.095862376 | 2.674084864 | 0.00000187 | 0.0000368 | 0.579545455 | 3.007231405 | Down |
| 23291 | ENSG00000072803 | K03362 | FBXW11 | chr5 | 171861549 | 172006873 | -3.577565718 | 4.065055456 | 0.0000383 | 0.000574947 | 0.507380074 | 3.001845018 | Down |
| 54905 | ENSG00000073067 | K07423 | CYP2W1 | chr7 | 983157 | 989640 | -6.128886143 | 4.964945407 | 5.68E-17 | 1.92E-14 | 0.33877551 | 2.971428571 | Down |
| 54825 | ENSG00000074276 | K16502 | CDHR2 | chr5 | 176542511 | 176595768 | -5.131909959 | 4.096541259 | 5.9E-15 | 8.93E-13 | 0.535114504 | 2.780916031 | Down |
| 10512 | ENSG00000075223 | K06840 | SEMA3C | chr7 | 80742538 | 80919351 | -3.624671408 | 4.425833018 | 0.0000521 | 0.000757196 | 0.482023968 | 2.994673768 | Down |
| 23016 | ENSG00000075914 | K12589 | EXOSC7 | chr3 | 44975291 | 45012668 | -3.963188104 | 4.325612745 | 0.00000702 | 0.000117822 | 0.505154639 | 2.776632302 | Down |
| 88455 | ENSG00000076513 | K21437 | ANKRD13A | chr12 | 109999169 | 110039432 | -4.747924914 | 3.785577849 | 8.11E-14 | 4.13E-12 | 0.579661017 | 3.038983051 | Down |
| 22978 | ENSG00000076685 | K01081 | NT5C2 | chr10 | 103088017 | 103193306 | -4.064739868 | 3.280835385 | 5.57E-09 | 0.000000153 | 0.536541889 | 3.212121212 | Down |
| 8527 | ENSG00000077044 | K00901 | DGKD | chr2 | 233354507 | 233472098 | -3.784380237 | 4.376575437 | 0.0000217 | 0.000343343 | 0.485996705 | 2.825370675 | Down |
| 1641 | ENSG00000077279 | K16579 | DCX |  |  |  | -4.136903909 | 3.326189908 | 1.06E-12 | 4.46E-11 | 0.52154195 | 2.863945578 | Down |
| 4034 | ENSG00000077454 | - | LRCH4 | chr7 | 100574011 | 100586188 | -3.0478294 | 4.636091453 | 0.000750738 | 0.008545699 | 0.439238653 | 2.691068814 | Down |
| 203427 | ENSG00000077713 | K15120 | SLC25A43 |  |  |  | -3.229786858 | 4.563637906 | 0.000893619 | 0.009774872 | 0.337243402 | 3.038123167 | Down |
| 1906 | ENSG00000078401 | K16366 | EDN1 | chr6 | 12256464 | 12297194 | -2.961935839 | 3.698629645 | 0.000492746 | 0.00591586 | 0.448113208 | 2.971698113 | Down |
| 117 | ENSG00000078549 | K04587 | ADCYAP1R1 | chr7 | 31052341 | 31114005 | -4.136903962 | 3.326189908 | 1.06E-12 | 4.46E-11 | 0.437022901 | 3.158396947 | Down |
| 57690 | ENSG00000078687 | K18412 | TNRC6C |  |  |  | -3.624672429 | 4.425833018 | 0.0000522 | 0.000757196 | 0.496523754 | 2.418887601 | Down |
| 10590 | ENSG00000079689 | - | SCGN |  |  |  | -5.13190433 | 4.096541259 | 5.97E-15 | 8.93E-13 | 0.536231884 | 3.148550725 | Down |
| 11158 | ENSG00000079974 | K07931 | RABL2B | chr22 | 50767492 | 50783705 | -4.136904014 | 3.326189908 | 1.06E-12 | 4.46E-11 | 0.596638655 | 3.071428571 | Down |
| 8671 | ENSG00000080493 | K13575 | SLC4A4 |  |  |  | -4.747918646 | 3.785577849 | 8.2E-14 | 4.13E-12 | 0.451553931 | 3.10786106 | Down |
| 1136 | ENSG00000080644 | K04805 | CHRNA3 | chr15 | 78593052 | 78621295 | -3.338464049 | 4.089072688 | 0.000135594 | 0.001828784 | 0.447524752 | 3.203960396 | Down |
| 6604 | ENSG00000082014 | K11650 | SMARCD3 | chr7 | 151238973 | 151277436 | -5.74412613 | 4.621282224 | 1.57E-15 | 3.55E-13 | 0.48447205 | 3.022774327 | Down |
| 1741 | ENSG00000082458 | K21098 | DLG3 | chrX | 70444850 | 70505490 | -3.09585921 | 2.674084864 | 0.00000187 | 0.0000368 | 0.487632509 | 2.842167256 | Down |
| 7188 | ENSG00000082512 | K09849 | TRAF5 | chr1 | 211326587 | 211374946 | -2.961939877 | 3.698629645 | 0.000493532 | 0.00591586 | 0.508078995 | 3.080789946 | Down |
| 26747 | ENSG00000083635 | - | NUFIP1 | chr13 | 44939249 | 44989478 | -4.747910852 | 3.785577849 | 8.32E-14 | 4.15E-12 | 0.507070707 | 3.018181818 | Down |
| 5019 | ENSG00000083720 | K01027 | OXCT1 | chr5 | 41730065 | 41870689 | -4.964029802 | 5.094673907 | 5.43E-08 | 0.00000136 | 0.430769231 | 2.780769231 | Down |
| 64744 | ENSG00000084070 | K12486 | SMAP2 | chr1 | 40373706 | 40423326 | -5.131898707 | 4.096541259 | 6.04E-15 | 8.93E-13 | 0.440559441 | 2.734265734 | Down |
| 54434 | ENSG00000084112 | K05766 | SSH1 | chr12 | 108782690 | 108857606 | -2.961942762 | 3.698629645 | 0.000494094 | 0.00591586 | 0.523355577 | 2.795996187 | Down |
| 338 | ENSG00000084674 | K14462 | APOB |  |  |  | -3.095856047 | 2.674084864 | 0.00000187 | 0.0000368 | 0.522024984 | 3.075608153 | Down |
| 2646 | ENSG00000084734 | - | GCKR |  |  |  | -4.136896245 | 3.326189908 | 1.08E-12 | 4.48E-11 | 0.448 | 2.8992 | Down |
| 55624 | ENSG00000085998 | K09666 | POMGNT1 | chr1 | 46188681 | 46220305 | -5.131893088 | 4.096541259 | 6.11E-15 | 8.93E-13 | 0.461229947 | 3.053475936 | Down |
| 55616 | ENSG00000088280 | K12488 | ASAP3 | chr1 | 23428563 | 23484631 | -2.961945272 | 3.698629645 | 0.000494583 | 0.00591586 | 0.50055371 | 2.774086379 | Down |
| 23483 | ENSG00000088451 | K01710 | TGDS | chr13 | 94574054 | 94596275 | -3.480511131 | 4.47349386 | 0.000100824 | 0.001397881 | 0.494285714 | 3.222857143 | Down |
| 25943 | ENSG00000088854 | - | C20orf194 |  |  |  | -3.095852888 | 2.674084864 | 0.00000188 | 0.0000369 | 0.491928632 | 3.020390824 | Down |
| 113878 | ENSG00000091073 | K06058 | DTX2 | chr7 | 76461655 | 76505995 | -3.480511923 | 4.47349386 | 0.000100954 | 0.001397998 | 0.427652733 | 2.745980707 | Down |
| 1811 | ENSG00000091138 | K14078 | SLC26A3 | chr7 | 107765467 | 107803233 | -5.131889431 | 4.096541259 | 6.16E-15 | 8.93E-13 | 0.393979058 | 3.095549738 | Down |
| 6913 | ENSG00000092607 | K10182 | TBX15 |  |  |  | -4.688383157 | 3.739046202 | 5.06E-14 | 3.17E-12 | 0.503322259 | 2.740863787 | Down |
| 10044 | ENSG00000095370 | - | SH2D3C |  |  |  | -4.136888483 | 3.326189908 | 1.09E-12 | 4.51E-11 | 0.477906977 | 2.815116279 | Down |
| 284422 | ENSG00000095932 | - | SMIM24 | chr19 | 3474407 | 3480542 | -3.784381182 | 4.376575437 | 0.0000217 | 0.000343343 | 0.576923077 | 3.076923077 | Down |
| 80270 | ENSG00000099377 | K12408 | HSD3B7 | chr16 | 30985189 | 30989152 | -3.844794297 | 4.961085769 | 0.0000866 | 0.001219919 | 0.376693767 | 3.013550136 | Down |
| 3483 | ENSG00000099769 | K17256 | IGFALS |  |  |  | -5.131885776 | 4.096541259 | 6.2E-15 | 8.93E-13 | 0.388802488 | 2.877138414 | Down |
| 5064 | ENSG00000099864 | K16519 | PALM | chr19 | 708953 | 748330 | -4.136880729 | 3.326189908 | 1.1E-12 | 4.55E-11 | 0.589147287 | 2.607235142 | Down |
| 29801 | ENSG00000099904 | K20030 | ZDHHC8 | chr22 | 20131841 | 20148007 | -5.131882123 | 4.096541259 | 6.25E-15 | 8.93E-13 | 0.366323907 | 2.748071979 | Down |
| 8216 | ENSG00000099949 | - | LZTR1 | chr22 | 20982269 | 20999037 | -4.747905764 | 3.785577849 | 8.4E-14 | 4.17E-12 | 0.467857143 | 3.023809524 | Down |
| 9127 | ENSG00000099957 | K05221 | P2RX6 | chr22 | 21009699 | 21028013 | -4.136872386 | 3.326189908 | 1.11E-12 | 4.58E-11 | 0.41723356 | 2.986394558 | Down |
| 102724197 | ENSG00000100031 | - | LOC102724197 | chr22 | 65299 | 86956 | -6.12888529 | 4.964945407 | 5.69E-17 | 1.92E-14 | 0.427065026 | 2.804920914 | Down |
| 9681 | ENSG00000100150 | K20404 | DEPDC5 |  |  |  | -4.688375914 | 3.739046202 | 5.12E-14 | 3.19E-12 | 0.512164691 | 2.998128509 | Down |
| 79019 | ENSG00000100162 | K11505 | CENPM | chr22 | 41927747 | 41947164 | -3.095849731 | 2.674084864 | 0.00000188 | 0.0000369 | 0.422222222 | 2.861111111 | Down |
| 11015 | ENSG00000100196 | K10949 | KDELR3 |  |  |  | -2.961948787 | 3.698629645 | 0.00049527 | 0.00591586 | 0.440909091 | 3.486363636 | Down |
| 84271 | ENSG00000100227 | - | POLDIP3 | chr22 | 42583721 | 42614962 | -4.578849758 | 4.784107766 | 0.000000468 | 0.0000111 | 0.429223744 | 2.796803653 | Down |
| 638 | ENSG00000100290 | K18452 | BIK | chr22 | 43110748 | 43129712 | -3.624673186 | 4.425833018 | 0.0000523 | 0.000757196 | 0.51875 | 2.975 | Down |
| 150379 | ENSG00000100341 | K16814 | PNPLA5 |  |  |  | -5.131878472 | 4.096541259 | 6.3E-15 | 8.93E-13 | 0.405594406 | 3.03030303 | Down |
| 11020 | ENSG00000100360 | K07934 | IFT27 |  |  |  | -3.963191129 | 4.325612745 | 0.00000703 | 0.000117911 | 0.462365591 | 2.908602151 | Down |
| 10478 | ENSG00000100372 | K13354 | SLC25A17 | chr22 | 40769630 | 40819399 | -4.688368678 | 3.739046202 | 5.19E-14 | 3.21E-12 | 0.348534202 | 3.175895765 | Down |
| 51382 | ENSG00000100554 | K02149 | ATP6V1D | chr14 | 67337864 | 67360003 | -4.13685946 | 3.326189908 | 1.13E-12 | 4.65E-11 | 0.502024291 | 3.076923077 | Down |
| 3705 | ENSG00000100605 | K00913 | ITPK1 | chr14 | 92936914 | 93115918 | -5.131874823 | 4.096541259 | 6.35E-15 | 8.93E-13 | 0.475845411 | 2.855072464 | Down |
| 9252 | ENSG00000100784 | K04445 | RPS6KA5 | chr14 | 90868122 | 91060649 | -3.338465687 | 4.089072688 | 0.000135758 | 0.001828784 | 0.496902107 | 2.982651797 | Down |
| 5693 | ENSG00000100804 | K02737 | PSMB5 | chr14 | 23016543 | 23035220 | -4.136846554 | 3.326189908 | 1.15E-12 | 4.71E-11 | 0.437262357 | 2.790874525 | Down |
| 84312 | ENSG00000100916 | K19196 | BRMS1L | chr14 | 35825596 | 35871963 | -4.136839496 | 3.326189908 | 1.16E-12 | 4.74E-11 | 0.66873065 | 3.021671827 | Down |
| 4773 | ENSG00000101096 | K17332 | NFATC2 | chr20 | 51386957 | 51562857 | -4.747901242 | 3.785577849 | 8.46E-14 | 4.18E-12 | 0.462702703 | 2.761081081 | Down |
| 26164 | ENSG00000101181 | K03979 | MTG2 | chr20 | 62183025 | 62203568 | -3.338467324 | 4.089072688 | 0.000135922 | 0.001828784 | 0.36453202 | 2.810344828 | Down |
| 28231 | ENSG00000101187 | K14354 | SLCO4A1 | chr20 | 62642445 | 62690700 | -3.315800032 | 4.792956557 | 0.00073675 | 0.008397198 | 0.358725762 | 2.826869806 | Down |
| 54976 | ENSG00000101220 | - | C20orf27 | chr20 | 3753499 | 3768388 | -4.480531819 | 5.183581805 | 0.000000802 | 0.0000187 | 0.391959799 | 2.809045226 | Down |
| 79133 | ENSG00000101247 | K18162 | NDUFAF5 | chr20 | 13785026 | 13821580 | -4.688363967 | 3.739046202 | 5.23E-14 | 3.22E-12 | 0.48115942 | 3.005797101 | Down |
| 10326 | ENSG00000101307 | K06551 | SIRPB1 | chr20 | 1564193 | 1620457 | -3.229788079 | 4.563637906 | 0.000894308 | 0.009774872 | 0.459798995 | 2.788944724 | Down |
| 79913 | ENSG00000101442 | K11672 | ACTR5 | chr20 | 38748454 | 38772446 | -3.239489399 | 4.184151988 | 0.000281693 | 0.003570862 | 0.507413509 | 2.97199341 | Down |
| 60625 | ENSG00000101452 | K13117 | DHX35 | chr20 | 38962338 | 39039723 | -4.136832443 | 3.326189908 | 1.17E-12 | 4.77E-11 | 0.438122333 | 3.014224751 | Down |
| 128869 | ENSG00000101464 | K05293 | PIGU | chr20 | 34560542 | 34677285 | -5.131869425 | 4.096541259 | 6.42E-15 | 8.93E-13 | 0.337931034 | 3.55862069 | Down |
| 22850 | ENSG00000101544 | - | ADNP2 | chr18 | 80109031 | 80140346 | -4.33913256 | 4.775802366 | 0.00000178 | 0.0000358 | 0.387267905 | 2.854111406 | Down |
| 28952 | ENSG00000101997 | - | CCDC22 |  |  |  | -5.04753873 | 4.031698835 | 9.77E-11 | 2.8E-09 | 0.507177033 | 2.931419458 | Down |
| 6855 | ENSG00000102003 | - | SYP |  |  |  | -4.136825422 | 3.326189908 | 1.18E-12 | 4.8E-11 | 0.434504792 | 2.916932907 | Down |
| 6594 | ENSG00000102038 | K11727subfamily | SMARCA1 | chrX | 129446501 | 129523564 | -4.136818406 | 3.326189908 | 1.19E-12 | 4.82E-11 | 0.527102804 | 3.142056075 | Down |
| 3750 | ENSG00000102057 | K04891 | KCND1 | chrX | 48960983 | 48972099 | -4.688359258 | 3.739046202 | 5.27E-14 | 3.23E-12 | 0.432766615 | 2.887171561 | Down |
| 81617 | ENSG00000102547 | K08272 | CAB39L | chr13 | 49308628 | 49444346 | -4.136811395 | 3.326189908 | 1.21E-12 | 4.85E-11 | 0.49851632 | 3.270029674 | Down |
| 1203 | ENSG00000102805 | K12390 | CLN5 | chr13 | 76991924 | 77002517 | -5.131864839 | 4.096541259 | 6.48E-15 | 8.93E-13 | 0.417690418 | 3.194103194 | Down |
| 79650 | ENSG00000103005 | - | USB1 | chr16 | 57999600 | 58021623 | -3.262592625 | 2.765916931 | 0.000000957 | 0.0000219 | 0.486792453 | 3.090566038 | Down |
| 55697 | ENSG00000103043 | K15305 | VAC14 | chr16 | 70687439 | 70801169 | -3.229788647 | 4.563637906 | 0.000894629 | 0.009774872 | 0.482097187 | 3.031969309 | Down |
| 23406 | ENSG00000103187 | - | COTL1 | chr16 | 84565596 | 84618096 | -4.688354553 | 3.739046202 | 5.31E-14 | 3.24E-12 | 0.542253521 | 2.971830986 | Down |
| 64779 | ENSG00000103248 | - | MTHFSD |  |  |  | -3.784382127 | 4.376575437 | 0.0000217 | 0.000343343 | 0.428198433 | 2.845953003 | Down |
| 55313 | ENSG00000103381 | - | CPPED1 | chr16 | 12659799 | 12803887 | -3.784383711 | 4.376575437 | 0.0000218 | 0.000343343 | 0.48089172 | 3.066878981 | Down |
| 27324 | ENSG00000103460 | - | TOX3 | chr16 | 52436415 | 52547802 | -3.239490274 | 4.184151988 | 0.000281932 | 0.003570862 | 0.526041667 | 2.751736111 | Down |
| 23322 | ENSG00000103494 | K16550 | RPGRIP1L | chr16 | 53599239 | 53703934 | -3.784385338 | 4.376575437 | 0.0000218 | 0.000343343 | 0.599239544 | 3.02661597 | Down |
| 57020 | ENSG00000103544 | - | C16orf62 |  |  |  | -3.624674115 | 4.425833018 | 0.0000523 | 0.000757196 | 0.465779468 | 3.009505703 | Down |
| 10391 | ENSG00000103647 | K13887 | CORO2B | chr15 | 68518373 | 68727806 | -3.262588624 | 2.765916931 | 0.00000096 | 0.0000219 | 0.470833333 | 3.0875 | Down |
| 2184 | ENSG00000103876 | K01555 | FAH | chr15 | 80152891 | 80186582 | -4.13680439 | 3.326189908 | 1.22E-12 | 4.88E-11 | 0.415274463 | 2.961813842 | Down |
| 64397 | ENSG00000103994 | - | ZNF106 | chr15 | 42412437 | 42491197 | -6.128884659 | 4.964945407 | 5.7E-17 | 1.92E-14 | 0.575146044 | 2.741901221 | Down |
| 29106 | ENSG00000104112 | - | SCG3 |  |  |  | -4.136793916 | 3.326189908 | 1.24E-12 | 4.93E-11 | 0.647435897 | 2.967948718 | Down |
| 11339 | ENSG00000104147 | K11565 | OIP5 | chr15 | 41309268 | 41332621 | -3.691615033 | 5.387008694 | 0.000240562 | 0.003120225 | 0.406113537 | 2.742358079 | Down |
| 10404 | ENSG00000104324 | K01302 | CPQ |  |  |  | -4.136783456 | 3.326189908 | 1.25E-12 | 4.99E-11 | 0.455508475 | 2.906779661 | Down |
| 6788 | ENSG00000104375 | K04412 | STK3 |  |  |  | -5.131860257 | 4.096541259 | 6.54E-15 | 8.93E-13 | 0.572254335 | 3.015414258 | Down |
| 7264 | ENSG00000104522 | K02377 | TSTA3 | chr8 | 143612618 | 143618043 | -4.136773008 | 3.326189908 | 1.27E-12 | 5.04E-11 | 0.473520249 | 3.012461059 | Down |
| 23484 | ENSG00000104660 | - | LEPROTL1 |  |  |  | -3.095846578 | 2.674084864 | 0.00000189 | 0.0000369 | 0.284023669 | 3 | Down |
| 10382 | ENSG00000104833 | K07375 | TUBB4A | chr19 | 6494319 | 6502848 | -3.262584625 | 2.765916931 | 0.000000964 | 0.000022 | 0.531531532 | 2.905405405 | Down |
| 729440 | ENSG00000104983 | K16755 | CCDC61 |  |  |  | -4.747896722 | 3.785577849 | 8.53E-14 | 4.2E-12 | 0.49609375 | 2.76171875 | Down |
| 51343 | ENSG00000105325 | K03364 | FZR1 | chr19 | 3506115 | 3536757 | -4.688347799 | 3.739046202 | 5.38E-14 | 3.26E-12 | 0.485887097 | 2.85483871 | Down |
| 7040 | ENSG00000105329 | K13375 | TGFB1 | chr19 | 41330531 | 41353933 | -3.963194153 | 4.325612745 | 0.00000705 | 0.000118 | 0.443589744 | 3.058974359 | Down |
| 1954 | ENSG00000105429 | - | MEGF8 | chr19 | 42325609 | 42378769 | -3.784387052 | 4.376575437 | 0.0000218 | 0.000343343 | 0.390509666 | 2.598242531 | Down |
| 495 | ENSG00000105675 | K01542 | ATP4A |  |  |  | -3.684703373 | 4.950329489 | 0.000299948 | 0.003768928 | 0.439613527 | 2.926570048 | Down |
| 136319 | ENSG00000105887 | - | MTPN | chr7 | 135926755 | 135977456 | -4.688341052 | 3.739046202 | 5.44E-14 | 3.28E-12 | 0.474576271 | 2.847457627 | Down |
| 11033 | ENSG00000105963 | - | ADAP1 | chr7 | 897900 | 955407 | -4.136763617 | 3.326189908 | 1.29E-12 | 5.09E-11 | 0.454545455 | 3.27012987 | Down |
| 8887 | ENSG00000106052 | K21347 | TAX1BP1 | chr7 | 27739373 | 27829767 | -3.229789215 | 4.563637906 | 0.000894949 | 0.009774872 | 0.611316113 | 3.027060271 | Down |
| 6804 | ENSG00000106089 | K04560 | STX1A |  |  |  | -4.136754236 | 3.326189908 | 1.3E-12 | 5.14E-11 | 0.621527778 | 2.920138889 | Down |
| 4885 | ENSG00000106236 | - | NPTX2 |  |  |  | -3.095843427 | 2.674084864 | 0.00000189 | 0.0000369 | 0.454756381 | 2.802784223 | Down |
| 3614 | ENSG00000106348 | K00088 | IMPDH1 | chr7 | 128392277 | 128409988 | -3.229789784 | 4.563637906 | 0.00089527 | 0.009774872 | 0.440734558 | 2.731218698 | Down |
| 95681 | ENSG00000106477 | K16455 | CEP41 | chr7 | 130393771 | 130441210 | -5.131855677 | 4.096541259 | 6.6E-15 | 8.93E-13 | 0.530831099 | 2.836461126 | Down |
| 643641 | ENSG00000106479 | - | ZNF862 | chr7 | 149838420 | 149867479 | -4.136744865 | 3.326189908 | 1.32E-12 | 5.18E-11 | 0.458511548 | 2.997433704 | Down |
| 79937 | ENSG00000106714 | - | CNTNAP3 |  |  |  | -3.09584028 | 2.674084864 | 0.00000189 | 0.0000369 | 0.447204969 | 2.802018634 | Down |
| 23731 | ENSG00000106771 | - | TMEM245 | chr9 | 109015135 | 109119963 | -5.1318511 | 4.096541259 | 6.67E-15 | 8.93E-13 | 0.353811149 | 3.135381115 | Down |
| 22949 | ENSG00000106853 | K13948 | PTGR1 | chr9 | 111549722 | 111599855 | -3.624675044 | 4.425833018 | 0.0000524 | 0.000757196 | 0.41337386 | 2.933130699 | Down |
| 23268 | ENSG00000107554 | K20705 | DNMBP | chr10 | 99875571 | 100009953 | -5.131846526 | 4.096541259 | 6.73E-15 | 8.93E-13 | 0.547875713 | 2.939759036 | Down |
| 64081 | ENSG00000108187 | - | PBLD |  |  |  | -3.480512715 | 4.47349386 | 0.000101085 | 0.001398117 | 0.447916667 | 2.920138889 | Down |
| 443 | ENSG00000108381 | K01437 | ASPA | chr17 | 3474106 | 3502534 | -4.136735505 | 3.326189908 | 1.34E-12 | 5.23E-11 | 0.476038339 | 3.134185304 | Down |
| 79665 | ENSG00000108406 | K18711 | DHX40 | chr17 | 59565525 | 59608352 | -3.262580629 | 2.765916931 | 0.000000967 | 0.000022 | 0.46084724 | 3.02182285 | Down |
| 57508 | ENSG00000108506 | K13139 | INTS2 | chr17 | 61865367 | 61928029 | -3.095837135 | 2.674084864 | 0.0000019 | 0.0000369 | 0.46013289 | 2.950166113 | Down |
| 4669 | ENSG00000108784 | K01205 | NAGLU | chr17 | 42535933 | 42544449 | -5.131841955 | 4.096541259 | 6.8E-15 | 8.93E-13 | 0.402422611 | 3.00538358 | Down |
| 9120 | ENSG00000108932 | K08183 | SLC16A6 | chr17 | 68267026 | 68291264 | -3.095833994 | 2.674084864 | 0.0000019 | 0.0000369 | 0.365200765 | 3.038240918 | Down |
| 1949 | ENSG00000108947 | K05463 | EFNB3 |  |  |  | -3.262576635 | 2.765916931 | 0.00000097 | 0.000022 | 0.35 | 2.658823529 | Down |
| 27346 | ENSG00000109084 | - | TMEM97 |  |  |  | -5.131837387 | 4.096541259 | 6.86E-15 | 8.93E-13 | 0.352272727 | 3.670454545 | Down |
| 4790 | ENSG00000109320 | K02580 | NFKB1 | chr4 | 102501329 | 102617302 | -3.517007966 | 4.026762593 | 0.0000472 | 0.00069757 | 0.504643963 | 2.790505676 | Down |
| 5602 | ENSG00000109339 | K04440 | MAPK10 |  |  |  | -3.095830855 | 2.674084864 | 0.0000019 | 0.0000369 | 0.497844828 | 3.040948276 | Down |
| 23158 | ENSG00000109436 | K19951 | TBC1D9 | chr4 | 140620782 | 140756317 | -4.747890975 | 3.785577849 | 8.62E-14 | 4.22E-12 | 0.531595577 | 3.033175355 | Down |
| 11199 | ENSG00000109511 | K17097 | ANXA10 |  |  |  | -3.383172318 | 5.051423819 | 0.000513738 | 0.005942872 | 0.50617284 | 3.080246914 | Down |
| 51166 | ENSG00000109576 | K00825 | AADAT |  |  |  | -3.095825501 | 2.674084864 | 0.00000191 | 0.000037 | 0.44988345 | 3.016317016 | Down |
| 23321 | ENSG00000109654 | K11997 | TRIM2 | chr4 | 153152281 | 153339322 | -4.136724544 | 3.326189908 | 1.36E-12 | 5.29E-11 | 0.492866407 | 2.792477302 | Down |
| 2743 | ENSG00000109738 | K05196 | GLRB | chr4 | 157076125 | 157172090 | -3.262572643 | 2.765916931 | 0.000000973 | 0.0000221 | 0.442655936 | 3.134808853 | Down |
| 7704 | ENSG00000109906 | K10055 | ZBTB16 |  |  |  | -3.09582015 | 2.674084864 | 0.00000192 | 0.0000371 | 0.499257058 | 2.778603269 | Down |
| 27087 | ENSG00000109956 | K00735 | B3GAT1 |  |  |  | -4.136711835 | 3.326189908 | 1.38E-12 | 5.36E-11 | 0.437125749 | 3.128742515 | Down |
| 9404 | ENSG00000110031 | - | LPXN |  |  |  | -3.095814803 | 2.674084864 | 0.00000192 | 0.0000371 | 0.485933504 | 2.920716113 | Down |
| 51338 | ENSG00000110079 | - | MS4A4A | chr11 | 60280541 | 60308972 | -3.034958286 | 4.206322663 | 0.000638041 | 0.007329241 | 0.355648536 | 2.707112971 | Down |
| 9828 | ENSG00000110237 | K20689 | ARHGEF17 | chr11 | 73305961 | 73369380 | -3.936974682 | 4.93513404 | 0.000119496 | 0.001633069 | 0.480853127 | 2.642753272 | Down |
| 50862 | ENSG00000110315 | - | RNF141 | chr11 | 10511678 | 10541227 | -3.691614617 | 5.387008694 | 0.000240441 | 0.003120225 | 0.539130435 | 2.760869565 | Down |
| 5818 | ENSG00000110400 | K06081 | NECTIN1 | chr11 | 119638098 | 119728725 | -3.691614201 | 5.387008694 | 0.000240321 | 0.003120225 | 0.500967118 | 2.889748549 | Down |
| 10944 | ENSG00000110696 | - | C11orf58 |  |  |  | -4.136705148 | 3.326189908 | 1.39E-12 | 5.39E-11 | 0.754098361 | 2.601092896 | Down |
| 9976 | ENSG00000110852 | K10071 | CLEC2B | chr12 | 9852369 | 9869859 | -4.064735591 | 3.280835385 | 5.6E-09 | 0.000000153 | 0.476510067 | 3.093959732 | Down |
| 23371 | ENSG00000111077 | K18080 | TNS2 |  |  |  | -3.095809459 | 2.674084864 | 0.00000193 | 0.0000372 | 0.429175476 | 2.778717407 | Down |
| 6539 | ENSG00000111181 | K05039 | SLC6A12 |  |  |  | -4.688335626 | 3.739046202 | 5.49E-14 | 3.3E-12 | 0.374592834 | 3.267100977 | Down |
| 59341 | ENSG00000111199 | K04973 | TRPV4 | chr12 | 109783087 | 109833407 | -4.064731315 | 3.280835385 | 5.63E-09 | 0.000000153 | 0.452353617 | 3.096440873 | Down |
| 81539 | ENSG00000111371 | K14990 | SLC38A1 | chr12 | 46183055 | 46269425 | -2.9619523 | 3.698629645 | 0.000495956 | 0.00591586 | 0.415506958 | 3.149105368 | Down |
| 4739 | ENSG00000111859 | K16832 | NEDD9 | chr6 | 11183298 | 11382348 | -5.683914411 | 4.568749497 | 1.84E-15 | 3.58E-13 | 0.511990408 | 2.882494005 | Down |
| 51020 | ENSG00000111906 | K07023 | HDDC2 | chr6 | 125274934 | 125302141 | -3.963196257 | 4.325612745 | 0.00000706 | 0.00011801 | 0.553921569 | 2.980392157 | Down |
| 3605 | ENSG00000112115 | K05489 | IL17A |  |  |  | -3.262568654 | 2.765916931 | 0.000000976 | 0.0000221 | 0.44516129 | 2.922580645 | Down |
| 7803 | ENSG00000112245 | K18041 | PTP4A1 | chr6 | 63528021 | 63583588 | -3.262564666 | 2.765916931 | 0.00000098 | 0.0000221 | 0.416184971 | 3.092485549 | Down |
| 11149 | ENSG00000112276 | K21108 | BVES | chr6 | 105096822 | 105137174 | -3.095804118 | 2.674084864 | 0.00000194 | 0.0000373 | 0.469444444 | 3.166666667 | Down |
| 9444 | ENSG00000112531 | K14945 | QKI | chr6 | 163414486 | 163578596 | -4.136698467 | 3.326189908 | 1.41E-12 | 5.43E-11 | 0.442815249 | 2.900293255 | Down |
| 4188 | ENSG00000112559 | - | MDFI | chr6 | 41637020 | 41654246 | -3.784388083 | 4.376575437 | 0.0000218 | 0.000343343 | 0.479674797 | 2.203252033 | Down |
| 10279 | ENSG00000112812 | K09649 | PRSS16 | chr6 | 27247701 | 27256620 | -5.131832822 | 4.096541259 | 6.93E-15 | 8.93E-13 | 0.383268482 | 2.758754864 | Down |
| 9037 | ENSG00000112902 | K06841 | SEMA5A | chr5 | 9035026 | 9546121 | -3.517010499 | 4.026762593 | 0.0000473 | 0.000697614 | 0.47858473 | 2.900372439 | Down |
| 1839 | ENSG00000113070 | K08523 | HBEGF |  |  |  | -4.064727331 | 3.280835385 | 5.66E-09 | 0.000000153 | 0.389423077 | 2.899038462 | Down |
| 3593 | ENSG00000113302 | K05425 | IL12B | chr5 | 159314783 | 159330473 | -4.136689769 | 3.326189908 | 1.42E-12 | 5.47E-11 | 0.554878049 | 3.018292683 | Down |
| 80315 | ENSG00000113742 | K02602 | CPEB4 | chr5 | 173888328 | 173960991 | -5.13182826 | 4.096541259 | 6.99E-15 | 8.93E-13 | 0.471879287 | 2.85048011 | Down |
| 3274 | ENSG00000113749 | K04150 | HRH2 |  |  |  | -3.095798781 | 2.674084864 | 0.00000194 | 0.0000373 | 0.377833753 | 3.083123426 | Down |
| 3827 | ENSG00000113889 | K03898 | KNG1 | chr3 | 186717309 | 186744410 | -3.095793448 | 2.674084864 | 0.00000195 | 0.0000374 | 0.540372671 | 2.872670807 | Down |
| 3081 | ENSG00000113924 | K00451 | HGD | chr3 | 120628168 | 120682571 | -4.688330203 | 3.739046202 | 5.54E-14 | 3.31E-12 | 0.449438202 | 3.047191011 | Down |
| 8850 | ENSG00000114166 | K06062 | KAT2B | chr3 | 20040032 | 20154404 | -4.518264877 | 4.737240222 | 0.00000058 | 0.0000136 | 0.449519231 | 2.984375 | Down |
| 10342 | ENSG00000114354 | K09292 | TFG | chr3 | 100709284 | 100748967 | -2.961955811 | 3.698629645 | 0.000496644 | 0.00591586 | 0.5525 | 2.7325 | Down |
| 79858 | ENSG00000114670 | K20880 | NEK11 | chr3 | 131026850 | 131350465 | -3.262560681 | 2.765916931 | 0.000000983 | 0.0000221 | 0.592248062 | 3.017054264 | Down |
| 10057 | ENSG00000114770 | K05668 | ABCC5 |  |  |  | -3.095788118 | 2.674084864 | 0.00000196 | 0.0000375 | 0.439109255 | 2.997912317 | Down |
| 13 | ENSG00000114771 | K13616 | AADAC | chr3 | 151814008 | 151828488 | -3.399127695 | 4.125804243 | 0.000125605 | 0.001712463 | 0.443609023 | 3.245614035 | Down |
| 2976 | ENSG00000115207 | K15200 | GTF3C2 | chr2 | 27325849 | 27357034 | -3.338469658 | 4.089072688 | 0.000136156 | 0.001828784 | 0.442371021 | 2.907793633 | Down |
| 79745 | ENSG00000115295 | K10423 | CLIP4 |  |  |  | -4.688324784 | 3.739046202 | 5.59E-14 | 3.32E-12 | 0.470921986 | 2.74751773 | Down |
| 84695 | ENSG00000115318 | K00280 | LOXL3 | chr2 | 74532258 | 74555709 | -3.399129365 | 4.125804243 | 0.000125763 | 0.001712592 | 0.448871182 | 2.771580345 | Down |
| 56655 | ENSG00000115350 | K03506 | POLE4 | chr2 | 74958648 | 74969732 | -3.493130272 | 5.452154712 | 0.000440927 | 0.005398657 | 0.444444444 | 2.538461538 | Down |
| 6869 | ENSG00000115353 | K04222 | TACR1 | chr2 | 75046463 | 75199519 | -3.095782791 | 2.674084864 | 0.00000196 | 0.0000375 | 0.42014742 | 3.223587224 | Down |
| 33 | ENSG00000115361 | K00255 | ACADL | chr2 | 210187990 | 210225491 | -5.131823701 | 4.096541259 | 7.06E-15 | 8.93E-13 | 0.430232558 | 2.930232558 | Down |
| 2744 | ENSG00000115419 | K01425 | GLS | chr2 | 190880821 | 190965552 | -3.517013031 | 4.026762593 | 0.0000473 | 0.000697728 | 0.428998505 | 2.865470852 | Down |
| 80303 | ENSG00000115468 | - | EFHD1 | chr2 | 232606057 | 232682781 | -3.963198765 | 4.325612745 | 0.00000707 | 0.000118055 | 0.514644351 | 2.953974895 | Down |
| 27013 | ENSG00000115649 | - | CNPPD1 |  |  |  | -4.13667868 | 3.326189908 | 1.44E-12 | 5.54E-11 | 0.414634146 | 2.97804878 | Down |
| 25823 | ENSG00000116176 | K09615 | TPSG1 |  |  |  | -6.128882116 | 4.964945407 | 5.75E-17 | 1.92E-14 | 0.345794393 | 2.666666667 | Down |
| 6814 | ENSG00000116266 | K15301 | STXBP3 | chr1 | 108746663 | 108809526 | -3.517015562 | 4.026762593 | 0.0000474 | 0.000697843 | 0.52027027 | 3.086148649 | Down |
| 79084 | ENSG00000116455 | K13221 | WDR77 | chr1 | 111439890 | 111449308 | -4.136667605 | 3.326189908 | 1.47E-12 | 5.6E-11 | 0.467836257 | 2.713450292 | Down |
| 9927 | ENSG00000116688 | K06030 | MFN2 | chr1 | 11980181 | 12013515 | -4.064723349 | 3.280835385 | 5.69E-09 | 0.000000153 | 0.499339498 | 3.025099075 | Down |
| 84618 | ENSG00000116981 | K01081 | NT5C1A | chr1 | 39659121 | 39672038 | -4.747885233 | 3.785577849 | 8.71E-14 | 4.25E-12 | 0.467391304 | 2.983695652 | Down |
| 6675 | ENSG00000117143 | K00972 | UAP1 | chr1 | 162561440 | 162599843 | -3.624675973 | 4.425833018 | 0.0000524 | 0.000757196 | 0.482758621 | 3.038314176 | Down |
| 117178 | ENSG00000117155 | K06085 | SSX2IP | chr1 | 84643707 | 84690757 | -5.131819145 | 4.096541259 | 7.12E-15 | 8.93E-13 | 0.625407166 | 2.990228013 | Down |
| 5129 | ENSG00000117266 | K15596 | CDK18 | chr1 | 205504556 | 205532793 | -3.963201199 | 4.325612745 | 0.00000709 | 0.000118094 | 0.468253968 | 3.073412698 | Down |
| 10327 | ENSG00000117448 | K00002 | AKR1A1 | chr1 | 45550779 | 45570051 | -3.963204288 | 4.325612745 | 0.0000071 | 0.00011819 | 0.446153846 | 3.070769231 | Down |
| 10560 | ENSG00000117479 | K14610 | SLC19A2 | chr1 | 169463909 | 169485970 | -3.624676901 | 4.425833018 | 0.0000525 | 0.000757196 | 0.38028169 | 3.193158954 | Down |
| 23632 | ENSG00000118298 | K01672 | CA14 | chr1 | 150257255 | 150265078 | -2.96195932 | 3.698629645 | 0.000497332 | 0.00591586 | 0.483679525 | 3.014836795 | Down |
| 57558 | ENSG00000118369 | K11854 | USP35 | chr11 | 78188812 | 78233651 | -3.095777468 | 2.674084864 | 0.00000197 | 0.0000376 | 0.492141454 | 2.896856582 | Down |
| 6696 | ENSG00000118785 | K06250 | SPP1 | chr4 | 87975650 | 87983411 | -3.095770314 | 2.674084864 | 0.00000198 | 0.0000377 | 0.767515924 | 2.77388535 | Down |
| 64747 | ENSG00000118855 | - | MFSD1 | chr3 | 158801870 | 158829719 | -3.262556699 | 2.765916931 | 0.000000986 | 0.0000222 | 0.319066148 | 3.048638132 | Down |
| 196483 | ENSG00000118894 | - | EEF2KMT | chr16 | 5084298 | 5097820 | -3.229790352 | 4.563637906 | 0.000895591 | 0.009774872 | 0.466666667 | 2.96969697 | Down |
| 57864 | ENSG00000119457 | K14614 | SLC46A2 |  |  |  | -3.262552718 | 2.765916931 | 0.000000989 | 0.0000222 | 0.338947368 | 2.934736842 | Down |
| 85365 | ENSG00000119523 | K03843 | ALG2 | chr9 | 99216425 | 99221964 | -3.624678126 | 4.425833018 | 0.0000526 | 0.000757396 | 0.435096154 | 3.112980769 | Down |
| 83544 | ENSG00000119661 | K10411 | DNAL1 | chr14 | 73644875 | 73703728 | -3.784389465 | 4.376575437 | 0.0000219 | 0.000343343 | 0.510526316 | 3.031578947 | Down |
| 53335 | ENSG00000119866 | - | BCL11A | chr2 | 60451167 | 60553498 | -2.961962827 | 3.698629645 | 0.00049802 | 0.00591586 | 0.502994012 | 2.711377246 | Down |
| 9419 | ENSG00000119878 | - | CRIPT | chr2 | 46617172 | 46630176 | -5.744124968 | 4.621282224 | 1.57E-15 | 3.55E-13 | 0.435643564 | 2.742574257 | Down |
| 5507 | ENSG00000119938 | K07189 | PPP1R3C | chr10 | 91628440 | 91633101 | -4.136654269 | 3.326189908 | 1.49E-12 | 5.68E-11 | 0.498422713 | 3.126182965 | Down |
| 26123 | ENSG00000119977 | K19382 | TCTN3 | chr10 | 95663396 | 95694143 | -5.683914516 | 4.568749497 | 1.84E-15 | 3.58E-13 | 0.4288 | 2.8384 | Down |
| 4115 | ENSG00000120289 | - | MAGEB4 | chrX | 30241940 | 30244193 | -3.262544605 | 2.765916931 | 0.000000996 | 0.0000223 | 0.537572254 | 2.901734104 | Down |
| 84418 | ENSG00000120306 | - | CYSTM1 |  |  |  | -6.128879573 | 4.964945407 | 5.79E-17 | 1.92E-14 | 0.422680412 | 2.907216495 | Down |
| 9617 | ENSG00000120662 | K02835 | MTRF1 | chr13 | 41216369 | 41311794 | -4.136646253 | 3.326189908 | 1.51E-12 | 5.73E-11 | 0.521348315 | 3.146067416 | Down |
| 51019 | ENSG00000120860 | K18463 | CCDC53 | chr12 | 102012840 | 102062124 | -4.136638244 | 3.326189908 | 1.52E-12 | 5.77E-11 | 0.613402062 | 2.721649485 | Down |
| 1191 | ENSG00000120885 | K17252 | CLU | chr8 | 27596917 | 27616717 | -3.78439086 | 4.376575437 | 0.0000219 | 0.000343343 | 0.567928731 | 3.124721604 | Down |
| 5533 | ENSG00000120910 | K04348 | PPP3CC |  |  |  | -4.136630242 | 3.326189908 | 1.54E-12 | 5.81E-11 | 0.489443378 | 3.069097889 | Down |
| 59342 | ENSG00000121064 | K09646 | SCPEP1 | chr17 | 56978107 | 57006768 | -5.683911851 | 4.568749497 | 1.85E-15 | 3.58E-13 | 0.457964602 | 3.084070796 | Down |
| 113 | ENSG00000121281 | K08047 | ADCY7 | chr16 | 50246158 | 50318135 | -4.136622248 | 3.326189908 | 1.56E-12 | 5.86E-11 | 0.39537037 | 2.982407407 | Down |
| 55268 | ENSG00000121310 | - | ECHDC2 | chr1 | 52895910 | 52927212 | -3.9632073 | 4.325612745 | 0.00000712 | 0.000118229 | 0.356164384 | 2.640410959 | Down |
| 3764 | ENSG00000121361 | K05001 | KCNJ8 | chr12 | 21764955 | 21775593 | -3.19082741 | 4.579030301 | 0.000452765 | 0.005495138 | 0.431603774 | 3.018867925 | Down |
| 65982 | ENSG00000121413 | K09230 | ZSCAN18 | chr19 | 58083834 | 58118433 | -3.034959032 | 4.206322663 | 0.000638628 | 0.007329241 | 0.505300353 | 2.671378092 | Down |
| 81627 | ENSG00000121486 | - | TRMT1L | chr1 | 185118085 | 185157098 | -5.131814591 | 4.096541259 | 7.19E-15 | 8.93E-13 | 0.480218281 | 2.882673943 | Down |
| 1475 | ENSG00000121552 | K13907 | CSTA | chr3 | 122325164 | 122341969 | -4.136614261 | 3.326189908 | 1.57E-12 | 5.9E-11 | 0.571428571 | 3.020408163 | Down |
| 55116 | ENSG00000121775 | - | TMEM39B | chr1 | 32072031 | 32102866 | -3.963210151 | 4.325612745 | 0.00000713 | 0.000118229 | 0.37804878 | 3.231707317 | Down |
| 729230 | ENSG00000121807 | K04177 | CCR2 | chr3 | 46353744 | 46360940 | -4.136606282 | 3.326189908 | 1.59E-12 | 5.95E-11 | 0.334224599 | 3.181818182 | Down |
| 119587 | ENSG00000121898 | K08639 | CPXM2 |  |  |  | -3.262537599 | 2.765916931 | 0.000001 | 0.0000224 | 0.488095238 | 3.006613757 | Down |
| 5412 | ENSG00000122042 | - | UBL3 |  |  |  | -2.961966333 | 3.698629645 | 0.00049871 | 0.00591586 | 0.487179487 | 2.94017094 | Down |
| 130916 | ENSG00000122085 | K15032 | MTERF4 | chr2 | 241072168 | 241103367 | -3.39913137 | 4.125804243 | 0.000125954 | 0.001713103 | 0.62992126 | 2.997375328 | Down |
| 54440 | ENSG00000122122 | - | SASH3 | chrX | 129779916 | 129795201 | -4.136598311 | 3.326189908 | 1.61E-12 | 5.99E-11 | 0.565789474 | 2.755263158 | Down |
| 29066 | ENSG00000122299 | - | ZC3H7A | chr16 | 11750586 | 11797258 | -3.597072089 | 5.417399556 | 0.000512741 | 0.005942872 | 0.509783728 | 3.008238929 | Down |
| 48 | ENSG00000122729 | K01681 | ACO1 | chr9 | 32384603 | 32450836 | -4.322032224 | 5.217598863 | 0.0000233 | 0.000355448 | 0.445444319 | 2.98200225 | Down |
| 800 | ENSG00000122786 | K12327 | CALD1 |  |  |  | -3.095763166 | 2.674084864 | 0.00000199 | 0.0000378 | 0.713745271 | 2.941992434 | Down |
| 9469 | ENSG00000122863 | K01020 | CHST3 | chr10 | 71964362 | 72013564 | -3.095756025 | 2.674084864 | 0.000002 | 0.0000379 | 0.448851775 | 3.089770355 | Down |
| 28981 | ENSG00000122970 | K19677 | IFT81 | chr12 | 110124335 | 110218795 | -3.784392102 | 4.376575437 | 0.0000219 | 0.000343343 | 0.585798817 | 3.155325444 | Down |
| 84329 | ENSG00000122986 | - | HVCN1 |  |  |  | -4.136590346 | 3.326189908 | 1.63E-12 | 6.04E-11 | 0.41025641 | 3.318681319 | Down |
| 10212 | ENSG00000123136 | K13182 | DDX39A | chr19 | 14408798 | 14419383 | -3.963212095 | 4.325612745 | 0.00000714 | 0.000118229 | 0.515222482 | 3.107728337 | Down |
| 262 | ENSG00000123505 | K01611 | AMD1 | chr6 | 110814621 | 110895713 | -3.09575255 | 2.674084864 | 0.000002 | 0.0000379 | 0.532934132 | 3.134730539 | Down |
| 81579 | ENSG00000123739 | K01047 | PLA2G12A | chr4 | 109709989 | 109730086 | -3.095359343 | 4.240277523 | 0.000508296 | 0.00591586 | 0.470899471 | 2.835978836 | Down |
| 80776 | ENSG00000123810 | K16745 | B9D2 | chr19 | 41354417 | 41364540 | -4.239741748 | 4.857906377 | 0.0000034 | 0.0000585 | 0.365714286 | 2.92 | Down |
| 725 | ENSG00000123843 | K04003 | C4BPB | chr1 | 207088826 | 207099993 | -3.480513507 | 4.47349386 | 0.000101215 | 0.001398237 | 0.5 | 2.956349206 | Down |
| 27304 | ENSG00000124217 | K11996 | MOCS3 |  |  |  | -5.131810041 | 4.096541259 | 7.26E-15 | 8.93E-13 | 0.419565217 | 2.765217391 | Down |
| 55577 | ENSG00000124357 | K00884 | NAGK | chr2 | 71068246 | 71078868 | -4.688318539 | 3.739046202 | 5.65E-14 | 3.34E-12 | 0.430769231 | 2.735897436 | Down |
| 4201 | ENSG00000124733 | - | MEA1 | chr6 | 42980344 | 43016886 | -4.13658239 | 3.326189908 | 1.64E-12 | 6.08E-11 | 0.610810811 | 2.605405405 | Down |
| 4246 | ENSG00000124939 | - | SCGB2A1 | chr11 | 62208668 | 62213939 | -3.095749076 | 2.674084864 | 0.00000201 | 0.000038 | 0.473684211 | 3.052631579 | Down |
| 652 | ENSG00000125378 | K04662 | BMP4 |  |  |  | -5.744123807 | 4.621282224 | 1.58E-15 | 3.55E-13 | 0.450980392 | 3.004901961 | Down |
| 8603 | ENSG00000125386 | - | FAM193A | chr4 | 2596288 | 2732575 | -3.784393344 | 4.376575437 | 0.000022 | 0.000343343 | 0.554940711 | 2.76284585 | Down |
| 55154 | ENSG00000125459 | - | MSTO1 | chr1 | 155610170 | 155614967 | -3.60361866 | 4.976015926 | 0.00021643 | 0.002835563 | 0.436842105 | 2.824561404 | Down |
| 27178 | ENSG00000125571 | K05485 | IL37 | chr2 | 112908889 | 112918882 | -3.095743579 | 2.674084864 | 0.00000201 | 0.000038 | 0.481651376 | 2.885321101 | Down |
| 192669 | ENSG00000126070 | K11593 | AGO3 |  |  |  | -4.747879495 | 3.785577849 | 8.8E-14 | 4.28E-12 | 0.426744186 | 3.044186047 | Down |
| 79713 | ENSG00000126246 | - | IGFLR1 | chr19 | 35739250 | 35742450 | -3.047829513 | 4.636091453 | 0.00075127 | 0.008545699 | 0.366197183 | 2.735211268 | Down |
| 2123 | ENSG00000126860 | - | EVI2A |  |  |  | -3.095738085 | 2.674084864 | 0.00000202 | 0.0000381 | 0.505791506 | 2.884169884 | Down |
| 64768 | ENSG00000127080 | K10572 | IPPK |  |  |  | -4.339133585 | 4.775802366 | 0.00000179 | 0.0000358 | 0.466395112 | 3.077393075 | Down |
| 53834 | ENSG00000127418 | - | FGFRL1 | chr4 | 1011822 | 1026898 | -5.131805493 | 4.096541259 | 7.33E-15 | 8.93E-13 | 0.369047619 | 2.797619048 | Down |
| 7277 | ENSG00000127824 | K07374 | TUBA4A | chr2 | 219249711 | 219254608 | -4.964029023 | 5.094673907 | 5.42E-08 | 0.00000136 | 0.511160714 | 2.930803571 | Down |
| 100506136 | ENSG00000127922 | - | LOC100506136 | chr7 | 96621657 | 96664338 | -3.603619084 | 4.976015926 | 0.000216533 | 0.002835563 | 0.3671875 | 2.71875 | Down |
| 7732 | ENSG00000128482 | - | RNF112 | chr17 | 19407169 | 19417276 | -3.262534688 | 2.765916931 | 0.000001 | 0.0000224 | 0.374009509 | 2.697305864 | Down |
| 57464 | ENSG00000128578 | - | STRIP2 | chr7 | 129434433 | 129488399 | -5.131800949 | 4.096541259 | 7.4E-15 | 8.93E-13 | 0.504796163 | 3.098321343 | Down |
| 4189 | ENSG00000128590 | K09515 | DNAJB9 | chr7 | 108569745 | 108574850 | -3.963213567 | 4.325612745 | 0.00000715 | 0.000118229 | 0.547085202 | 3.031390135 | Down |
| 84952 | ENSG00000128849 | K21110 | CGNL1 | chr15 | 57376487 | 57550727 | -3.22979123 | 4.563637906 | 0.000896088 | 0.009774872 | 0.642857143 | 2.87250384 | Down |
| 90416 | ENSG00000128891 | - | C15orf57 |  |  |  | -3.262531778 | 2.765916931 | 0.00000101 | 0.0000224 | 0.571428571 | 2.84729064 | Down |
| 91860 | ENSG00000129007 | K02183 | CALML4 | chr15 | 68190705 | 68206110 | -4.136574441 | 3.326189908 | 1.66E-12 | 6.13E-11 | 0.494897959 | 2.892857143 | Down |
| 57153 | ENSG00000129353 | K15377 | SLC44A2 | chr19 | 10602445 | 10644559 | -3.26252887 | 2.765916931 | 0.00000101 | 0.0000224 | 0.362869198 | 3.250351617 | Down |
| 27180 | ENSG00000129450 | K06740 | SIGLEC9 | chr19 | 51124880 | 51140480 | -4.136566499 | 3.326189908 | 1.68E-12 | 6.17E-11 | 0.409185804 | 2.891440501 | Down |
| 58533 | ENSG00000129515 | K17920 | SNX6 |  |  |  | -5.131795994 | 4.096541259 | 7.48E-15 | 8.93E-13 | 0.550239234 | 3.043062201 | Down |
| 6035 | ENSG00000129538 | K01168 | RNASE1 |  |  |  | -6.128877032 | 4.964945407 | 5.83E-17 | 1.92E-14 | 0.474358974 | 2.852564103 | Down |
| 3929 | ENSG00000129988 | K05399 | LBP | chr20 | 38346411 | 38377011 | -2.961969837 | 3.698629645 | 0.000499399 | 0.00591586 | 0.455301455 | 3.027027027 | Down |
| 23677 | ENSG00000130147 | K20066 | SH3BP4 | chr2 | 234951984 | 235055714 | -3.262520875 | 2.765916931 | 0.00000102 | 0.0000225 | 0.488058152 | 2.969885774 | Down |
| 3949 | ENSG00000130164 | K12473 | LDLR | chr19 | 11089362 | 11133830 | -4.688312298 | 3.739046202 | 5.72E-14 | 3.36E-12 | 0.509493671 | 2.726793249 | Down |
| 7837 | ENSG00000130508 | K19511 | PXDN | chr2 | 1631887 | 1744852 | -4.136558564 | 3.326189908 | 1.7E-12 | 6.22E-11 | 0.473968898 | 2.899932387 | Down |
| 26152 | ENSG00000130684 | K09228 | ZNF337 |  |  |  | -3.095732595 | 2.674084864 | 0.00000203 | 0.0000382 | 0.436750999 | 3.027962716 | Down |
| 84326 | ENSG00000130731 | - | C16orf13 | chr16 | 634427 | 636366 | -6.128874491 | 4.964945407 | 5.87E-17 | 1.92E-14 | 0.351219512 | 2.765853659 | Down |
| 1349 | ENSG00000131174 | K02271 | COX7B |  |  |  | -5.131791042 | 4.096541259 | 7.55E-15 | 8.93E-13 | 0.4125 | 3.075 | Down |
| 11127 | ENSG00000131437 | K10394 | KIF3A | chr5 | 132688808 | 132737633 | -3.664125951 | 5.015888334 | 0.000178161 | 0.002358889 | 0.58953168 | 2.920110193 | Down |
| 79652 | ENSG00000131634 | - | TMEM204 | chr16 | 1528741 | 1555580 | -5.131786094 | 4.096541259 | 7.63E-15 | 8.93E-13 | 0.349557522 | 2.809734513 | Down |
| 84231 | ENSG00000131653 | K10646 | TRAF7 | chr16 | 2155752 | 2178129 | -3.42469001 | 4.860165797 | 0.000508562 | 0.00591586 | 0.489552239 | 2.86119403 | Down |
| 4131 | ENSG00000131711 | K10429 | MAP1B | chr5 | 72107268 | 72209570 | -3.095727108 | 2.674084864 | 0.00000203 | 0.0000382 | 0.649918963 | 2.757698541 | Down |
| 2330 | ENSG00000131781 | K00485 | FMO5 | chr1 | 147183963 | 147225798 | -4.136550637 | 3.326189908 | 1.72E-12 | 6.27E-11 | 0.454033771 | 3.088180113 | Down |
| 84261 | ENSG00000132004 | K10265 | FBXW9 | chr19 | 12688916 | 12696900 | -5.131779034 | 4.096541259 | 7.74E-15 | 8.96E-13 | 0.472803347 | 2.834728033 | Down |
| 23647 | ENSG00000132254 | K20314 | ARFIP2 | chr11 | 6474683 | 6481479 | -3.784394585 | 4.376575437 | 0.000022 | 0.000343343 | 0.516042781 | 2.885026738 | Down |
| 5562 | ENSG00000132356 | K07198 | PRKAA1 | chr5 | 40759379 | 40798195 | -5.131772764 | 4.096541259 | 7.84E-15 | 8.96E-13 | 0.496515679 | 3.048780488 | Down |
| 23277 | ENSG00000132361 | K03255 | CLUH |  |  |  | -4.136542718 | 3.326189908 | 1.73E-12 | 6.31E-11 | 0.493506494 | 2.951871658 | Down |
| 85013 | ENSG00000132406 | - | TMEM128 | chr4 | 4235542 | 4248265 | -3.262515234 | 2.765916931 | 0.00000102 | 0.0000226 | 0.387878788 | 3.333333333 | Down |
| 55915 | ENSG00000132434 | - | LANCL2 |  |  |  | -4.747873762 | 3.785577849 | 8.89E-14 | 4.3E-12 | 0.464444444 | 3.013333333 | Down |
| 64773 | ENSG00000132635 | - | PCED1A | chr20 | 2835314 | 2841243 | -4.784774955 | 5.124914931 | 0.000000167 | 0.00000407 | 0.396475771 | 3.162995595 | Down |
| 222 | ENSG00000132746 | K00129 | ALDH3B2 | chr11 | 67662162 | 67681214 | -3.262509597 | 2.765916931 | 0.00000102 | 0.0000226 | 0.451948052 | 2.974025974 | Down |
| 10955 | ENSG00000132824 | - | SERINC3 | chr20 | 44496221 | 44522116 | -5.131768918 | 4.096541259 | 7.91E-15 | 8.96E-13 | 0.410147992 | 3.086680761 | Down |
| 56923 | ENSG00000132911 | K05053 | NMUR2 | chr5 | 152391541 | 152405279 | -2.961973339 | 3.698629645 | 0.00050009 | 0.00591586 | 0.378313253 | 3.339759036 | Down |
| 4628 | ENSG00000133026 | K10352 | MYH10 | chr17 | 8474205 | 8631369 | -4.136534805 | 3.326189908 | 1.75E-12 | 6.36E-11 | 0.608868959 | 3.031390135 | Down |
| 10631 | ENSG00000133110 | - | POSTN | chr13 | 37562582 | 37598844 | -4.238374536 | 3.40286445 | 5.56E-09 | 0.000000153 | 0.483253589 | 2.976076555 | Down |
| 66035 | ENSG00000133460 | K08148 | SLC2A11 | chr22 | 23856703 | 23886112 | -4.688306758 | 3.739046202 | 5.77E-14 | 3.38E-12 | 0.325233645 | 2.796261682 | Down |
| 102724197 | ENSG00000133475 | - | LOC102724197 | chr22 | 6 | 18670 | -3.095721625 | 2.674084864 | 0.00000204 | 0.0000383 | 0.414762742 | 2.855887522 | Down |
| 4922 | ENSG00000133636 | K05235 | NTS |  |  |  | -4.064719369 | 3.280835385 | 5.92E-09 | 0.000000157 | 0.505882353 | 3.164705882 | Down |
| 10894 | ENSG00000133800 | K19012 | LYVE1 |  |  |  | -4.13652555 | 3.326189908 | 1.78E-12 | 6.42E-11 | 0.48447205 | 2.854037267 | Down |
| 83876 | ENSG00000134042 | - | MRO | chr18 | 50795120 | 50825413 | -3.095716147 | 2.674084864 | 0.00000205 | 0.0000384 | 0.477099237 | 3.15648855 | Down |
| 8932 | ENSG00000134046 | K11590 | MBD2 | chr18 | 54151601 | 54224788 | -3.399132917 | 4.125804243 | 0.000126102 | 0.001713103 | 0.420924574 | 2.450121655 | Down |
| 8536 | ENSG00000134072 | K08794 | CAMK1 | chr3 | 9757345 | 9769987 | -5.131765073 | 4.096541259 | 7.97E-15 | 8.96E-13 | 0.502702703 | 2.975675676 | Down |
| 10100 | ENSG00000134198 | K17354 | TSPAN2 |  |  |  | -3.095708989 | 2.674084864 | 0.00000206 | 0.0000385 | 0.334841629 | 2.972850679 | Down |
| 84722 | ENSG00000134222 | K21443 | PSRC1 |  |  |  | -4.136516305 | 3.326189908 | 1.8E-12 | 6.48E-11 | 0.426997245 | 2.603305785 | Down |
| 5738 | ENSG00000134247 | K06729 | PTGFRN | chr1 | 116909923 | 116990358 | -3.480514299 | 4.47349386 | 0.000101346 | 0.001398286 | 0.499431172 | 2.948805461 | Down |
| 8774 | ENSG00000134265 | K21198 | NAPG | chr18 | 10525876 | 10552769 | -3.229791444 | 4.563637906 | 0.000896209 | 0.009774872 | 0.53525641 | 2.884615385 | Down |
| 51303 | ENSG00000134285 | K09576 | FKBP11 | chr12 | 48921959 | 48925547 | -4.624827564 | 5.154542875 | 0.00000041 | 0.00000981 | 0.358208955 | 3 | Down |
| 29841 | ENSG00000134317 | K09275 | GRHL1 | chr2 | 9951663 | 10002284 | -3.338471186 | 4.089072688 | 0.000136309 | 0.001828784 | 0.542071197 | 3.033980583 | Down |
| 1794 | ENSG00000134516 | K12367 | DOCK2 | chr5 | 169637247 | 170083382 | -2.961976839 | 3.698629645 | 0.000500781 | 0.00591586 | 0.515300546 | 3.177595628 | Down |
| 85004 | ENSG00000134533 | K07855 | RERG |  |  |  | -3.262503965 | 2.765916931 | 0.00000103 | 0.0000227 | 0.522613065 | 2.964824121 | Down |
| 90853 | ENSG00000134668 | K17596 | SPOCD1 |  |  |  | -4.136507069 | 3.326189908 | 1.82E-12 | 6.54E-11 | 0.435032895 | 2.611842105 | Down |
| 192670 | ENSG00000134698 | K11593 | AGO4 | chr1 | 35807630 | 35857890 | -3.811390917 | 5.350646805 | 0.000259111 | 0.003301078 | 0.433217189 | 3.009291521 | Down |
| 29015 | ENSG00000134802 | K08230 | SLC43A3 | chr11 | 57406954 | 57427580 | -3.229791658 | 4.563637906 | 0.00089633 | 0.009774872 | 0.361111111 | 3.138888889 | Down |
| 2694 | ENSG00000134812 | K14615 | GIF | chr11 | 59829268 | 59845529 | -4.784775256 | 5.124914931 | 0.000000167 | 0.00000407 | 0.482014388 | 2.836930456 | Down |
| 187 | ENSG00000134817 | K04174 | APLNR | chr11 | 57233577 | 57237453 | -3.338472714 | 4.089072688 | 0.000136463 | 0.001828784 | 0.413157895 | 3.086842105 | Down |
| 5156 | ENSG00000134853 | K04363 | PDGFRA |  |  |  | -4.136500097 | 3.326189908 | 1.84E-12 | 6.58E-11 | 0.548209366 | 3.011937557 | Down |
| 54841 | ENSG00000134897 | - | BIVM | chr13 | 102799049 | 102841538 | -4.136493131 | 3.326189908 | 1.85E-12 | 6.62E-11 | 0.502982107 | 2.964214712 | Down |
| 10677 | ENSG00000135407 | K08017 | AVIL |  |  |  | -4.136486171 | 3.326189908 | 1.87E-12 | 6.67E-11 | 0.514041514 | 3.003663004 | Down |
| 10220 | ENSG00000135414 | K05497 | GDF11 | chr12 | 55743278 | 55757194 | -5.131759753 | 4.096541259 | 8.06E-15 | 8.96E-13 | 0.41031941 | 2.901719902 | Down |
| 84872 | ENSG00000135482 | - | ZC3H10 | chr12 | 56118220 | 56122497 | -5.131755799 | 4.096541259 | 8.13E-15 | 8.96E-13 | 0.428571429 | 2.585253456 | Down |
| 6610 | ENSG00000135587 | K12351 | SMPD2 | chr6 | 109440646 | 109443922 | -5.131750212 | 4.096541259 | 8.22E-15 | 8.97E-13 | 0.411347518 | 3.089834515 | Down |
| 84865 | ENSG00000135637 | - | CCDC142 |  |  |  | -4.136479216 | 3.326189908 | 1.89E-12 | 6.71E-11 | 0.394666667 | 2.821333333 | Down |
| 10753 | ENSG00000135773 | K08578 | CAPN9 | chr1 | 230747289 | 230803356 | -3.493130161 | 5.452154712 | 0.000440851 | 0.005398657 | 0.513043478 | 3.142028986 | Down |
| 3915 | ENSG00000135862 | K05635 | LAMC1 | chr1 | 183023460 | 183145592 | -3.262498338 | 2.765916931 | 0.00000103 | 0.0000228 | 0.557489124 | 2.679303915 | Down |
| 55619 | ENSG00000135905 | - | DOCK10 |  |  |  | -3.262492715 | 2.765916931 | 0.00000104 | 0.0000228 | 0.530649588 | 3.064958829 | Down |
| 9654 | ENSG00000135912 | K16601 | TTLL4 | chr2 | 218710835 | 218757484 | -3.095701838 | 2.674084864 | 0.00000207 | 0.0000386 | 0.493744787 | 2.909924937 | Down |
| 9392 | ENSG00000135966 | K20177 | TGFBRAP1 | chr2 | 105249404 | 105329714 | -4.688300651 | 3.739046202 | 5.83E-14 | 3.39E-12 | 0.463953488 | 3.063953488 | Down |
| 84102 | ENSG00000136052 | K15122 | SLC41A2 | chr12 | 104802553 | 104958744 | -4.518266362 | 4.737240222 | 0.000000581 | 0.0000136 | 0.417102967 | 2.97556719 | Down |
| 101927181 | ENSG00000136213 | - | LOC101927181 | chr7 | 2437763 | 2447850 | -3.443660464 | 5.089316372 | 0.000425207 | 0.005219942 | 0.439613527 | 3.306763285 | Down |
| 5879 | ENSG00000136238 | K04392 | RAC1 | chr7 | 6374495 | 6403967 | -3.48051509 | 4.47349386 | 0.000101477 | 0.001398286 | 0.431279621 | 3 | Down |
| 58489 | ENSG00000136379 | - | ABHD17C | chr15 | 80695311 | 80755621 | -3.517018092 | 4.026762593 | 0.0000475 | 0.000697958 | 0.43768997 | 2.835866261 | Down |
| 55852 | ENSG00000136478 | - | TEX2 | chr17 | 64147433 | 64263323 | -4.136472267 | 3.326189908 | 1.91E-12 | 6.75E-11 | 0.546737213 | 2.910934744 | Down |
| 7111 | ENSG00000136842 | K10370 | TMOD1 |  |  |  | -4.136465323 | 3.326189908 | 1.93E-12 | 6.79E-11 | 0.576601671 | 2.955431755 | Down |
| 158427 | ENSG00000136925 | - | TSTD2 | chr9 | 97600080 | 97633680 | -3.57756801 | 4.065055456 | 0.0000384 | 0.00057497 | 0.494186047 | 2.994186047 | Down |
| 9380 | ENSG00000137106 | K00049 | GRHPR | chr9 | 37422666 | 37437782 | -3.784395826 | 4.376575437 | 0.000022 | 0.000343343 | 0.413407821 | 2.751396648 | Down |
| 7280 | ENSG00000137267 | K07375 | TUBB2A | chr6 | 3153666 | 3157549 | -3.262487096 | 2.765916931 | 0.00000104 | 0.0000229 | 0.548314607 | 2.912359551 | Down |
| 347733 | ENSG00000137285 | K07375 | TUBB2B | chr6 | 3224261 | 3227734 | -3.262481482 | 2.765916931 | 0.00000105 | 0.0000229 | 0.548314607 | 2.914606742 | Down |
| 11309 | ENSG00000137491 | K14352 | SLCO2B1 |  |  |  | -4.688294549 | 3.739046202 | 5.89E-14 | 3.41E-12 | 0.359661495 | 2.880112835 | Down |
| 5547 | ENSG00000137509 | K01285 | PRCP | chr11 | 82822940 | 82901740 | -3.095694693 | 2.674084864 | 0.00000208 | 0.0000387 | 0.429400387 | 3.073500967 | Down |
| 5962 | ENSG00000137710 | K05762 | RDX | chr11 | 110174880 | 110296712 | -4.688288451 | 3.739046202 | 5.96E-14 | 3.43E-12 | 0.579470199 | 3.182119205 | Down |
| 100533181 | ENSG00000137726 | - | FXYD6-FXYD2 | chr11 | 117820075 | 117877031 | -3.034959778 | 4.206322663 | 0.000639215 | 0.007329241 | 0.432432432 | 3.009009009 | Down |
| 51496 | ENSG00000137770 | K17616 | CTDSPL2 | chr15 | 44427329 | 44527774 | -5.13174463 | 4.096541259 | 8.32E-15 | 8.97E-13 | 0.564377682 | 3.012875536 | Down |
| 6176 | ENSG00000137818 | K02942 | RPLP1 | chr15 | 69452820 | 69455545 | -3.229791872 | 4.563637906 | 0.000896451 | 0.009774872 | 0.456140351 | 2.447368421 | Down |
| 3706 | ENSG00000137825 | K00911 | ITPKA |  |  |  | -4.136458385 | 3.326189908 | 1.94E-12 | 6.84E-11 | 0.46637744 | 2.800433839 | Down |
| 53905 | ENSG00000137857 | K13411 | DUOX1 |  |  |  | -5.131740566 | 4.096541259 | 8.39E-15 | 8.97E-13 | 0.439071567 | 3.152804642 | Down |
| 64220 | ENSG00000137868 | - | STRA6 | chr15 | 74179466 | 74212267 | -4.136451453 | 3.326189908 | 1.96E-12 | 6.88E-11 | 0.342776204 | 3.005665722 | Down |
| 145781 | ENSG00000137878 | - | GCOM1 |  |  |  | -5.131736506 | 4.096541259 | 8.46E-15 | 8.97E-13 | 0.624836601 | 2.907189542 | Down |
| 2644 | ENSG00000137880 | - | GCHFR | chr15 | 40764087 | 40767713 | -3.190827452 | 4.579030301 | 0.000452831 | 0.005495138 | 0.476190476 | 3.095238095 | Down |
| 1629 | ENSG00000137992 | K09699 | DBT | chr1 | 100186922 | 100249864 | -3.684703903 | 4.950329489 | 0.000300107 | 0.003768928 | 0.443983402 | 2.997925311 | Down |
| 9581 | ENSG00000138078 | - | PREPL | chr2 | 44317607 | 44361862 | -2.961979806 | 3.698629645 | 0.000501367 | 0.00591586 | 0.525447043 | 3.182943604 | Down |
| 57159 | ENSG00000138100 | K10653 | TRIM54 | chr2 | 27282429 | 27307439 | -2.961982771 | 3.698629645 | 0.000501954 | 0.00591586 | 0.5425 | 2.8275 | Down |
| 1559 | ENSG00000138109 | K17719 | CYP2C9 |  |  |  | -4.136442869 | 3.326189908 | 1.98E-12 | 6.94E-11 | 0.436734694 | 3.142857143 | Down |
| 10579 | ENSG00000138162 | K14282 | TACC2 | chr10 | 121989174 | 122254545 | -3.480515741 | 4.47349386 | 0.000101585 | 0.001398286 | 0.544097693 | 2.505427408 | Down |
| 6744 | ENSG00000138434 | - | SSFA2 |  |  |  | -4.136430186 | 3.326189908 | 2.02E-12 | 7.04E-11 | 0.60127085 | 2.685464654 | Down |
| 51191 | ENSG00000138646 | - | HERC5 | chr4 | 88456604 | 88506170 | -3.262475873 | 2.765916931 | 0.00000105 | 0.000023 | 0.474609375 | 3.1015625 | Down |
| 55132 | ENSG00000138709 | K18757 | LARP1B | chr4 | 128060655 | 128222931 | -3.963215061 | 4.325612745 | 0.00000716 | 0.000118229 | 0.583150985 | 3.036105033 | Down |
| 22915 | ENSG00000138722 | - | MMRN1 | chr4 | 89879539 | 89954629 | -2.961985736 | 3.698629645 | 0.000502541 | 0.00591586 | 0.540716612 | 2.924267101 | Down |
| 246175 | ENSG00000138767 | K12603 | CNOT6L |  |  |  | -4.136419246 | 3.326189908 | 2.05E-12 | 7.12E-11 | 0.471530249 | 3.069395018 | Down |
| 23710 | ENSG00000139112 | K08341 | GABARAPL1 | chr12 | 10212841 | 10223128 | -4.688282358 | 3.739046202 | 6.02E-14 | 3.45E-12 | 0.404109589 | 3.397260274 | Down |
| 121536 | ENSG00000139154 | K17452 | AEBP2 | chr12 | 19404101 | 19522239 | -4.13640832 | 3.326189908 | 2.08E-12 | 7.2E-11 | 0.564796905 | 2.419729207 | Down |
| 144165 | ENSG00000139174 | K04511 | PRICKLE1 | chr12 | 42458338 | 42589770 | -4.136395163 | 3.326189908 | 2.12E-12 | 7.31E-11 | 0.564380265 | 2.890493381 | Down |
| 196500 | ENSG00000139200 | - | PIANP | chr12 | 6689726 | 6700843 | -3.095687554 | 2.674084864 | 0.00000209 | 0.0000388 | 0.35106383 | 2.758865248 | Down |
| 1280 | ENSG00000139219 | K19719 | COL2A1 |  |  |  | -3.262470268 | 2.765916931 | 0.00000106 | 0.0000231 | 0.320107599 | 2.094821789 | Down |
| 7786 | ENSG00000139625 | K04423 | MAP3K12 | chr12 | 53480492 | 53501066 | -3.828687543 | 5.34566004 | 0.0000779 | 0.001101533 | 0.475336323 | 2.683856502 | Down |
| 283373 | ENSG00000139645 | K15504 | ANKRD52 | chr12 | 56237807 | 56258391 | -3.891373692 | 4.606796036 | 0.0000198 | 0.000315699 | 0.432156134 | 2.665427509 | Down |
| 161357 | ENSG00000139915 | - | MDGA2 | chr14 | 46836518 | 47675319 | -4.747867625 | 3.785577849 | 8.99E-14 | 4.33E-12 | 0.5041841 | 2.9958159 | Down |
| 6652 | ENSG00000140263 | K00008 | SORD | chr15 | 45023104 | 45075089 | -5.131732447 | 4.096541259 | 8.53E-15 | 8.97E-13 | 0.378151261 | 2.770308123 | Down |
| 405753 | ENSG00000140274 | K17232 | DUOXA2 |  |  |  | -4.136388414 | 3.326189908 | 2.14E-12 | 7.36E-11 | 0.334375 | 3.05 | Down |
| 54939 | ENSG00000140365 | - | COMMD4 |  |  |  | -5.131726493 | 4.096541259 | 8.63E-15 | 9E-13 | 0.421800948 | 2.706161137 | Down |
| 60677 | ENSG00000140488 | K13207 | CELF6 |  |  |  | -3.095676968 | 2.674084864 | 0.0000021 | 0.000039 | 0.363825364 | 2.654885655 | Down |
| 55114 | ENSG00000140750 | K20638 | ARHGAP17 | chr16 | 24919384 | 25015601 | -3.784397067 | 4.376575437 | 0.000022 | 0.000343343 | 0.464245176 | 2.742338252 | Down |
| 90507 | ENSG00000141295 | K14358 | SCRN2 | chr17 | 47837681 | 47841333 | -3.624679461 | 4.425833018 | 0.0000527 | 0.000757702 | 0.461893764 | 2.796766744 | Down |
| 85464 | ENSG00000141298 | K05766 | SSH2 | chr17 | 29625938 | 29930239 | -4.064714033 | 3.280835385 | 5.96E-09 | 0.000000158 | 0.577931034 | 2.792413793 | Down |
| 22901 | ENSG00000141337 | K12381 | ARSG |  |  |  | -3.095667628 | 2.674084864 | 0.00000211 | 0.0000392 | 0.420952381 | 2.853333333 | Down |
| 10351 | ENSG00000141338 | K05650 | ABCA8 | chr17 | 68867285 | 68955426 | -3.2624654 | 2.765916931 | 0.00000106 | 0.0000231 | 0.448488587 | 3.15299198 | Down |
| 3613 | ENSG00000141401 | K01092 | IMPA2 | chr18 | 11981428 | 12030886 | -3.262459001 | 2.765916931 | 0.00000107 | 0.0000232 | 0.427083333 | 2.774305556 | Down |
| 6563 | ENSG00000141469 | K08716 | SLC14A1 | chr18 | 45724123 | 45752520 | -4.136380212 | 3.326189908 | 2.16E-12 | 7.42E-11 | 0.352808989 | 2.937078652 | Down |
| 432 | ENSG00000141505 | K10063 | ASGR1 |  |  |  | -4.136373771 | 3.326189908 | 2.18E-12 | 7.46E-11 | 0.542955326 | 2.95532646 | Down |
| 6904 | ENSG00000141556 | - | TBCD | chr17 | 82752048 | 82943186 | -6.128871951 | 4.964945407 | 5.92E-17 | 1.92E-14 | 0.438211382 | 2.927642276 | Down |
| 22994 | ENSG00000141577 | K16540 | CEP131 |  |  |  | -4.136367336 | 3.326189908 | 2.2E-12 | 7.5E-11 | 0.561403509 | 2.788550323 | Down |
| 30811 | ENSG00000142149 | K08797 | HUNK | chr21 | 31873315 | 32004064 | -3.48051663 | 4.47349386 | 0.000101732 | 0.001398393 | 0.448179272 | 2.927170868 | Down |
| 8547 | ENSG00000142748 | K10104 | FCN3 | chr1 | 27369110 | 27374824 | -5.131720544 | 4.096541259 | 8.74E-15 | 9.03E-13 | 0.421404682 | 2.856187291 | Down |
| 64129 | ENSG00000142910 | - | TINAGL1 | chr1 | 31576384 | 31587686 | -5.131715793 | 4.096541259 | 8.83E-15 | 9.04E-13 | 0.415417559 | 2.875802998 | Down |
| 79590 | ENSG00000143314 | K02895 | MRPL24 | chr1 | 156737302 | 156741267 | -3.179034837 | 4.148862116 | 0.00034325 | 0.004281642 | 0.453703704 | 3.148148148 | Down |
| 58497 | ENSG00000143363 | K01514 | PRUNE | chr1 | 151008391 | 151035713 | -5.131710001 | 4.096541259 | 8.93E-15 | 9.07E-13 | 0.498896247 | 2.922737307 | Down |
| 9869 | ENSG00000143379 | K11421 | SETDB1 | chr1 | 150926246 | 150964744 | -4.784775558 | 5.124914931 | 0.000000167 | 0.00000407 | 0.541440744 | 2.824167312 | Down |
| 8394 | ENSG00000143398 | K00889 | PIP5K1A | chr1 | 151198003 | 151249536 | -3.034960605 | 4.206322663 | 0.000639867 | 0.007329391 | 0.494661922 | 2.960854093 | Down |
| 64241 | ENSG00000143921 | K05684 | ABCG8 | chr2 | 43831942 | 43880051 | -5.683909188 | 4.568749497 | 1.86E-15 | 3.58E-13 | 0.420505201 | 3.080237741 | Down |
| 130120 | ENSG00000143954 | - | REG3G |  |  |  | -3.262453296 | 2.765916931 | 0.00000107 | 0.0000233 | 0.457142857 | 2.908571429 | Down |
| 79998 | ENSG00000144031 | K21441 | ANKRD53 | chr2 | 70977183 | 70985499 | -3.262447755 | 2.765916931 | 0.00000108 | 0.0000233 | 0.426415094 | 2.962264151 | Down |
| 94097 | ENSG00000144040 | - | SFXN5 | chr2 | 72942036 | 73072234 | -3.480517562 | 4.47349386 | 0.000101887 | 0.001398393 | 0.385294118 | 2.935294118 | Down |
| 113419 | ENSG00000144043 | - | TEX261 |  |  |  | -4.747863889 | 3.785577849 | 9.05E-14 | 4.34E-12 | 0.357142857 | 3.551020408 | Down |
| 7851 | ENSG00000144063 | - | MALL |  |  |  | -4.747860156 | 3.785577849 | 9.11E-14 | 4.35E-12 | 0.346405229 | 3.483660131 | Down |
| 5899 | ENSG00000144118 | K07835 | RALB | chr2 | 120240070 | 120294711 | -4.68827627 | 3.739046202 | 6.08E-14 | 3.47E-12 | 0.567961165 | 2.990291262 | Down |
| 11159 | ENSG00000144134 | K07931 | RABL2A | chr2 | 113627218 | 113643398 | -4.13635465 | 3.326189908 | 2.24E-12 | 7.61E-11 | 0.596638655 | 3.084033613 | Down |
| 51601 | ENSG00000144182 | K10105 | LIPT1 | chr2 | 99154955 | 99163157 | -4.688270187 | 3.739046202 | 6.15E-14 | 3.49E-12 | 0.45844504 | 3.08310992 | Down |
| 5746 | ENSG00000144407 | K04586 | PTH2R |  |  |  | -3.095657566 | 2.674084864 | 0.00000213 | 0.0000393 | 0.414545455 | 3.127272727 | Down |
| 129563 | ENSG00000144535 | K18758 | DIS3L2 | chr2 | 231961583 | 232343968 | -4.688264108 | 3.739046202 | 6.21E-14 | 3.51E-12 | 0.505084746 | 2.962711864 | Down |
| 114790 | ENSG00000144589 | - | STK11IP | chr2 | 219597851 | 219616451 | -3.229792816 | 4.563637906 | 0.000896985 | 0.009774872 | 0.465878071 | 2.885350318 | Down |
| 29926 | ENSG00000144591 | K00966 | GMPPA |  |  |  | -5.131702776 | 4.096541259 | 9.07E-15 | 9.12E-13 | 0.397463002 | 2.978858351 | Down |
| 84892 | ENSG00000144647 | K18207 | POMGNT2 |  |  |  | -5.131698258 | 4.096541259 | 9.15E-15 | 9.13E-13 | 0.406896552 | 3.220689655 | Down |
| 1238 | ENSG00000144648 | K04187 | ACKR2 |  |  |  | -3.262444251 | 2.765916931 | 0.00000108 | 0.0000233 | 0.369791667 | 3.247395833 | Down |
| 3680 | ENSG00000144668 | K06585 | ITGA9 | chr3 | 37452322 | 37819790 | -5.131693744 | 4.096541259 | 9.24E-15 | 9.13E-13 | 0.465700483 | 2.92173913 | Down |
| 653333 | ENSG00000145002 | - | FAM86B2 | chr8 | 12424419 | 12436418 | -3.229793264 | 4.563637906 | 0.000897239 | 0.009774872 | 0.451515152 | 2.960606061 | Down |
| 4026 | ENSG00000145012 | K16676 | LPP | chr3 | 188153050 | 188890671 | -3.784398307 | 4.376575437 | 0.0000221 | 0.000343343 | 0.437908497 | 2.733660131 | Down |
| 9718 | ENSG00000145194 | K01415 | ECE2 | chr3 | 184249657 | 184293031 | -3.50579258 | 4.832318368 | 0.000667624 | 0.007624474 | 0.522083805 | 3.013590034 | Down |
| 10861 | ENSG00000145217 | K14700 | SLC26A1 | chr4 | 979073 | 993440 | -4.747855271 | 3.785577849 | 9.19E-14 | 4.37E-12 | 0.346647646 | 2.77746077 | Down |
| 345274 | ENSG00000145283 | K14346 | SLC10A6 | chr4 | 86823468 | 86849263 | -4.064708701 | 3.280835385 | 5.99E-09 | 0.000000158 | 0.360742706 | 2.933687003 | Down |
| 79966 | ENSG00000145284 | K00507 | SCD5 | chr4 | 82629537 | 82798857 | -5.131689001 | 4.096541259 | 9.33E-15 | 9.14E-13 | 0.363636364 | 3.193939394 | Down |
| 5921 | ENSG00000145715 | K04352 | RASA1 | chr5 | 87267801 | 87391926 | -2.961989741 | 3.698629645 | 0.000503336 | 0.00591586 | 0.47086915 | 2.959885387 | Down |
| 5066 | ENSG00000145730 | K00504 | PAM |  |  |  | -4.747850388 | 3.785577849 | 9.27E-14 | 4.39E-12 | 0.474332649 | 2.95687885 | Down |
| 23092 | ENSG00000145819 | K20071 | ARHGAP26 | chr5 | 142770370 | 143229007 | -3.239491202 | 4.184151988 | 0.000282186 | 0.003570862 | 0.534398034 | 2.998771499 | Down |
| 153830 | ENSG00000145860 | - | RNF145 | chr5 | 159157409 | 159210053 | -4.747845509 | 3.785577849 | 9.35E-14 | 4.41E-12 | 0.395382395 | 3.262626263 | Down |
| 26249 | ENSG00000146021 | K10443 | KLHL3 | chr5 | 137617500 | 137736090 | -4.688258035 | 3.739046202 | 6.28E-14 | 3.53E-12 | 0.495741056 | 2.856899489 | Down |
| 50636 | ENSG00000146205 | K19501 | ANO7 | chr2 | 241188509 | 241239602 | -5.131684261 | 4.096541259 | 9.42E-15 | 9.16E-13 | 0.407288317 | 3.129689175 | Down |
| 51390 | ENSG00000146416 | - | AIG1 | chr6 | 143059363 | 143343594 | -4.2383702 | 3.40286445 | 5.6E-09 | 0.000000153 | 0.351020408 | 3.359183673 | Down |
| 347404 | ENSG00000147036 | - | LANCL3 |  |  |  | -3.095647518 | 2.674084864 | 0.00000214 | 0.0000395 | 0.40952381 | 2.988095238 | Down |
| 114569 | ENSG00000147676 | - | MAL2 |  |  |  | -5.131679378 | 4.096541259 | 9.52E-15 | 9.17E-13 | 0.306818182 | 3.090909091 | Down |
| 56169 | ENSG00000147697 | - | GSDMC |  |  |  | -4.064704605 | 3.280835385 | 6.02E-09 | 0.000000159 | 0.501968504 | 3.098425197 | Down |
| 256158 | ENSG00000148357 | K17341 | HMCN2 | chr9 | 130265742 | 130434123 | -3.597072221 | 5.417399556 | 0.000512832 | 0.005942872 | 0.444158925 | 2.689464321 | Down |
| 92170 | ENSG00000148824 | K19828 | MTG1 | chr10 | 133394117 | 133420670 | -3.784399547 | 4.376575437 | 0.0000221 | 0.000343343 | 0.383233533 | 2.94011976 | Down |
| 2620 | ENSG00000148935 | - | GAS2 |  |  |  | -4.136341983 | 3.326189908 | 2.27E-12 | 7.72E-11 | 0.463258786 | 2.900958466 | Down |
| 54970 | ENSG00000149292 | - | TTC12 | chr11 | 113314529 | 113385952 | -4.747841207 | 3.785577849 | 9.43E-14 | 4.43E-12 | 0.509562842 | 2.887978142 | Down |
| 89944 | ENSG00000149328 | - | GLB1L2 | chr11 | 134331874 | 134376324 | -3.577570239 | 4.065055456 | 0.0000384 | 0.000574973 | 0.441823899 | 3.179245283 | Down |
| 51524 | ENSG00000149483 | - | TMEM138 | chr11 | 61362001 | 61376502 | -5.131674498 | 4.096541259 | 9.62E-15 | 9.19E-13 | 0.351851852 | 3.679012346 | Down |
| 219990 | ENSG00000149507 | - | OOSP2 |  |  |  | -3.262436384 | 2.765916931 | 0.00000109 | 0.0000235 | 0.506329114 | 3.120253165 | Down |
| 84304 | ENSG00000149761 | - | NUDT22 | chr11 | 64226258 | 64230016 | -3.603619508 | 4.976015926 | 0.000216636 | 0.002835563 | 0.435643564 | 2.778877888 | Down |
| 220108 | ENSG00000150510 | - | FAM124A | chr13 | 51222328 | 51284241 | -3.26242974 | 2.765916931 | 0.00000109 | 0.0000235 | 0.484536082 | 2.867697595 | Down |
| 775 | ENSG00000151067 | K04850 | CACNA1C | chr12 | 1969677 | 2697949 | -3.095639829 | 2.674084864 | 0.00000215 | 0.0000397 | 0.445903124 | 3.014938886 | Down |
| 100532726 | ENSG00000151364 | K03968 | NDUFC2-KCTD14 | chr11 | 78015715 | 78080219 | -4.179256663 | 4.813376398 | 0.00000442 | 0.0000752 | 0.470588235 | 3.266666667 | Down |
| 140609 | ENSG00000151414 | K20876 | NEK7 | chr1 | 198156978 | 198322420 | -4.578849299 | 4.784107766 | 0.000000468 | 0.0000111 | 0.456953642 | 3.105960265 | Down |
| 83447 | ENSG00000151475 | K05863 | SLC25A31 | chr4 | 127730375 | 127774299 | -3.095632148 | 2.674084864 | 0.00000216 | 0.0000398 | 0.352380952 | 3.076190476 | Down |
| 9781 | ENSG00000151692 | K11975 | RNF144A | chr2 | 6917392 | 7076886 | -4.136333784 | 3.326189908 | 2.3E-12 | 7.78E-11 | 0.424657534 | 2.928082192 | Down |
| 196394 | ENSG00000151743 | - | AMN1 |  |  |  | -4.064700511 | 3.280835385 | 6.05E-09 | 0.000000159 | 0.418604651 | 2.821705426 | Down |
| 130340 | ENSG00000152056 | K12395 | AP1S3 | chr2 | 223755330 | 223837602 | -5.131668551 | 4.096541259 | 9.74E-15 | 9.23E-13 | 0.5 | 3.347560976 | Down |
| 3087 | ENSG00000152804 | K08024 | HHEX |  |  |  | -3.0956263 | 2.674084864 | 0.00000217 | 0.0000399 | 0.5 | 2.948148148 | Down |
| 7402 | ENSG00000152818 | - | UTRN | chr6 | 144284955 | 144853034 | -5.131662609 | 4.096541259 | 9.86E-15 | 9.26E-13 | 0.590154384 | 3.011069036 | Down |
| 55203 | ENSG00000153012 | - | LGI2 |  |  |  | -3.26242456 | 2.765916931 | 0.0000011 | 0.0000236 | 0.491743119 | 3.157798165 | Down |
| 266977 | ENSG00000153292 | K08453 | ADGRF1 | chr6 | 46997703 | 47042363 | -5.131655201 | 4.096541259 | 1E-14 | 9.33E-13 | 0.421978022 | 3.045054945 | Down |
| 26175 | ENSG00000153485 | - | TMEM251 |  |  |  | -4.06469642 | 3.280835385 | 6.08E-09 | 0.00000016 | 0.390532544 | 3.278106509 | Down |
| 9407 | ENSG00000153802 | K09641 | TMPRSS11D | chr4 | 67820876 | 67885063 | -4.964028243 | 5.094673907 | 5.42E-08 | 0.00000136 | 0.454545455 | 2.868421053 | Down |
| 8526 | ENSG00000153933 | K00901 | DGKE | chr17 | 56834099 | 56869565 | -3.784400787 | 4.376575437 | 0.0000221 | 0.000343343 | 0.435626102 | 2.980599647 | Down |
| 2838 | ENSG00000154165 | K08403 | GPR15 |  |  |  | -5.131650061 | 4.096541259 | 1.01E-14 | 9.35E-13 | 0.369444444 | 3.230555556 | Down |
| 5578 | ENSG00000154229 | K02677 | PRKCA | chr17 | 66302640 | 66810744 | -4.688251966 | 3.739046202 | 6.34E-14 | 3.55E-12 | 0.477678571 | 3.098214286 | Down |
| 55286 | ENSG00000154274 | - | C4orf19 | chr4 | 37453454 | 37596146 | -3.624680796 | 4.425833018 | 0.0000528 | 0.000758009 | 0.601910828 | 2.563694268 | Down |
| 9510 | ENSG00000154734 | K08617 | ADAMTS1 |  |  |  | -3.095620456 | 2.674084864 | 0.00000218 | 0.00004 | 0.475698035 | 2.716649431 | Down |
| 7476 | ENSG00000154764 | K00572 | WNT7A | chr3 | 13818108 | 13880121 | -4.747836907 | 3.785577849 | 9.5E-14 | 4.45E-12 | 0.415472779 | 2.8252149 | Down |
| 2047 | ENSG00000154928 | K05110 | EPHB1 | chr3 | 134776089 | 135260467 | -4.136325594 | 3.326189908 | 2.33E-12 | 7.84E-11 | 0.490853659 | 2.928861789 | Down |
| 388272 | ENSG00000155330 | - | C16orf87 |  |  |  | -4.136314134 | 3.326189908 | 2.36E-12 | 7.94E-11 | 0.391304348 | 2.639751553 | Down |
| 285172 | ENSG00000155744 | - | FAM126B | chr2 | 200973718 | 201071671 | -5.131644918 | 4.096541259 | 1.02E-14 | 9.36E-13 | 0.509433962 | 2.849056604 | Down |
| 1193 | ENSG00000155962 | K05022 | CLIC2 |  |  |  | -3.262419385 | 2.765916931 | 0.0000011 | 0.0000237 | 0.530364372 | 3.194331984 | Down |
| 10 | ENSG00000156006 | K00622 | NAT2 | chr8 | 18386585 | 18401219 | -3.517020621 | 4.026762593 | 0.0000475 | 0.000698074 | 0.520689655 | 3.217241379 | Down |
| 157657 | ENSG00000156172 | - | C8orf37 | chr8 | 95244913 | 95270602 | -2.961991961 | 3.698629645 | 0.000503777 | 0.00591586 | 0.536231884 | 2.874396135 | Down |
| 10563 | ENSG00000156234 | K10032 | CXCL13 | chr4 | 77511753 | 77611834 | -3.262414213 | 2.765916931 | 0.00000111 | 0.0000237 | 0.348623853 | 3.220183486 | Down |
| 7102 | ENSG00000156298 | K06571 | TSPAN7 |  |  |  | -4.136303417 | 3.326189908 | 2.4E-12 | 8.03E-11 | 0.394736842 | 2.95112782 | Down |
| 23552 | ENSG00000156345 | K08817 | CDK20 | chr9 | 87966441 | 87974780 | -5.131641806 | 4.096541259 | 1.03E-14 | 9.36E-13 | 0.39017341 | 3.104046243 | Down |
| 122416 | ENSG00000156381 | - | ANKRD9 |  |  |  | -3.095614616 | 2.674084864 | 0.00000219 | 0.0000401 | 0.331230284 | 2.804416404 | Down |
| 60685 | ENSG00000156639 | - | ZFAND3 | chr6 | 37819496 | 38154624 | -5.131638372 | 4.096541259 | 1.04E-14 | 9.36E-13 | 0.577092511 | 2.599118943 | Down |
| 6750 | ENSG00000157005 | K05237 | SST |  |  |  | -5.131633555 | 4.096541259 | 1.05E-14 | 9.38E-13 | 0.474137931 | 2.75 | Down |
| 51738 | ENSG00000157017 | K05254 | GHRL |  |  |  | -5.131628741 | 4.096541259 | 1.06E-14 | 9.4E-13 | 0.452991453 | 2.897435897 | Down |
| 9372 | ENSG00000157077 | K04679 | ZFYVE9 | chr1 | 52142001 | 52348664 | -4.136292714 | 3.326189908 | 2.43E-12 | 8.12E-11 | 0.544561404 | 2.757894737 | Down |
| 134285 | ENSG00000157111 | - | TMEM171 | chr5 | 73120561 | 73131817 | -3.095360016 | 4.240277523 | 0.000508725 | 0.00591586 | 0.435185185 | 2.777777778 | Down |
| 203328 | ENSG00000157303 | - | SUSD3 |  |  |  | -4.136282023 | 3.326189908 | 2.47E-12 | 8.22E-11 | 0.345098039 | 2.623529412 | Down |
| 10901 | ENSG00000157326 | K11147 | DHRS4 | chr14 | 23953735 | 23969279 | -5.131623931 | 4.096541259 | 1.07E-14 | 9.42E-13 | 0.40647482 | 2.633093525 | Down |
| 5192 | ENSG00000157911 | K13346 | PEX10 |  |  |  | -3.898052213 | 5.32578119 | 0.000129088 | 0.001747494 | 0.343930636 | 3.046242775 | Down |
| 26100 | ENSG00000157954 | K17908 | WIPI2 | chr7 | 5190204 | 5233855 | -4.136270123 | 3.326189908 | 2.51E-12 | 8.33E-11 | 0.471365639 | 2.792951542 | Down |
| 138639 | ENSG00000158079 | K18078 | PTPDC1 | chr9 | 94030591 | 94109856 | -5.131619123 | 4.096541259 | 1.08E-14 | 9.44E-13 | 0.457920792 | 2.908415842 | Down |
| 195827 | ENSG00000158122 | - | AAED1 | chr9 | 96641251 | 96655331 | -5.683909375 | 4.568749497 | 1.86E-15 | 3.58E-13 | 0.380530973 | 2.933628319 | Down |
| 129531 | ENSG00000158411 | - | MITD1 | chr2 | 99161427 | 99181073 | -3.517023149 | 4.026762593 | 0.0000476 | 0.000698191 | 0.554216867 | 3.208835341 | Down |
| 340348 | ENSG00000158457 | K17346 | TSPAN33 | chr7 | 129144716 | 129169694 | -3.262409045 | 2.765916931 | 0.00000111 | 0.0000238 | 0.371024735 | 3.056537102 | Down |
| 23382 | ENSG00000158467 | K01251 | AHCYL2 |  |  |  | -6.128869412 | 4.964945407 | 5.96E-17 | 1.92E-14 | 0.464811784 | 2.765957447 | Down |
| 912 | ENSG00000158473 | K06448 | CD1D |  |  |  | -4.136262799 | 3.326189908 | 2.53E-12 | 8.39E-11 | 0.426865672 | 3.080597015 | Down |
| 1780 | ENSG00000158560 | K10415 | DYNC1I1 |  |  |  | -4.136255481 | 3.326189908 | 2.56E-12 | 8.44E-11 | 0.575193798 | 2.945736434 | Down |
| 56833 | ENSG00000158714 | K16853 | SLAMF8 |  |  |  | -3.09560878 | 2.674084864 | 0.0000022 | 0.0000401 | 0.421052632 | 3.003508772 | Down |
| 142683 | ENSG00000158764 | K17527 | ITLN2 | chr1 | 160945020 | 160957378 | -4.136249102 | 3.326189908 | 2.58E-12 | 8.49E-11 | 0.455384615 | 2.916923077 | Down |
| 10361 | ENSG00000158806 | K11277 | NPM2 | chr8 | 22023308 | 22036908 | -3.62468213 | 4.425833018 | 0.0000528 | 0.000758317 | 0.61682243 | 2.869158879 | Down |
| 8867 | ENSG00000159082 | K20279 | SYNJ1 | chr21 | 32628759 | 32728128 | -4.747832254 | 3.785577849 | 9.58E-14 | 4.46E-12 | 0.479528536 | 2.863523573 | Down |
| 714 | ENSG00000159189 | K03988 | C1QC | chr1 | 22643625 | 22648110 | -4.136242728 | 3.326189908 | 2.6E-12 | 8.54E-11 | 0.346938776 | 2.751020408 | Down |
| 9992 | ENSG00000159197 | K04896 | KCNE2 |  |  |  | -5.131614319 | 4.096541259 | 1.09E-14 | 9.46E-13 | 0.552845528 | 3.300813008 | Down |
| 201176 | ENSG00000159314 | K20636 | ARHGAP27 |  |  |  | -2.961994181 | 3.698629645 | 0.000504218 | 0.00591586 | 0.50056243 | 2.880764904 | Down |
| 221188 | ENSG00000159618 | K08459 | ADGRG5 |  |  |  | -4.136227885 | 3.326189908 | 2.65E-12 | 8.69E-11 | 0.380681818 | 3.064393939 | Down |
| 146206 | ENSG00000159753 | K20493 | RLTPR | chr16 | 67645004 | 67657569 | -4.064692331 | 3.280835385 | 6.11E-09 | 0.00000016 | 0.449477352 | 2.672473868 | Down |
| 7032 | ENSG00000160181 | - | TFF2 |  |  |  | -3.47507926 | 5.458570407 | 0.000507283 | 0.00591586 | 0.434108527 | 2.76744186 | Down |
| 7031 | ENSG00000160182 | - | TFF1 |  |  |  | -6.128866874 | 4.964945407 | 6E-17 | 1.92E-14 | 0.476190476 | 2.678571429 | Down |
| 5316 | ENSG00000160199 | - | PKNOX1 | chr21 | 42974510 | 43033931 | -4.136219726 | 3.326189908 | 2.68E-12 | 8.76E-11 | 0.555045872 | 2.72706422 | Down |
| 105372824 | ENSG00000160209 | - | LOC105372824 | chr21 | 43737000 | 43738097 | -3.963216555 | 4.325612745 | 0.00000717 | 0.000118229 | 0.483974359 | 2.958333333 | Down |
| 23181 | ENSG00000160305 | - | DIP2A |  |  |  | -3.262401861 | 2.765916931 | 0.00000112 | 0.0000239 | 0.419478039 | 2.805856143 | Down |
| 11093 | ENSG00000160323 | K08627 | ADAMTS13 | chr9 | 133414339 | 133459403 | -2.961996399 | 3.698629645 | 0.00050466 | 0.00591586 | 0.407848633 | 2.633496847 | Down |
| 26086 | ENSG00000160360 | K15839 | GPSM1 | chr9 | 136327476 | 136359605 | -3.190827495 | 4.579030301 | 0.000452896 | 0.005495138 | 0.496296296 | 2.785185185 | Down |
| 84446 | ENSG00000160469 | K08796 | BRSK1 | chr19 | 55284101 | 55312562 | -4.136208002 | 3.326189908 | 2.72E-12 | 8.87E-11 | 0.453400504 | 2.785894207 | Down |
| 84814 | ENSG00000160539 | - | PLPP7 |  |  |  | -5.13161025 | 4.096541259 | 1.1E-14 | 9.47E-13 | 0.324723247 | 2.826568266 | Down |
| 120425 | ENSG00000160593 | - | JAML | chr11 | 118193727 | 118225094 | -4.136202996 | 3.326189908 | 2.74E-12 | 8.91E-11 | 0.454314721 | 3.032994924 | Down |
| 55585 | ENSG00000160714 | K10582 | UBE2Q1 | chr1 | 154548575 | 154558644 | -5.683908777 | 4.568749497 | 1.86E-15 | 3.58E-13 | 0.509478673 | 2.803317536 | Down |
| 23218 | ENSG00000160796 | - | NBEAL2 | chr3 | 46979683 | 47009704 | -4.238365867 | 3.40286445 | 5.63E-09 | 0.000000153 | 0.433551198 | 2.893246187 | Down |
| 10898 | ENSG00000160917 | K14404 | CPSF4 | chr7 | 99438920 | 99457377 | -5.131606184 | 4.096541259 | 1.11E-14 | 9.48E-13 | 0.416356877 | 2.918215613 | Down |
| 126402 | ENSG00000160994 | - | CCDC105 | chr19 | 15010727 | 15023271 | -2.961998617 | 3.698629645 | 0.000505102 | 0.00591586 | 0.434869739 | 2.957915832 | Down |
| 11282 | ENSG00000161013 | K00738 | MGAT4B |  |  |  | -5.131602119 | 4.096541259 | 1.12E-14 | 9.49E-13 | 0.470692718 | 3.188277087 | Down |
| 91010 | ENSG00000161791 | - | FMNL3 | chr12 | 49636499 | 49707414 | -4.238361537 | 3.40286445 | 5.67E-09 | 0.000000153 | 0.489776047 | 3.05842259 | Down |
| 115950 | ENSG00000161914 | - | ZNF653 | chr19 | 11483427 | 11505923 | -3.262396546 | 2.765916931 | 0.00000112 | 0.0000239 | 0.474796748 | 2.718699187 | Down |
| 400569 | ENSG00000161920 | K15131 | MED11 |  |  |  | -4.238357209 | 3.40286445 | 5.7E-09 | 0.000000153 | 0.564102564 | 2.794871795 | Down |
| 51005 | ENSG00000162066 | K01443 | AMDHD2 | chr16 | 2520351 | 2531414 | -4.688245902 | 3.739046202 | 6.41E-14 | 3.57E-12 | 0.348484848 | 2.745791246 | Down |
| 10825 | ENSG00000162139 | K12357 | NEU3 |  |  |  | -3.262391235 | 2.765916931 | 0.00000113 | 0.000024 | 0.459869848 | 2.917570499 | Down |
| 249 | ENSG00000162551 | K01077 | ALPL | chr1 | 21508982 | 21578412 | -4.13619398 | 3.326189908 | 2.78E-12 | 8.99E-11 | 0.467557252 | 2.826335878 | Down |
| 6339 | ENSG00000162572 | K04826 | SCNN1D | chr1 | 1280415 | 1292029 | -4.688239843 | 3.739046202 | 6.48E-14 | 3.59E-12 | 0.382316314 | 2.826899128 | Down |
| 149233 | ENSG00000162594 | K05065 | IL23R |  |  |  | -4.136184973 | 3.326189908 | 2.81E-12 | 9.08E-11 | 0.548489666 | 3.093799682 | Down |
| 51375 | ENSG00000162627 | K17921 | SNX7 | chr1 | 98661344 | 98760500 | -3.624683464 | 4.425833018 | 0.0000529 | 0.00075835 | 0.585365854 | 3.042128603 | Down |
| 2634 | ENSG00000162645 | K20897 | GBP2 | chr1 | 89106132 | 89126159 | -5.683908178 | 4.568749497 | 1.87E-15 | 3.58E-13 | 0.541455161 | 3.037225042 | Down |
| 25903 | ENSG00000162745 | - | OLFML2B | chr1 | 161983192 | 162024465 | -4.064688244 | 3.280835385 | 6.14E-09 | 0.00000016 | 0.559254328 | 2.910785619 | Down |
| 148304 | ENSG00000162757 | - | C1orf74 |  |  |  | -5.131597433 | 4.096541259 | 1.13E-14 | 9.51E-13 | 0.423791822 | 2.944237918 | Down |
| 91461 | ENSG00000162878 | K17548 | PKDCC |  |  |  | -4.688233789 | 3.739046202 | 6.54E-14 | 3.61E-12 | 0.403651116 | 2.855983773 | Down |
| 23498 | ENSG00000162882 | K00452 | HAAO |  |  |  | -4.136176389 | 3.326189908 | 2.84E-12 | 9.16E-11 | 0.48951049 | 3.062937063 | Down |
| 4760 | ENSG00000162992 | K08033 | NEUROD1 | chr2 | 181676106 | 181680665 | -3.179035925 | 4.148862116 | 0.000343604 | 0.004281642 | 0.573033708 | 2.884831461 | Down |
| 90957 | ENSG00000163214 | K13026 | DHX57 | chr2 | 38797729 | 38875883 | -3.399134463 | 4.125804243 | 0.000126249 | 0.001713103 | 0.5 | 2.954545455 | Down |
| 388701 | ENSG00000163263 | - | C1orf189 | chr1 | 154199085 | 154206365 | -5.744124073 | 4.621282224 | 1.58E-15 | 3.55E-13 | 0.465346535 | 3.257425743 | Down |
| 152519 | ENSG00000163293 | - | NIPAL1 |  |  |  | -3.095601288 | 2.674084864 | 0.00000221 | 0.0000403 | 0.414634146 | 3.002439024 | Down |
| 151556 | ENSG00000163328 | - | GPR155 | chr2 | 174431571 | 174494001 | -4.518269417 | 4.737240222 | 0.000000583 | 0.0000137 | 0.412643678 | 3.098850575 | Down |
| 1293 | ENSG00000163359 | K06238 | COL6A3 |  |  |  | -3.095593803 | 2.674084864 | 0.00000222 | 0.0000404 | 0.457979226 | 2.794774945 | Down |
| 11167 | ENSG00000163430 | - | FSTL1 | chr3 | 120394214 | 120451071 | -4.688227739 | 3.739046202 | 6.61E-14 | 3.63E-12 | 0.558441558 | 2.892857143 | Down |
| 3577 | ENSG00000163464 | K04175 | CXCR1 |  |  |  | -3.095586325 | 2.674084864 | 0.00000223 | 0.0000405 | 0.348571429 | 3.24 | Down |
| 131474 | ENSG00000163528 | K17782 | CHCHD4 | chr3 | 14112077 | 14124871 | -3.179037012 | 4.148862116 | 0.000343958 | 0.004281642 | 0.696774194 | 2.793548387 | Down |
| 23429 | ENSG00000163602 | K11469 | RYBP | chr3 | 72374593 | 72446623 | -3.095576942 | 2.674084864 | 0.00000224 | 0.0000407 | 0.479495268 | 2.58044164 | Down |
| 64419 | ENSG00000163719 | K18086 | MTMR14 | chr3 | 9649433 | 9702394 | -5.131594035 | 4.096541259 | 1.14E-14 | 9.51E-13 | 0.501538462 | 2.915384615 | Down |
| 25898 | ENSG00000163743 | K10144 | RCHY1 | chr4 | 75479037 | 75515057 | -4.964027017 | 5.094673907 | 5.41E-08 | 0.00000136 | 0.501915709 | 2.858237548 | Down |
| 1359 | ENSG00000163751 | K08780 | CPA3 | chr3 | 148865256 | 148897091 | -3.262385929 | 2.765916931 | 0.00000113 | 0.000024 | 0.494004796 | 3.256594724 | Down |
| 116441 | ENSG00000163762 | - | TM4SF18 | chr3 | 149318498 | 149333761 | -4.136167814 | 3.326189908 | 2.88E-12 | 9.24E-11 | 0.373134328 | 3.074626866 | Down |
| 54585 | ENSG00000163818 | K19400 | LZTFL1 |  |  |  | -4.136159248 | 3.326189908 | 2.91E-12 | 9.32E-11 | 0.628762542 | 3.063545151 | Down |
| 79442 | ENSG00000163827 | - | LRRC2 | chr3 | 46515385 | 46580099 | -4.747827522 | 3.785577849 | 9.66E-14 | 4.48E-12 | 0.53638814 | 3.15902965 | Down |
| 7802 | ENSG00000163879 | K10410 | DNALI1 |  |  |  | -3.095570549 | 2.674084864 | 0.00000225 | 0.0000408 | 0.528571429 | 2.921428571 | Down |
| 28999 | ENSG00000163884 | K09210 | KLF15 | chr3 | 126288121 | 126357393 | -3.784402026 | 4.376575437 | 0.0000221 | 0.000343343 | 0.396634615 | 2.622596154 | Down |
| 90407 | ENSG00000163900 | - | TMEM41A | chr3 | 185482671 | 185499057 | -6.128864337 | 4.964945407 | 6.05E-17 | 1.92E-14 | 0.340909091 | 3.25 | Down |
| 57654 | ENSG00000163945 | - | UVSSA | chr4 | 1345662 | 1388049 | -5.13158832 | 4.096541259 | 1.15E-14 | 9.55E-13 | 0.543018336 | 2.909732017 | Down |
| 64850 | ENSG00000164089 | K14286 | ETNPPL | chr4 | 108742040 | 108763180 | -3.262380626 | 2.765916931 | 0.00000114 | 0.0000241 | 0.46492986 | 2.931863727 | Down |
| 9464 | ENSG00000164107 | K18486 | HAND2 | chr4 | 173526501 | 173530227 | -3.963219659 | 4.325612745 | 0.00000718 | 0.000118328 | 0.423963134 | 2.815668203 | Down |
| 51313 | ENSG00000164125 | - | FAM198B | chr4 | 158124580 | 158173050 | -4.13615069 | 3.326189908 | 2.94E-12 | 9.4E-11 | 0.373814042 | 2.90512334 | Down |
| 493869 | ENSG00000164294 | K00432 | GPX8 | chr5 | 55160118 | 55167301 | -4.784775859 | 5.124914931 | 0.000000167 | 0.00000407 | 0.397129187 | 3.301435407 | Down |
| 154743 | ENSG00000164603 | - | C7orf60 | chr7 | 112819147 | 112939877 | -3.262375328 | 2.765916931 | 0.00000114 | 0.0000242 | 0.516049383 | 3.091358025 | Down |
| 8645 | ENSG00000164626 | K04916 | KCNK5 |  |  |  | -3.095564161 | 2.674084864 | 0.00000226 | 0.0000409 | 0.45490982 | 3.006012024 | Down |
| 94120 | ENSG00000164674 | K17598 | SYTL3 | chr6 | 158644881 | 158766298 | -4.688221695 | 3.739046202 | 6.68E-14 | 3.65E-12 | 0.455737705 | 2.93442623 | Down |
| 202915 | ENSG00000164855 | - | TMEM184A | chr7 | 1542235 | 1560821 | -3.876473577 | 4.892966714 | 0.000147778 | 0.001972553 | 0.387409201 | 3.07748184 | Down |
| 138716 | ENSG00000164967 | K14525 | RPP25L | chr9 | 34610485 | 34612113 | -5.13158261 | 4.096541259 | 1.16E-14 | 9.6E-13 | 0.36809816 | 2.680981595 | Down |
| 216 | ENSG00000165092 | K07249 | ALDH1A1 | chr9 | 72900662 | 72953317 | -4.034986747 | 4.850010943 | 0.0000086 | 0.000139915 | 0.453093812 | 2.896207585 | Down |
| 57526 | ENSG00000165194 | K16499 | PCDH19 | chrX | 100291644 | 100410273 | -3.095557778 | 2.674084864 | 0.00000227 | 0.000041 | 0.548780488 | 2.804878049 | Down |
| 145581 | ENSG00000165379 | K16358 | LRFN5 |  |  |  | -3.262369512 | 2.765916931 | 0.00000115 | 0.0000242 | 0.496522949 | 2.847009736 | Down |
| 1073 | ENSG00000165410 | K05765 | CFL2 | chr14 | 34710382 | 34714823 | -3.095549336 | 2.674084864 | 0.00000228 | 0.0000412 | 0.530120482 | 3.048192771 | Down |
| 222389 | ENSG00000165626 | - | BEND7 |  |  |  | -4.136142141 | 3.326189908 | 2.98E-12 | 9.48E-11 | 0.482905983 | 2.758547009 | Down |
| 509 | ENSG00000165629 | K02136 | ATP5C1 | chr10 | 7788104 | 7807801 | -3.496466836 | 4.728879567 | 0.0003775 | 0.004664245 | 0.483221477 | 2.906040268 | Down |
| 10495 | ENSG00000165675 | - | ENOX2 |  |  |  | -3.262363702 | 2.765916931 | 0.00000116 | 0.0000243 | 0.532786885 | 3.024590164 | Down |
| 6297 | ENSG00000165821 | K19871 | SALL2 | chr14 | 21521080 | 21537216 | -4.136133601 | 3.326189908 | 3.01E-12 | 9.56E-11 | 0.474677259 | 2.514399206 | Down |
| 1557 | ENSG00000165841 | K17721 | CYP2C19 |  |  |  | -3.262357896 | 2.765916931 | 0.00000116 | 0.0000244 | 0.448979592 | 3.157142857 | Down |
| 145567 | ENSG00000165914 | - | TTC7B | chr14 | 90540588 | 90816479 | -5.131578711 | 4.096541259 | 1.17E-14 | 9.6E-13 | 0.472123369 | 2.941874259 | Down |
| 25841 | ENSG00000166016 | K10521 | ABTB2 | chr11 | 34150987 | 34358008 | -3.784403265 | 4.376575437 | 0.0000222 | 0.000343343 | 0.48195122 | 2.842926829 | Down |
| 161742 | ENSG00000166068 | K04703 | SPRED1 | chr15 | 38252087 | 38357249 | -5.131571978 | 4.096541259 | 1.19E-14 | 9.67E-13 | 0.554054054 | 2.862612613 | Down |
| 83700 | ENSG00000166086 | K06785 | JAM3 | chr11 | 134068925 | 134151757 | -3.095540904 | 2.674084864 | 0.00000229 | 0.0000413 | 0.483870968 | 2.983870968 | Down |
| 1152 | ENSG00000166165 | K00933 | CKB |  |  |  | -5.131568133 | 4.096541259 | 1.2E-14 | 9.68E-13 | 0.501312336 | 2.939632546 | Down |
| 1200 | ENSG00000166340 | K01279 | TPP1 | chr11 | 6612766 | 6619461 | -3.96322276 | 4.325612745 | 0.0000072 | 0.000118428 | 0.444049734 | 2.857904085 | Down |
| 7275 | ENSG00000166402 | - | TUB | chr11 | 8019180 | 8106107 | -3.1790381 | 4.148862116 | 0.000344313 | 0.004281642 | 0.511586453 | 2.762923351 | Down |
| 8509 | ENSG00000166507 | K02577 | NDST2 | chr10 | 73801911 | 73811831 | -3.645247792 | 5.401958111 | 0.000324466 | 0.004061495 | 0.429218573 | 3.200453001 | Down |
| 90701 | ENSG00000166562 | K13280 | SEC11C |  |  |  | -4.13612507 | 3.326189908 | 3.05E-12 | 9.65E-11 | 0.385416667 | 3.098958333 | Down |
| 6236 | ENSG00000166592 | K07845 | RRAD | chr16 | 66921679 | 66925536 | -5.131563289 | 4.096541259 | 1.21E-14 | 9.71E-13 | 0.451298701 | 2.616883117 | Down |
| 80063 | ENSG00000166669 | - | ATF7IP2 | chr16 | 10386055 | 10483640 | -3.23949213 | 4.184151988 | 0.00028244 | 0.003570862 | 0.582111437 | 2.774193548 | Down |
| 80095 | ENSG00000166704 | K09228 | ZNF606 | chr19 | 57977072 | 58003346 | -5.131558448 | 4.096541259 | 1.22E-14 | 9.74E-13 | 0.507575758 | 3.074494949 | Down |
| 84191 | ENSG00000166797 | - | FAM96A | chr15 | 64072559 | 64094008 | -4.784776077 | 5.124914931 | 0.000000168 | 0.00000407 | 0.55625 | 3.03125 | Down |
| 100287413 | ENSG00000166840 | - | LOC100287413 |  |  |  | -4.136117041 | 3.326189908 | 3.08E-12 | 9.73E-11 | 0.528528529 | 3.144144144 | Down |
| 54893 | ENSG00000166912 | K18085 | MTMR10 | chr15 | 30919764 | 30991604 | -4.13610902 | 3.326189908 | 3.11E-12 | 9.81E-11 | 0.474903475 | 3.108108108 | Down |
| 115752 | ENSG00000166938 | K18681 | DIS3L | chr15 | 66293257 | 66333898 | -4.747820188 | 3.785577849 | 9.79E-14 | 4.53E-12 | 0.521821632 | 3.060721063 | Down |
| 10982 | ENSG00000166974 | K10436 | MAPRE2 | chr18 | 34976928 | 35143470 | -5.13155361 | 4.096541259 | 1.24E-14 | 9.77E-13 | 0.571865443 | 2.972477064 | Down |
| 150365 | ENSG00000167077 | - | MEI1 | chr22 | 41699503 | 41799456 | -3.229793662 | 4.563637906 | 0.000897464 | 0.009774872 | 0.432496075 | 2.960753532 | Down |
| 5090 | ENSG00000167081 | K15610 | PBX3 | chr9 | 125747338 | 125967377 | -4.238352884 | 3.40286445 | 5.73E-09 | 0.000000154 | 0.525274725 | 2.63956044 | Down |
| 162461 | ENSG00000167105 | - | TMEM92 |  |  |  | -3.262352095 | 2.765916931 | 0.00000117 | 0.0000244 | 0.34591195 | 2.905660377 | Down |
| 2021 | ENSG00000167136 | K01173 | ENDOG | chr9 | 128818402 | 128822676 | -5.13154816 | 4.096541259 | 1.25E-14 | 9.81E-13 | 0.390572391 | 2.878787879 | Down |
| 56905 | ENSG00000167173 | - | C15orf39 |  |  |  | -4.238348562 | 3.40286445 | 5.77E-09 | 0.000000155 | 0.400191022 | 2.687679083 | Down |
| 2879 | ENSG00000167468 | K05361 | GPX4 | chr19 | 1103926 | 1106789 | -4.136101007 | 3.326189908 | 3.15E-12 | 9.88E-11 | 0.334710744 | 2.876033058 | Down |
| 147015 | ENSG00000167536 | K11169 | DHRS13 |  |  |  | -4.06468416 | 3.280835385 | 6.17E-09 | 0.000000161 | 0.389920424 | 2.779840849 | Down |
| 148170 | ENSG00000167617 | - | CDC42EP5 |  |  |  | -6.128861801 | 4.964945407 | 6.09E-17 | 1.92E-14 | 0.331081081 | 2.52027027 | Down |
| 126259 | ENSG00000167664 | K16668 | TMIGD2 | chr19 | 4292227 | 4302439 | -3.577573429 | 4.065055456 | 0.0000385 | 0.000575302 | 0.40070922 | 2.776595745 | Down |
| 84798 | ENSG00000167747 | - | C19orf48 |  |  |  | -3.262349209 | 2.765916931 | 0.00000117 | 0.0000245 | 0.384615385 | 2.931623932 | Down |
| 4723 | ENSG00000167792 | K03942 | NDUFV1 | chr11 | 67606852 | 67612541 | -4.049163239 | 5.284390257 | 0.0000313 | 0.00047399 | 0.413793103 | 2.827586207 | Down |
| 10871 | ENSG00000167850 | K06719 | CD300C | chr17 | 74534359 | 74546171 | -3.095532479 | 2.674084864 | 0.0000023 | 0.0000415 | 0.419642857 | 2.924107143 | Down |
| 11314 | ENSG00000167851 | K06719 | CD300A | chr17 | 74466320 | 74484798 | -3.480518347 | 4.47349386 | 0.000102018 | 0.001398393 | 0.484949833 | 2.956521739 | Down |
| 283987 | ENSG00000167861 | - | HID1 | chr17 | 74950742 | 74972805 | -3.229793913 | 4.563637906 | 0.000897606 | 0.009774872 | 0.472081218 | 3.08248731 | Down |
| 6730 | ENSG00000167881 | K03107 | SRP68 | chr17 | 76038775 | 76072526 | -3.229794054 | 4.563637906 | 0.000897686 | 0.009774872 | 0.502392344 | 2.974481659 | Down |
| 50964 | ENSG00000167941 | K16834 | SOST | chr17 | 43753731 | 43758788 | -3.784404504 | 4.376575437 | 0.0000222 | 0.000343343 | 0.403755869 | 2.915492958 | Down |
| 9881 | ENSG00000168016 | - | TRANK1 | chr3 | 36826817 | 36945662 | -2.962000834 | 3.698629645 | 0.000505544 | 0.00591586 | 0.502564103 | 3.122051282 | Down |
| 3800 | ENSG00000168280 | K10396 | KIF5C | chr2 | 148875223 | 149026759 | -2.962003051 | 3.698629645 | 0.000505986 | 0.00591586 | 0.603970742 | 2.938349007 | Down |
| 11170 | ENSG00000168309 | - | FAM107A |  |  |  | -4.688215882 | 3.739046202 | 6.75E-14 | 3.67E-12 | 0.514285714 | 3.148571429 | Down |
| 84879 | ENSG00000168389 | - | MFSD2A | chr1 | 39955112 | 39969968 | -3.784406152 | 4.376575437 | 0.0000222 | 0.000343421 | 0.395948435 | 3.097605893 | Down |
| 84203 | ENSG00000168454 | - | TXNDC2 |  |  |  | -3.09552347 | 2.674084864 | 0.00000232 | 0.0000417 | 0.6039783 | 2.783001808 | Down |
| 9796 | ENSG00000168490 | - | PHYHIP | chr8 | 22219703 | 22232338 | -3.229794377 | 4.563637906 | 0.000897869 | 0.009774872 | 0.493939394 | 3.057575758 | Down |
| 135656 | ENSG00000168631 | - | DPCR1 | chr6 | 30941000 | 30954221 | -3.784407799 | 4.376575437 | 0.0000223 | 0.0003435 | 0.679109835 | 2.567839196 | Down |
| 54910 | ENSG00000168758 | K06521 | SEMA4C | chr2 | 96859736 | 96870943 | -4.049163886 | 5.284390257 | 0.0000313 | 0.00047399 | 0.422569028 | 2.979591837 | Down |
| 79867 | ENSG00000168778 | K19361 | TCTN2 | chr12 | 123671108 | 123708405 | -4.136093001 | 3.326189908 | 3.18E-12 | 9.96E-11 | 0.467718795 | 2.913916786 | Down |
| 5865 | ENSG00000169213 | K06108 | RAB3B |  |  |  | -4.136085009 | 3.326189908 | 3.21E-12 | 1E-10 | 0.570776256 | 2.98173516 | Down |
| 204474 | ENSG00000169340 | K20354 | PDILT | chr16 | 20359170 | 20404742 | -3.262346325 | 2.765916931 | 0.00000117 | 0.0000245 | 0.494863014 | 3.179794521 | Down |
| 3576 | ENSG00000169429 | K10030 | CXCL8 |  |  |  | -5.131542052 | 4.096541259 | 1.27E-14 | 9.87E-13 | 0.383838384 | 3 | Down |
| 56287 | ENSG00000169605 | - | GKN1 |  |  |  | -5.131535972 | 4.096541259 | 1.28E-14 | 9.91E-13 | 0.48241206 | 2.874371859 | Down |
| 123207 | ENSG00000169609 | K09131 | C15orf40 | chr15 | 82988297 | 83012298 | -3.811390501 | 5.350646805 | 0.000259001 | 0.003301078 | 0.389221557 | 2.51497006 | Down |
| 5986 | ENSG00000169733 | K05948 | RFNG | chr17 | 82047902 | 82051774 | -3.277969272 | 5.529693432 | 0.000876386 | 0.009765445 | 0.383685801 | 2.86102719 | Down |
| 5881 | ENSG00000169750 | K07861 | RAC3 | chr17 | 82031656 | 82034204 | -3.480518725 | 4.47349386 | 0.000102081 | 0.001398393 | 0.421875 | 3.010416667 | Down |
| 9185 | ENSG00000169891 | K20068 | REPS2 | chrX | 16946691 | 17194274 | -4.136072488 | 3.326189908 | 3.27E-12 | 1.02E-10 | 0.490909091 | 2.746969697 | Down |
| 375484 | ENSG00000170085 | - | SIMC1 | chr5 | 176238359 | 176345991 | -5.131532395 | 4.096541259 | 1.29E-14 | 9.91E-13 | 0.492704826 | 2.858585859 | Down |
| 84140 | ENSG00000170264 | K16772 | FAM161A |  |  |  | -5.131528821 | 4.096541259 | 1.3E-14 | 9.91E-13 | 0.583798883 | 3.074022346 | Down |
| 2018 | ENSG00000170370 | K09317 | EMX2 |  |  |  | -3.095514471 | 2.674084864 | 0.00000233 | 0.0000418 | 0.424603175 | 3.027777778 | Down |
| 345275 | ENSG00000170509 | - | HSD17B13 | chr4 | 87303789 | 87322906 | -3.262342924 | 2.765916931 | 0.00000118 | 0.0000245 | 0.41 | 3.076666667 | Down |
| 79071 | ENSG00000170522 | K10203 | ELOVL6 | chr4 | 110049073 | 110199199 | -3.963225268 | 4.325612745 | 0.00000721 | 0.000118472 | 0.358490566 | 3.535849057 | Down |
| 1000 | ENSG00000170558 | K06736 | CDH2 | chr18 | 27932878 | 28177446 | -4.136062723 | 3.326189908 | 3.31E-12 | 1.03E-10 | 0.535320088 | 2.864238411 | Down |
| 6444 | ENSG00000170624 | K12563 | SGCD |  |  |  | -3.095510496 | 2.674084864 | 0.00000234 | 0.0000418 | 0.424137931 | 2.944827586 | Down |
| 79145 | ENSG00000170791 | - | CHCHD7 | chr8 | 56211638 | 56218798 | -3.784409234 | 4.376575437 | 0.0000223 | 0.000343509 | 0.509090909 | 3.072727273 | Down |
| 1056 | ENSG00000170835 | K12298 | CEL |  |  |  | -4.238344243 | 3.40286445 | 5.8E-09 | 0.000000155 | 0.431216931 | 2.724867725 | Down |
| 8493 | ENSG00000170836 | K10147 | PPM1D | chr17 | 60600183 | 60666280 | -3.963227205 | 4.325612745 | 0.00000722 | 0.000118472 | 0.452892562 | 2.788429752 | Down |
| 84437 | ENSG00000170903 | - | MSANTD4 |  |  |  | -5.131525248 | 4.096541259 | 1.31E-14 | 9.91E-13 | 0.64057971 | 3.182608696 | Down |
| 5581 | ENSG00000171132 | K18050 | PRKCE |  |  |  | -3.262336449 | 2.765916931 | 0.00000118 | 0.0000246 | 0.474898236 | 3.023066486 | Down |
| 2548 | ENSG00000171298 | K12316 | GAA | chr17 | 80101526 | 80119882 | -4.688210075 | 3.739046202 | 6.82E-14 | 3.69E-12 | 0.43697479 | 2.974789916 | Down |
| 5223 | ENSG00000171314 | K01834 | PGAM1 | chr10 | 97426125 | 97433444 | -5.131521678 | 4.096541259 | 1.32E-14 | 9.91E-13 | 0.464566929 | 3.047244094 | Down |
| 9 | ENSG00000171428 | K00622 | NAT1 | chr8 | 18170419 | 18223689 | -3.963229141 | 4.325612745 | 0.00000723 | 0.000118472 | 0.508522727 | 3.232954545 | Down |
| 54811 | ENSG00000171466 | K09228 | ZNF562 |  |  |  | -4.688204272 | 3.739046202 | 6.89E-14 | 3.71E-12 | 0.495305164 | 2.978873239 | Down |
| 93134 | ENSG00000171469 | K09228 | ZNF561 |  |  |  | -4.688198473 | 3.739046202 | 6.96E-14 | 3.73E-12 | 0.473251029 | 2.977366255 | Down |
| 5481 | ENSG00000171497 | K05864 | PPID | chr4 | 158709127 | 158723400 | -3.2297947 | 4.563637906 | 0.000898052 | 0.009774872 | 0.464864865 | 2.897297297 | Down |
| 2186 | ENSG00000171634 | K11728 | BPTF | chr17 | 67825517 | 67984378 | -3.338474241 | 4.089072688 | 0.000136617 | 0.001828784 | 0.590282337 | 2.772816809 | Down |
| 79029 | ENSG00000171763 | - | SPATA5L1 | chr15 | 45400202 | 45421419 | -3.2394933 | 4.184151988 | 0.00028276 | 0.003570862 | 0.395750332 | 2.735723772 | Down |
| 92342 | ENSG00000171806 | - | METTL18 | chr1 | 169792524 | 169794976 | -3.784410942 | 4.376575437 | 0.0000223 | 0.000343609 | 0.516129032 | 3.091397849 | Down |
| 361 | ENSG00000171885 | K09866 | AQP4 |  |  |  | -5.13151731 | 4.096541259 | 1.33E-14 | 9.91E-13 | 0.352941176 | 2.879256966 | Down |
| 57834 | ENSG00000171903 | K17729 | CYP4F11 | chr19 | 15912370 | 15934866 | -2.962005266 | 3.698629645 | 0.000506428 | 0.00591586 | 0.429389313 | 3.213740458 | Down |
| 11346 | ENSG00000171992 | K21112 | SYNPO |  |  |  | -3.262329979 | 2.765916931 | 0.00000119 | 0.0000247 | 0.482238967 | 2.640473628 | Down |
| 5068 | ENSG00000172016 | - | REG3A |  |  |  | -3.262323516 | 2.765916931 | 0.00000119 | 0.0000248 | 0.457142857 | 2.885714286 | Down |
| 51315 | ENSG00000172086 | - | KRCC1 | chr2 | 88027203 | 88055801 | -3.624684658 | 4.425833018 | 0.000053 | 0.00075835 | 0.602316602 | 3.162162162 | Down |
| 257019 | ENSG00000172159 | - | FRMD3 | chr9 | 83242990 | 83560208 | -3.963231076 | 4.325612745 | 0.00000724 | 0.000118472 | 0.507537688 | 3.195979899 | Down |
| 114900 | ENSG00000172247 | - | C1QTNF4 | chr11 | 47589664 | 47596336 | -5.131510169 | 4.096541259 | 1.35E-14 | 1E-12 | 0.386018237 | 2.787234043 | Down |
| 257194 | ENSG00000172260 | K06775 | NEGR1 |  |  |  | -5.131503036 | 4.096541259 | 1.37E-14 | 1.01E-12 | 0.471751412 | 2.836158192 | Down |
| 199857 | ENSG00000172339 | K07441 | ALG14 |  |  |  | -3.095506522 | 2.674084864 | 0.00000234 | 0.0000419 | 0.37037037 | 3.083333333 | Down |
| 9854 | ENSG00000172375 | - | C2CD2L | chr11 | 119107061 | 119118543 | -4.136052969 | 3.326189908 | 3.36E-12 | 1.04E-10 | 0.480905233 | 2.678925035 | Down |
| 9915 | ENSG00000172379 | K15589 | ARNT2 | chr15 | 80404350 | 80597937 | -4.136043227 | 3.326189908 | 3.4E-12 | 1.05E-10 | 0.511854951 | 2.740585774 | Down |
| 55970 | ENSG00000172380 | K04347 | GNG12 | chr1 | 67701466 | 67833779 | -3.78441265 | 4.376575437 | 0.0000224 | 0.000343709 | 0.513888889 | 2.763888889 | Down |
| 150678 | ENSG00000172428 | - | MYEOV2 | chr2 | 240126548 | 240136802 | -4.747814459 | 3.785577849 | 9.89E-14 | 4.55E-12 | 0.523809524 | 2.646825397 | Down |
| 30008 | ENSG00000172638 | K19866 | EFEMP2 | chr11 | 65866441 | 65872934 | -3.095500734 | 2.674084864 | 0.00000235 | 0.0000419 | 0.516930023 | 2.76523702 | Down |
| 80194 | ENSG00000172663 | - | TMEM134 | chr11 | 67464348 | 67469277 | -5.131498948 | 4.096541259 | 1.38E-14 | 1.01E-12 | 0.456410256 | 2.984615385 | Down |
| 5017 | ENSG00000172818 | K09216 | OVOL1 | chr11 | 65787034 | 65797219 | -3.09549495 | 2.674084864 | 0.00000236 | 0.000042 | 0.438202247 | 2.928838951 | Down |
| 116535 | ENSG00000172935 | K08394 | MRGPRF | chr11 | 69004375 | 69013382 | -4.136033496 | 3.326189908 | 3.45E-12 | 1.06E-10 | 0.279883382 | 3.104956268 | Down |
| 49854 | ENSG00000173276 | - | ZBTB21 | chr21 | 41986831 | 42010418 | -4.578849282 | 4.784107766 | 0.000000468 | 0.0000111 | 0.512195122 | 2.877110694 | Down |
| 1605 | ENSG00000173402 | K06265 | DAG1 | chr3 | 49468703 | 49535618 | -4.136023777 | 3.326189908 | 3.49E-12 | 1.07E-10 | 0.449162011 | 2.829050279 | Down |
| 2286 | ENSG00000173486 | K09569 | FKBP2 | chr11 | 64240941 | 64244135 | -6.128859266 | 4.964945407 | 6.14E-17 | 1.92E-14 | 0.394366197 | 2.908450704 | Down |
| 5091 | ENSG00000173599 | K01958 | PC | chr11 | 66848522 | 66958418 | -4.229849072 | 5.238995557 | 0.0000148 | 0.000239158 | 0.433786078 | 2.893039049 | Down |
| 64802 | ENSG00000173614 | K06210 | NMNAT1 | chr1 | 9942923 | 9996739 | -2.962007481 | 3.698629645 | 0.000506871 | 0.00591586 | 0.512544803 | 3.046594982 | Down |
| 27129 | ENSG00000173641 | K09546 | HSPB7 |  |  |  | -5.131492314 | 4.096541259 | 1.4E-14 | 1.02E-12 | 0.46122449 | 2.824489796 | Down |
| 6777 | ENSG00000173757 | K11224 | STAT5B | chr17 | 42199177 | 42276460 | -5.131485686 | 4.096541259 | 1.42E-14 | 1.03E-12 | 0.520965693 | 3.062261753 | Down |
| 10276 | ENSG00000173848 | K20683 | NET1 |  |  |  | -3.624685617 | 4.425833018 | 0.0000531 | 0.00075835 | 0.503355705 | 2.968120805 | Down |
| 26996 | ENSG00000173890 | K08438 | GPR160 | chr3 | 170037323 | 170085415 | -3.383172606 | 5.051423819 | 0.000513969 | 0.005942872 | 0.369822485 | 3.594674556 | Down |
| 345757 | ENSG00000174132 | - | FAM174A | chr5 | 100535305 | 100586741 | -4.964026586 | 5.094673907 | 5.41E-08 | 0.00000136 | 0.405263158 | 2.526315789 | Down |
| 488 | ENSG00000174437 | K05853 | ATP2A2 | chr12 | 110281227 | 110351093 | -3.262317058 | 2.765916931 | 0.0000012 | 0.0000248 | 0.454894434 | 2.899232246 | Down |
| 400073 | ENSG00000174456 | - | C12orf76 | chr12 | 110041177 | 110067695 | -4.624827565 | 5.154542875 | 0.00000041 | 0.00000981 | 0.392592593 | 2.837037037 | Down |
| 115019 | ENSG00000174502 | K14706 | SLC26A9 |  |  |  | -6.128857368 | 4.964945407 | 6.17E-17 | 1.92E-14 | 0.405862458 | 2.988726043 | Down |
| 55554 | ENSG00000174562 | K09623 | KLK15 |  |  |  | -5.396568531 | 4.320582808 | 1.33E-14 | 9.91E-13 | 0.4140625 | 2.73046875 | Down |
| 1240 | ENSG00000174600 | K04245 | CMKLR1 |  |  |  | -4.136014069 | 3.326189908 | 3.54E-12 | 1.08E-10 | 0.378016086 | 3.193029491 | Down |
| 90806 | ENSG00000174606 | K18729 | ANGEL2 | chr1 | 212992182 | 213015875 | -4.747806261 | 3.785577849 | 1E-13 | 4.6E-12 | 0.474264706 | 3.088235294 | Down |
| 10781 | ENSG00000174652 | - | ZNF266 | chr19 | 9412426 | 9435578 | -3.963233137 | 4.325612745 | 0.00000725 | 0.000118482 | 0.491803279 | 2.870673953 | Down |
| 3952 | ENSG00000174697 | K05424 | LEP | chr7 | 128241201 | 128257629 | -3.095487261 | 2.674084864 | 0.00000237 | 0.0000422 | 0.461077844 | 3.011976048 | Down |
| 57124 | ENSG00000174807 | K06706 | CD248 |  |  |  | -4.688192679 | 3.739046202 | 7.03E-14 | 3.75E-12 | 0.424042272 | 2.673712021 | Down |
| 2015 | ENSG00000174837 | K04591 | ADGRE1 | chr19 | 6887549 | 6940453 | -4.136004373 | 3.326189908 | 3.58E-12 | 1.1E-10 | 0.459367946 | 2.83972912 | Down |
| 10897 | ENSG00000174851 | K20362 | YIF1A | chr11 | 66284580 | 66289167 | -3.624686183 | 4.425833018 | 0.0000531 | 0.00075835 | 0.351535836 | 3.040955631 | Down |
| 253982 | ENSG00000174939 | - | ASPHD1 | chr16 | 29900392 | 29919860 | -5.131479065 | 4.096541259 | 1.44E-14 | 1.03E-12 | 0.330769231 | 2.633333333 | Down |
| 285598 | ENSG00000175414 | K07958 | ARL10 | chr5 | 176365468 | 176415085 | -5.131472449 | 4.096541259 | 1.46E-14 | 1.04E-12 | 0.475409836 | 3.090163934 | Down |
| 80227 | ENSG00000175575 | K11887 | PAAF1 | chr11 | 73876699 | 73927736 | -3.624687483 | 4.425833018 | 0.0000532 | 0.000758631 | 0.458015267 | 2.763358779 | Down |
| 146691 | ENSG00000175662 | - | TOM1L2 | chr17 | 17843508 | 17972470 | -3.963236316 | 4.325612745 | 0.00000727 | 0.00011859 | 0.554240631 | 2.735700197 | Down |
| 2 | ENSG00000175899 | K03910 | A2M | chr12 | 9067708 | 9115962 | -3.262310607 | 2.765916931 | 0.00000121 | 0.0000249 | 0.495929444 | 2.95183175 | Down |
| 163259 | ENSG00000175984 | K20161 | DENND2C |  |  |  | -4.135993608 | 3.326189908 | 3.64E-12 | 1.11E-10 | 0.516163793 | 3.120689655 | Down |
| 3291 | ENSG00000176387 | K00071 | HSD11B2 |  |  |  | -4.23834139 | 3.40286445 | 5.83E-09 | 0.000000155 | 0.338271605 | 2.901234568 | Down |
| 205717 | ENSG00000176542 | - | USF3 | chr3 | 113648385 | 113696657 | -4.064679872 | 3.280835385 | 6.21E-09 | 0.000000161 | 0.536302895 | 2.579955457 | Down |
| 84002 | ENSG00000176597 | K03766 | B3GNT5 | chr3 | 183253207 | 183273391 | -3.262304162 | 2.765916931 | 0.00000121 | 0.000025 | 0.473544974 | 3.298941799 | Down |
| 51320 | ENSG00000176624 | K15686 | MEX3C | chr18 | 51174550 | 51197681 | -3.229795378 | 4.563637906 | 0.000898436 | 0.009774872 | 0.481031866 | 2.558421851 | Down |
| 10409 | ENSG00000176788 | K17272 | BASP1 | chr5 | 17216823 | 17276845 | -4.688183668 | 3.739046202 | 7.14E-14 | 3.79E-12 | 0.607929515 | 2.211453744 | Down |
| 284207 | ENSG00000176845 | - | METRNL |  |  |  | -2.962009695 | 3.698629645 | 0.000507314 | 0.00591586 | 0.395498392 | 2.84244373 | Down |
| 54503 | ENSG00000177054 | K20032 | ZDHHC13 | chr11 | 19117099 | 19176420 | -3.452529221 | 5.027038536 | 0.000726923 | 0.008293428 | 0.377813505 | 3.239549839 | Down |
| 150368 | ENSG00000177096 | - | FAM109B | chr22 | 42074244 | 42079438 | -3.262298542 | 2.765916931 | 0.00000122 | 0.0000251 | 0.432432432 | 2.783783784 | Down |
| 125228 | ENSG00000177150 | - | FAM210A |  |  |  | -5.131466412 | 4.096541259 | 1.48E-14 | 1.05E-12 | 0.433823529 | 3.091911765 | Down |
| 64925 | ENSG00000177352 | - | CCDC71 | chr3 | 49162535 | 49166352 | -4.747800765 | 3.785577849 | 1.01E-13 | 4.63E-12 | 0.29764454 | 2.640256959 | Down |
| 125170 | ENSG00000177427 | - | MIEF2 | chr17 | 18260534 | 18265790 | -3.624688672 | 4.425833018 | 0.0000533 | 0.000758809 | 0.417204301 | 2.843010753 | Down |
| 122970 | ENSG00000177465 | K01068 | ACOT4 | chr14 | 73591528 | 73595766 | -3.095481461 | 2.674084864 | 0.00000238 | 0.0000423 | 0.365795724 | 2.966745843 | Down |
| 167826 | ENSG00000177468 | K09085 | OLIG3 | chr6 | 137492199 | 137494394 | -2.962011909 | 3.698629645 | 0.000507757 | 0.00591586 | 0.397058824 | 2.647058824 | Down |
| 10949 | ENSG00000177733 | K12894 | HNRNPA0 | chr5 | 137751384 | 137754350 | -4.578849264 | 4.784107766 | 0.000000468 | 0.0000111 | 0.403278689 | 2.367213115 | Down |
| 158056 | ENSG00000177943 | - | MAMDC4 |  |  |  | -4.135983875 | 3.326189908 | 3.68E-12 | 1.12E-10 | 0.428453947 | 2.737664474 | Down |
| 3615 | ENSG00000178035 | K00088 | IMPDH2 | chr3 | 49024325 | 49029750 | -4.229849879 | 5.238995557 | 0.0000148 | 0.000239158 | 0.461089494 | 2.79766537 | Down |
| 3350 | ENSG00000178394 | K04153 | HTR1A |  |  |  | -5.13146038 | 4.096541259 | 1.5E-14 | 1.05E-12 | 0.369668246 | 2.909952607 | Down |
| 196403 | ENSG00000178498 | K06058 | DTX3 | chr12 | 57604327 | 57609804 | -6.12885547 | 4.964945407 | 6.2E-17 | 1.92E-14 | 0.394285714 | 2.825714286 | Down |
| 84875 | ENSG00000178685 | K15261 | PARP10 | chr8 | 143977152 | 143986471 | -3.095475666 | 2.674084864 | 0.00000239 | 0.0000424 | 0.457087753 | 2.683702989 | Down |
| 23512 | ENSG00000178691 | K11463 | SUZ12 | chr17 | 31937010 | 32001045 | -3.262292926 | 2.765916931 | 0.00000122 | 0.0000252 | 0.51420839 | 2.889039242 | Down |
| 57184 | ENSG00000178761 | - | FAM219B | chr15 | 74898763 | 74907228 | -3.262288622 | 2.765916931 | 0.00000123 | 0.0000252 | 0.494949495 | 2.48989899 | Down |
| 93621 | ENSG00000179010 | - | MRFAP1 | chr4 | 6640091 | 6642745 | -4.135973485 | 3.326189908 | 3.74E-12 | 1.13E-10 | 0.622047244 | 2.952755906 | Down |
| 79730 | ENSG00000179299 | - | NSUN7 | chr4 | 40749874 | 40810601 | -3.784414373 | 4.376575437 | 0.0000224 | 0.000343815 | 0.486072423 | 3.029247911 | Down |
| 2262 | ENSG00000179399 | K08111 | GPC5 | chr13 | 91398619 | 92867237 | -3.517026563 | 4.026762593 | 0.0000477 | 0.000698664 | 0.468531469 | 2.872377622 | Down |
| 139065 | ENSG00000179542 | - | SLITRK4 |  |  |  | -3.095469874 | 2.674084864 | 0.0000024 | 0.0000424 | 0.471923536 | 3.077658303 | Down |
| 8575 | ENSG00000180228 | - | PRKRA | chr2 | 178431414 | 178451240 | -3.095464087 | 2.674084864 | 0.00000241 | 0.0000425 | 0.469648562 | 2.817891374 | Down |
| 245937 | ENSG00000180383 | - | DEFB124 |  |  |  | -4.135966086 | 3.326189908 | 3.77E-12 | 1.14E-10 | 0.366197183 | 3.070422535 | Down |
| 168620 | ENSG00000180535 | K08040 | BHLHA15 |  |  |  | -5.131454352 | 4.096541259 | 1.52E-14 | 1.06E-12 | 0.428571429 | 2.708994709 | Down |
| 85453 | ENSG00000180543 | K11288 | TSPYL5 | chr8 | 97273486 | 97277948 | -3.034961432 | 4.206322663 | 0.00064052 | 0.007329546 | 0.458033573 | 2.673860911 | Down |
| 169200 | ENSG00000180694 | - | TMEM64 | chr8 | 90621995 | 90645905 | -3.179039186 | 4.148862116 | 0.000344667 | 0.004281642 | 0.284210526 | 2.718421053 | Down |
| 124961 | ENSG00000180787 | - | ZFP3 | chr17 | 5078459 | 5096374 | -3.034962604 | 4.206322663 | 0.000641446 | 0.007332817 | 0.527888446 | 2.924302789 | Down |
| 79642 | ENSG00000180801 | K12375 | ARSJ |  |  |  | -4.747795787 | 3.785577849 | 1.02E-13 | 4.65E-12 | 0.452420701 | 3.023372287 | Down |
| 55783 | ENSG00000180917 | K14590 | CMTR2 | chr16 | 71282300 | 71290178 | -3.179040973 | 4.148862116 | 0.000345252 | 0.004284257 | 0.454545455 | 3.14025974 | Down |
| 90843 | ENSG00000180964 | - | TCEAL8 |  |  |  | -5.131449317 | 4.096541259 | 1.53E-14 | 1.06E-12 | 0.58974359 | 2.914529915 | Down |
| 5430 | ENSG00000181222 | K03006 | POLR2A | chr17 | 7484379 | 7514618 | -6.128851498 | 4.964945407 | 6.28E-17 | 1.92E-14 | 0.486868687 | 2.865656566 | Down |
| 347736 | ENSG00000181322 | K19869 | NME9 | chr3 | 138261437 | 138331785 | -3.095458304 | 2.674084864 | 0.00000242 | 0.0000426 | 0.548484848 | 2.872727273 | Down |
| 84282 | ENSG00000181481 | K16272 | RNF135 | chr17 | 30968642 | 30999911 | -3.229795411 | 4.563637906 | 0.000898455 | 0.009774872 | 0.467592593 | 2.824074074 | Down |
| 64764 | ENSG00000182158 | K09048 | CREB3L2 |  |  |  | -5.131444286 | 4.096541259 | 1.55E-14 | 1.07E-12 | 0.553846154 | 2.865384615 | Down |
| 286530 | ENSG00000182162 | K08386 | P2RY8 |  |  |  | -3.095451527 | 2.674084864 | 0.00000243 | 0.0000428 | 0.331476323 | 3.175487465 | Down |
| 8905 | ENSG00000182287 | K12394 | AP1S2 | chrX | 15825806 | 15855014 | -3.229795445 | 4.563637906 | 0.000898474 | 0.009774872 | 0.5 | 3.5 | Down |
| 391002 | ENSG00000182330 | - | PRAMEF8 |  |  |  | -3.26228432 | 2.765916931 | 0.00000123 | 0.0000252 | 0.44092827 | 3.014767932 | Down |
| 8513 | ENSG00000182333 | K14452 | LIPF |  |  |  | -6.128850338 | 4.964945407 | 6.3E-17 | 1.92E-14 | 0.455882353 | 3.18872549 | Down |
| 859 | ENSG00000182533 | K12959 | CAV3 | chr3 | 8733800 | 8746765 | -4.135958504 | 3.326189908 | 3.81E-12 | 1.15E-10 | 0.42384106 | 3.231788079 | Down |
| 283870 | ENSG00000182685 | - | BRICD5 | chr16 | 2209253 | 2213037 | -4.578849247 | 4.784107766 | 0.000000468 | 0.0000111 | 0.388461538 | 2.838461538 | Down |
| 3214 | ENSG00000182742 | K09304 | HOXB4 |  |  |  | -4.135950929 | 3.326189908 | 3.85E-12 | 1.16E-10 | 0.370517928 | 2.860557769 | Down |
| 55794 | ENSG00000182810 | K20096 | DDX28 | chr16 | 68021274 | 68023867 | -4.03498673 | 4.850010943 | 0.0000086 | 0.000139915 | 0.387037037 | 2.901851852 | Down |
| 345557 | ENSG00000182836 | - | PLCXD3 | chr5 | 41306946 | 41510628 | -3.217507081 | 5.113099631 | 0.000849883 | 0.009488566 | 0.46728972 | 3.140186916 | Down |
| 6546 | ENSG00000183023 | K05849 | SLC8A1 | chr2 | 40112146 | 40512452 | -3.51301614 | 5.065560498 | 0.000603955 | 0.006945782 | 0.499486125 | 3.029804728 | Down |
| 729540 | ENSG00000183054 | - | RGPD6 | chr2 | 110513802 | 110610903 | -3.784415771 | 4.376575437 | 0.0000224 | 0.000343815 | 0.549008499 | 2.975070822 | Down |
| 11251 | ENSG00000183134 | K06715 | PTGDR2 |  |  |  | -4.135943361 | 3.326189908 | 3.89E-12 | 1.17E-10 | 0.311392405 | 2.929113924 | Down |
| 83698 | ENSG00000183166 | - | CALN1 | chr7 | 71779491 | 72504271 | -2.962014122 | 3.698629645 | 0.0005082 | 0.00591586 | 0.544061303 | 2.896551724 | Down |
| 493753 | ENSG00000183513 | K18178 | COA5 |  |  |  | -4.688174362 | 3.739046202 | 7.25E-14 | 3.83E-12 | 0.445945946 | 2.905405405 | Down |
| 23194 | ENSG00000183580 | K10273 | FBXL7 |  |  |  | -3.095444755 | 2.674084864 | 0.00000244 | 0.0000429 | 0.460285132 | 2.804480652 | Down |
| 200504 | ENSG00000183607 | - | GKN2 |  |  |  | -4.747790812 | 3.785577849 | 1.03E-13 | 4.68E-12 | 0.423913043 | 3.043478261 | Down |
| 80258 | ENSG00000183690 | - | EFHC2 | chrX | 44146254 | 44343677 | -3.78441723 | 4.376575437 | 0.0000225 | 0.000343835 | 0.526034713 | 3.280373832 | Down |
| 9241 | ENSG00000183691 | K04658 | NOG | chr17 | 56593699 | 56595590 | -4.135935799 | 3.326189908 | 3.93E-12 | 1.18E-10 | 0.375 | 2.909482759 | Down |
| 390928 | ENSG00000183760 | - | ACP7 |  |  |  | -4.74778584 | 3.785577849 | 1.04E-13 | 4.7E-12 | 0.408675799 | 3.200913242 | Down |
| 10317 | ENSG00000183778 | K03877 | B3GALT5 | chr21 | 39612940 | 39662889 | -5.13143859 | 4.096541259 | 1.57E-14 | 1.08E-12 | 0.436305732 | 3.334394904 | Down |
| 90187 | ENSG00000183798 | - | EMILIN3 |  |  |  | -4.135928245 | 3.326189908 | 3.97E-12 | 1.19E-10 | 0.447780679 | 2.650130548 | Down |
| 114781 | ENSG00000183826 | K10481 | BTBD9 | chr6 | 38168451 | 38640426 | -3.262280021 | 2.765916931 | 0.00000124 | 0.0000253 | 0.517973856 | 2.977124183 | Down |
| 219527 | ENSG00000183908 | - | LRRC55 | chr11 | 57181747 | 57191714 | -5.131434046 | 4.096541259 | 1.58E-14 | 1.08E-12 | 0.401759531 | 2.882697947 | Down |
| 7113 | ENSG00000184012 | K09633 | TMPRSS2 | chr21 | 41464551 | 41508158 | -3.338475074 | 4.089072688 | 0.0001367 | 0.001828784 | 0.442344045 | 2.790170132 | Down |
| 2520 | ENSG00000184502 | K13768 | GAST |  |  |  | -4.238338537 | 3.40286445 | 5.85E-09 | 0.000000156 | 0.485148515 | 2.97029703 | Down |
| 352999 | ENSG00000184530 | - | C6orf58 |  |  |  | -5.131429504 | 4.096541259 | 1.6E-14 | 1.08E-12 | 0.512121212 | 3.184848485 | Down |
| 4753 | ENSG00000184613 | - | NELL2 | chr12 | 44508275 | 44921848 | -5.131424965 | 4.096541259 | 1.61E-14 | 1.09E-12 | 0.5 | 2.740184758 | Down |
| 9823 | ENSG00000184867 | - | ARMCX2 | chrX | 101655281 | 101659891 | -3.262275725 | 2.765916931 | 0.00000124 | 0.0000253 | 0.42721519 | 2.53164557 | Down |
| 222546 | ENSG00000185002 | K19521 | RFX6 | chr6 | 116877213 | 116932163 | -5.131420429 | 4.096541259 | 1.63E-14 | 1.09E-12 | 0.509698276 | 2.865301724 | Down |
| 26985 | ENSG00000185009 | K12398 | AP3M1 | chr10 | 74120257 | 74151085 | -3.229796261 | 4.563637906 | 0.000898937 | 0.009774872 | 0.468899522 | 3.098086124 | Down |
| 10519 | ENSG00000185043 | K17259 | CIB1 | chr15 | 90230245 | 90265759 | -4.135920697 | 3.326189908 | 4.01E-12 | 1.19E-10 | 0.601731602 | 2.939393939 | Down |
| 338440 | ENSG00000185101 | K19503 | ANO9 | chr11 | 417930 | 442011 | -3.095437989 | 2.674084864 | 0.00000245 | 0.000043 | 0.413043478 | 3.257033248 | Down |
| 387263 | ENSG00000185127 | - | C6orf120 | chr6 | 169702112 | 169706358 | -4.688168347 | 3.739046202 | 7.33E-14 | 3.85E-12 | 0.481675393 | 2.863874346 | Down |
| 114659 | ENSG00000185158 | - | LRRC37B | chr17 | 32007872 | 32053500 | -5.683906854 | 4.568749497 | 1.87E-15 | 3.58E-13 | 0.546201232 | 2.917864476 | Down |
| 283991 | ENSG00000185262 | - | UBALD2 | chr17 | 76265205 | 76271298 | -3.517029228 | 4.026762593 | 0.0000478 | 0.000698838 | 0.402439024 | 2.762195122 | Down |
| 54622 | ENSG00000185305 | K17201 | ARL15 |  |  |  | -3.239494505 | 4.184151988 | 0.00028309 | 0.003570862 | 0.495098039 | 2.970588235 | Down |
| 5087 | ENSG00000185630 | K09355 | PBX1 | chr1 | 164559360 | 164886047 | -3.262271432 | 2.765916931 | 0.00000125 | 0.0000254 | 0.527906977 | 2.620930233 | Down |
| 4603 | ENSG00000185697 | K09421 | MYBL1 |  |  |  | -4.747780872 | 3.785577849 | 1.05E-13 | 4.72E-12 | 0.569148936 | 2.98537234 | Down |
| 496 | ENSG00000186009 | K01543 | ATP4B |  |  |  | -6.128846319 | 4.964945407 | 6.37E-17 | 1.92E-14 | 0.467353952 | 3.192439863 | Down |
| 57711 | ENSG00000186020 | K09228 | ZNF529 | chr19 | 36534491 | 36605411 | -3.22979651 | 4.563637906 | 0.000899079 | 0.009774872 | 0.520426288 | 3.13321492 | Down |
| 84529 | ENSG00000186073 | - | C15orf41 | chr15 | 36579603 | 36810260 | -3.095431229 | 2.674084864 | 0.00000246 | 0.0000431 | 0.466898955 | 3.153310105 | Down |
| 130355 | ENSG00000186132 | - | C2orf76 |  |  |  | -4.747775907 | 3.785577849 | 1.06E-13 | 4.75E-12 | 0.515873016 | 3.26984127 | Down |
| 55693 | ENSG00000186280 | K06709 | KDM4D | chr11 | 94973681 | 94999514 | -3.262267141 | 2.765916931 | 0.00000125 | 0.0000254 | 0.430210325 | 2.961759082 | Down |
| 150763 | ENSG00000186281 | K00629 | GPAT2 |  |  |  | -3.095424475 | 2.674084864 | 0.00000247 | 0.0000432 | 0.381132075 | 2.973584906 | Down |
| 260293 | ENSG00000186377 | K07428 | CYP4X1 | chr1 | 46961364 | 47055432 | -4.135914514 | 3.326189908 | 4.04E-12 | 1.2E-10 | 0.438113949 | 3.277013752 | Down |
| 257106 | ENSG00000186517 | K20645 | ARHGAP30 | chr1 | 161046942 | 161069971 | -4.135908336 | 3.326189908 | 4.08E-12 | 1.21E-10 | 0.554950045 | 2.611262489 | Down |
| 53373 | ENSG00000186815 | K16896 | TPCN1 | chr12 | 113221429 | 113298589 | -3.229797072 | 4.563637906 | 0.000899397 | 0.009774872 | 0.442567568 | 3.248873874 | Down |
| 256949 | ENSG00000186994 | - | KANK3 |  |  |  | -5.131415897 | 4.096541259 | 1.64E-14 | 1.09E-12 | 0.48452381 | 2.470238095 | Down |
| 5333 | ENSG00000187091 | K05857 | PLCD1 | chr3 | 38007496 | 38029663 | -3.691614044 | 5.387008694 | 0.000240275 | 0.003120225 | 0.498069498 | 2.992277992 | Down |
| 4286 | ENSG00000187098 | K09455 | MITF | chr3 | 69739435 | 69968337 | -3.095418969 | 2.674084864 | 0.00000248 | 0.0000433 | 0.55513308 | 2.790874525 | Down |
| 222194 | ENSG00000187257 | - | RSBN1L | chr7 | 77696426 | 77779803 | -5.131411366 | 4.096541259 | 1.66E-14 | 1.1E-12 | 0.507092199 | 2.920803783 | Down |
| 387129 | ENSG00000187258 | K08376 | NPSR1 |  |  |  | -5.131405599 | 4.096541259 | 1.68E-14 | 1.11E-12 | 0.438461538 | 3.246153846 | Down |
| 9742 | ENSG00000187535 | K19672 | IFT140 |  |  |  | -5.131400053 | 4.096541259 | 1.7E-14 | 1.11E-12 | 0.501367989 | 2.985636115 | Down |
| 646643 | ENSG00000187550 | K08858 | SBK2 | chr19 | 55529336 | 55537175 | -4.135902162 | 3.326189908 | 4.11E-12 | 1.21E-10 | 0.413793103 | 2.893678161 | Down |
| 64170 | ENSG00000187796 | K12794 | CARD9 |  |  |  | -4.135895993 | 3.326189908 | 4.15E-12 | 1.22E-10 | 0.585820896 | 2.994402985 | Down |
| 401944 | ENSG00000187942 | - | LDLRAD2 | chr1 | 21812209 | 21825221 | -3.577576618 | 4.065055456 | 0.0000386 | 0.000575632 | 0.397058824 | 2.595588235 | Down |
| 339451 | ENSG00000187961 | K10454 | KLHL17 | chr1 | 960103 | 965719 | -5.131394512 | 4.096541259 | 1.72E-14 | 1.12E-12 | 0.433021807 | 2.749221184 | Down |
| 285386 | ENSG00000188001 | - | TPRG1 | chr3 | 189000774 | 189325304 | -3.095413133 | 2.674084864 | 0.00000249 | 0.0000434 | 0.505454545 | 3.065454545 | Down |
| 400935 | ENSG00000188263 | - | IL17REL |  |  |  | -3.262262904 | 2.765916931 | 0.00000125 | 0.0000255 | 0.413690476 | 2.883928571 | Down |
| 342897 | ENSG00000188505 | - | NCCRP1 |  |  |  | -4.135885683 | 3.326189908 | 4.2E-12 | 1.24E-10 | 0.436363636 | 2.989090909 | Down |
| 338599 | ENSG00000188716 | K14165 | DUPD1 | chr10 | 75026427 | 75073827 | -4.747770946 | 3.785577849 | 1.07E-13 | 4.77E-12 | 0.540909091 | 3.063636364 | Down |
| 85302 | ENSG00000188878 | K16471 | FBF1 | chr17 | 75909574 | 75938232 | -4.135877887 | 3.326189908 | 4.25E-12 | 1.25E-10 | 0.551393728 | 2.718641115 | Down |
| 120892 | ENSG00000188906 | K08844 | LRRK2 | chr12 | 40224895 | 40369285 | -3.262258669 | 2.765916931 | 0.00000126 | 0.0000255 | 0.475662841 | 3.043529877 | Down |
| 79979 | ENSG00000188917 | K15331 | TRMT2B | chrX | 100973358 | 101052118 | -3.338476535 | 4.089072688 | 0.000136848 | 0.001828784 | 0.410714286 | 3.051587302 | Down |
| 401546 | ENSG00000188959 | - | C9orf152 | chr9 | 110199561 | 110208133 | -5.131388976 | 4.096541259 | 1.74E-14 | 1.12E-12 | 0.439330544 | 2.782426778 | Down |
| 219348 | ENSG00000189129 | - | PLAC9 | chr10 | 80132502 | 80145028 | -3.423035984 | 5.476728747 | 0.000295295 | 0.003720716 | 0.391752577 | 2.670103093 | Down |
| 23254 | ENSG00000189337 | - | KAZN | chr1 | 13893387 | 15118048 | -2.962016334 | 3.698629645 | 0.000508644 | 0.00591586 | 0.529032258 | 2.778064516 | Down |
| 11122 | ENSG00000196090 | K13297 | PTPRT | chr20 | 42072752 | 43189917 | -3.517033002 | 4.026762593 | 0.0000479 | 0.000699457 | 0.487354751 | 2.989747095 | Down |
| 23424 | ENSG00000196116 | K18405 | TDRD7 | chr9 | 97412020 | 97496125 | -4.135870099 | 3.326189908 | 4.29E-12 | 1.26E-10 | 0.482695811 | 3.011839709 | Down |
| 124602 | ENSG00000196169 | K10401 | KIF19 | chr17 | 74326212 | 74355820 | -4.135862317 | 3.326189908 | 4.34E-12 | 1.27E-10 | 0.519038076 | 2.77755511 | Down |
| 8.39E+04 | ENSG00000196182 | K16312 | STK40 | chr1 | 3.63E+07 | 3.64E+07 | -3.10E+00 | 2.67E+00 | 2.50E-06 | 4.35E-05 | 4.82E-01 | 2.93E+00 | Down |

**Table S7: Pathways enriched by genes encoding up regulated and down regulated proteins.**

| **Type** | **Gene Set Name** | **k/K** | **p-value** | **FDR q-value** | **Genes in Gene Set** | **Genes in Overlap** |
| --- | --- | --- | --- | --- | --- | --- |
| Up regulated proteins | Chemokine signaling pathway | 0.0368 | 0.0000524 | 0.00495 | 190 | 7 |
|  | Cardiac muscle contraction | 0.0625 | 0.0000532 | 0.00495 | 80 | 5 |
|  | Prostate cancer | 0.0562 | 0.0000886 | 0.0055 | 89 | 5 |
|  | Arginine and proline metabolism | 0.0741 | 0.000159 | 0.00739 | 54 | 4 |
|  | Regulation of actin cytoskeleton | 0.0278 | 0.000809 | 0.025 | 216 | 6 |
|  | Hypertrophic cardiomyopathy (HCM) | 0.0471 | 0.000901 | 0.025 | 85 | 4 |
|  | Progesterone-mediated oocyte maturation | 0.0465 | 0.000941 | 0.025 | 86 | 4 |
|  | Dilated cardiomyopathy | 0.0435 | 0.00121 | 0.0264 | 92 | 4 |
|  | Pathways in cancer | 0.0213 | 0.00141 | 0.0264 | 328 | 7 |
|  | Alzheimer's disease | 0.0296 | 0.00167 | 0.0264 | 169 | 5 |
| Down regulated proteins | MAPK signaling pathway | 0.0712 | 0.000000122 | 0.0000226 | 267 | 19 |
|  | Calcium signaling pathway | 0.0843 | 0.0000003 | 0.0000279 | 178 | 15 |
|  | Phosphatidylinositol signaling system | 0.1316 | 0.000000485 | 0.0000301 | 76 | 10 |
|  | Pathways in cancer | 0.061 | 0.000000677 | 0.0000315 | 328 | 20 |
|  | Epithelial cell signaling in Helicobacter pylori infection | 0.1324 | 0.00000173 | 0.0000642 | 68 | 9 |
|  | Axon guidance | 0.0853 | 0.0000101 | 0.000312 | 129 | 11 |
|  | Glycerolipid metabolism | 0.1429 | 0.0000148 | 0.000394 | 49 | 7 |
|  | Inositol phosphate metabolism | 0.1296 | 0.0000285 | 0.000662 | 54 | 7 |
|  | Chemokine signaling pathway | 0.0632 | 0.0000806 | 0.0015 | 190 | 12 |
|  | Hypertrophic cardiomyopathy (HCM) | 0.0941 | 8.09E-05 | 1.50E-03 | 85 | 8 |

**Table S8: Functional dissection of key proteins in stomach cancer.**

| **Pathways** | **[O]** | **[C]** | **Gene name** | **Class** | **Gene annotation** |
| --- | --- | --- | --- | --- | --- |
| Cardiac muscle contraction | 0.712280702 | 2.943859649 | TPM3 | Cytoskeleton | Tropomyosin 3, subunit of cytoskeleton |
| Dilated cardiomyopathy | 0.712280702 | 2.943859649 | TPM3 | Cytoskeleton | Tropomyosin3,subunit of cytoskeleton |
| Hypertrophic cardiomyopathy (HCM) | 0.712280702 | 2.943859649 | TPM3 | Cytoskeleton | Tropomyosin3, subunit of cytoskeleton |
| Pathways in cancer | 0.712280702 | 2.943859649 | TPM3 | Cytoskeleton | Tropomyosin 3, subunit of cytoskeleton |
| Pathways in cancer | 0.620165746 | 3.070441989 | HSP90AB1 | Other oncogene | heat shock protein 90 alpha family class B member 1 |
| Progesterone-mediated oocyte maturation | 0.620165746 | 3.070441989 | HSP90AB1 | Other oncogene | heat shock protein 90 alpha family class B member 1 |
| Prostate cancer | 0.620165746 | 3.070441989 | HSP90AB1 | Other oncogene | heat shock protein 90 alpha family class B member 1 |
| Dilated cardiomyopathy | 0.614173228 | 3.023622047 | EMD | Cytoskeleton associated proteins | Emerin: nuclear lamina-associated protein |
| Hypertrophic cardiomyopathy (HCM) | 0.614173228 | 3.023622047 | EMD | Cytoskeleton associated proteins | Emerin: nuclear lamina-associated protein |
| Progesterone-mediated oocyte maturation | 0.564157706 | 3.035842294 | CCNB3 | Cyclins | cyclin B3 |
| Chemokine signaling pathway | 0.548082967 | 2.857950974 | TIAM1 | Other oncogene | T cell lymphoma invasion and metastasis 1 |
| Regulation of actin cytoskeleton | 0.548082967 | 2.857950974 | TIAM1 | Other oncogene | T cell lymphoma invasion and metastasis 1 |
| Regulation of actin cytoskeleton | 0.53952927 | 3.056125528 | IQGAP1 | Cytoskeleton associated proteins | IQ motif containing GTPase activating protein 1, interacts with components of the cytoskeleton, with cell adhesion molecules, and with several signaling molecules to regulate cell morphology and motility |
| Arginine and proline metabolism | 0.533707865 | 3.108614232 | P4HA1 | Other oncogene | prolyl 4-hydroxylase subunit alpha 1 |
| Arginine and proline metabolism | 0.519626168 | 3.059813084 | P4HA2 | Other oncogene | prolyl 4-hydroxylase subunit alpha 2 |
| Regulation of actin cytoskeleton | 0.514619883 | 2.77582846 | ABI2 | Other oncogene | abl interactor 2 |
| Prostate cancer | 0.506329114 | 2.794936709 | CREB3L4 | Other oncogene | cAMP responsive element binding protein 3-like 4 |
| Chemokine signaling pathway | 0.50331525 | 2.927667269 | PREX1 | Other oncogene | phosphatidylinositol-3,4,5-trisphosphate dependent Rac exchange factor 1 |
| Pathways in cancer | 0.497560976 | 3.085365854 | CCNE1 | Cyclins | Cyclins E1 |
| Prostate cancer | 0.497560976 | 3.085365854 | CCNE1 | Cyclins | Cyclins E1 |
| Chemokine signaling pathway | 0.493383743 | 3.015122873 | FGR | Other oncogene | FGR proto-oncogene, Src family tyrosine kinase |
| Pathways in cancer | 0.492357724 | 2.735934959 | LAMA1 | Cytoskeleton | laminin subunit alpha 1 |
| Alzheimer disease | 0.477572559 | 3.09762533 | MAPK3 | Signals in cell cycle progression | cell division |
| Chemokine signaling pathway | 0.477572559 | 3.09762533 | MAPK3 | Signals in cell cycle progression | cell division |
| Pathways in cancer | 0.477572559 | 3.09762533 | MAPK3 | Signals in cell cycle progression | cell division |
| Progesterone-mediated oocyte maturation | 0.477572559 | 3.09762533 | MAPK3 | Signals in cell cycle progression | cell division |
| Prostate cancer | 0.477572559 | 3.09762533 | MAPK3 | Signals in cell cycle progression | cell division |
| Regulation of actin cytoskeleton | 0.477572559 | 3.09762533 | MAPK3 | Signals in cell cycle progression | cell division |
| Chemokine signaling pathway | 0.47659176 | 3.200374532 | PIK3CA | Other oncogene | phosphatidylinositol-4,5-bisphosphate 3-kinase catalytic subunit alpha, which uses ATP to phosphorylate PtdIns, PtdIns4P and PtdIns(4,5)P2. This gene has been found to be oncogenic and has been implicated in cervical cancers. |
| Pathways in cancer | 0.47659176 | 3.200374532 | PIK3CA | Other oncogene | phosphatidylinositol-4,5-bisphosphate 3-kinase catalytic subunit alpha, which uses ATP to phosphorylate PtdIns, PtdIns4P and PtdIns(4,5)P2. This gene has been found to be oncogenic and has been implicated in cervical cancers. |
| Progesterone-mediated oocyte maturation | 0.47659176 | 3.200374532 | PIK3CA | Other oncogene | phosphatidylinositol-4,5-bisphosphate 3-kinase catalytic subunit alpha, which uses ATP to phosphorylate PtdIns, PtdIns4P and PtdIns(4,5)P2. This gene has been found to be oncogenic and has been implicated in cervical cancers. |
| Prostate cancer | 0.47659176 | 3.200374532 | PIK3CA | Other oncogene | phosphatidylinositol-4,5-bisphosphate 3-kinase catalytic subunit alpha, which uses ATP to phosphorylate PtdIns, PtdIns4P and PtdIns(4,5)P2. This gene has been found to be oncogenic and has been implicated in cervical cancers. |
| Regulation of actin cytoskeleton | 0.47659176 | 3.200374532 | PIK3CA | Other oncogene | phosphatidylinositol-4,5-bisphosphate 3-kinase catalytic subunit alpha, which uses ATP to phosphorylate PtdIns, PtdIns4P and PtdIns(4,5)P2. This gene has been found to be oncogenic and has been implicated in cervical cancers. |
| Arginine and proline metabolism | 0.475728155 | 3.067961165 | ASS1 | Other oncogene | argininosuccinate synthase 1, catalyzes the penultimate step of the arginine biosynthetic pathway |
| Alzheimer disease | 0.469050894 | 3.079779917 | CACNA1D | muscle contraction | calcium channel, voltage-dependent, L type, alpha 1D subunit |
| Cardiac muscle contraction | 0.469050894 | 3.079779917 | CACNA1D | muscle contraction | calcium channel, voltage-dependent, L type, alpha 1D subunit |
| Dilated cardiomyopathy | 0.469050894 | 3.079779917 | CACNA1D | muscle contraction | calcium channel, voltage-dependent, L type, alpha 1D subunit |
| Hypertrophic cardiomyopathy (HCM) | 0.469050894 | 3.079779917 | CACNA1D | muscle contraction | calcium channel, voltage-dependent, L type, alpha 1D subunit |
| Chemokine signaling pathway | 0.451612903 | 2.600496278 | CX3CL1 | Other oncogene | C-X3-C motif chemokine ligand 1, belongs to the CX3C subgroup of chemokines |
| Alzheimer disease | 0.437086093 | 2.748344371 | UQCRC2 | Other oncogene | ubiquinol-cytochrome c reductase core protein 2 |
| Cardiac muscle contraction | 0.437086093 | 2.748344371 | UQCRC2 | Other oncogene | ubiquinol-cytochrome c reductase core protein 2 |
| Cardiac muscle contraction | 0.429090909 | 3.087272727 | CACNG5 | Other oncogene | calcium voltage-gated channel auxiliary subunit gamma 5 |
| Dilated cardiomyopathy | 0.429090909 | 3.087272727 | CACNG5 | Other oncogene | calcium voltage-gated channel auxiliary subunit gamma 5 |
| Hypertrophic cardiomyopathy (HCM) | 0.429090909 | 3.087272727 | CACNG5 | Other oncogene | calcium voltage-gated channel auxiliary subunit gamma 5 |
| Pathways in cancer | 0.409090909 | 2.752272727 | CTBP1 | Other oncogene | C-terminal binding protein 1, encodes a protein that binds to the C-terminus of adenovirus E1A proteins. |
| Chemokine signaling pathway | 0.398373984 | 3.227642276 | CCR9 | Other oncogene | C-C motif chemokine receptor 9. The protein encoded by this gene is a member of the beta chemokine receptor family. |
| Alzheimer disease | 0.363984674 | 2.54789272 | HSD17B10 | Other oncogene | hydroxysteroid 17-beta dehydrogenase 10 |
| Arginine and proline metabolism | 0.346153846 | 2.534965035 | PYCRL | Other oncogene | pyrroline-5-carboxylate reductase-like |
| Regulation of actin cytoskeleton | 0.322946176 | 3.354107649 | BDKRB1 | Other oncogene | bradykinin receptor, beta 1 |
| Alzheimer disease | 0.194444444 | 3.125 | COX8C | Other oncogene | cytochrome c oxidase subunit 8C |
| Cardiac muscle contraction | 0.194444444 | 3.125 | COX8C | Other oncogene | cytochrome c oxidase subunit 8C |

**Figures**

**
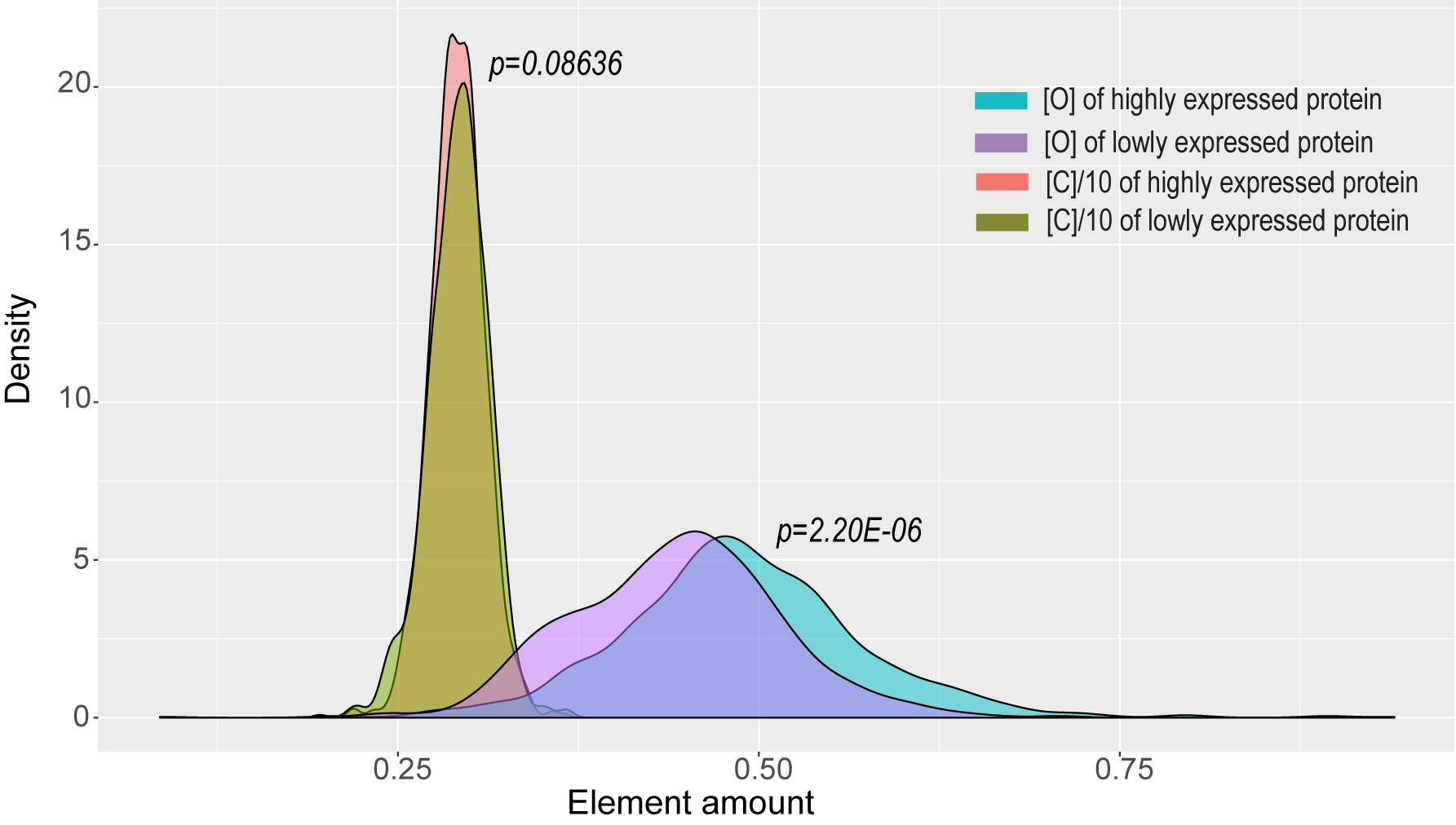
Figure S1: Distribution of oxygen and carbon contents of highly expressed and lowly expressed proteins with a preset threshold score of ≥12/≤0.1 in stomach cancer.**

**
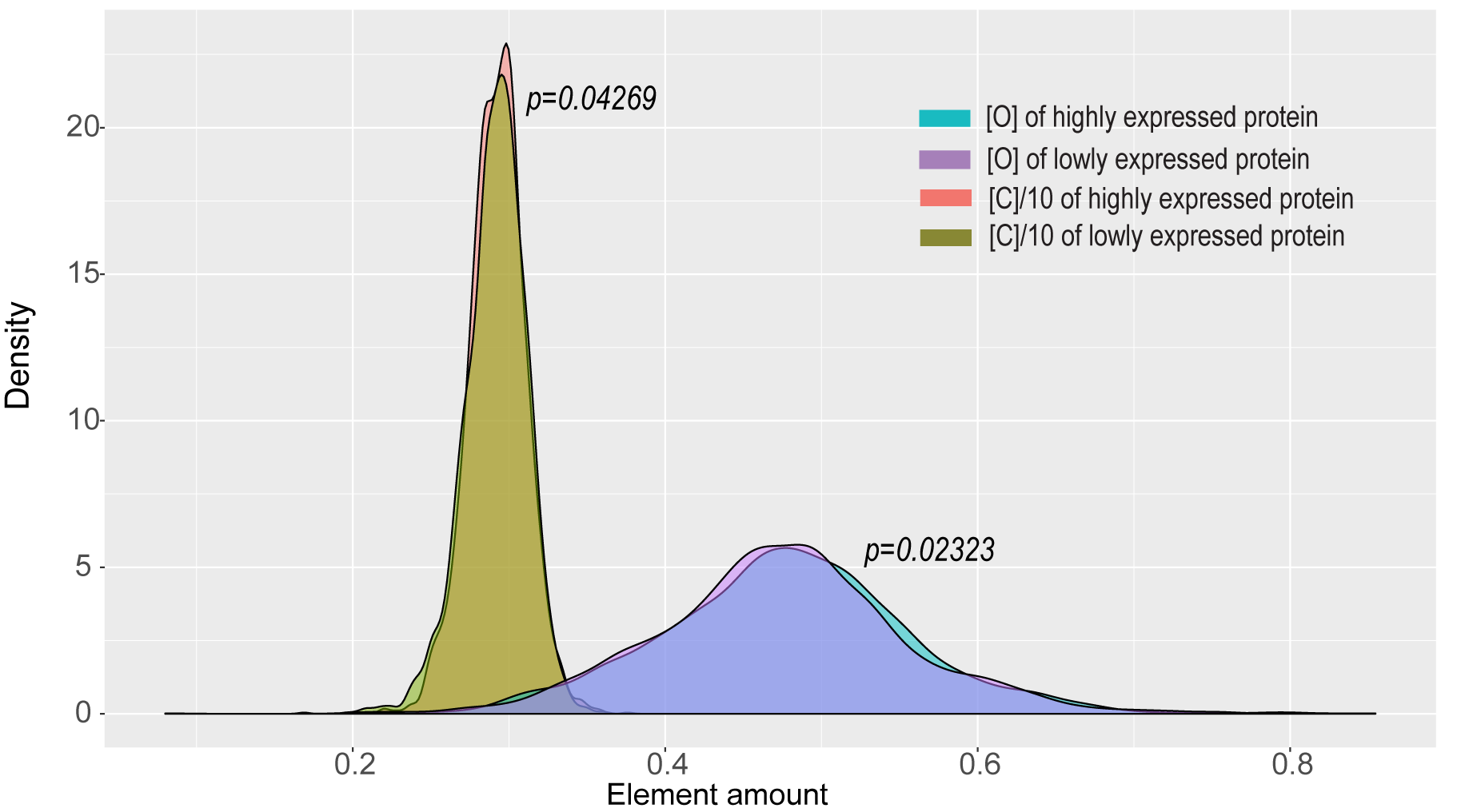
Figure S2: Distribution of oxygen and carbon contents of highly expressed and lowly expressed proteins with a preset threshold score of ≥12/≤0.1 in stomach 1-glandular cells.**

**
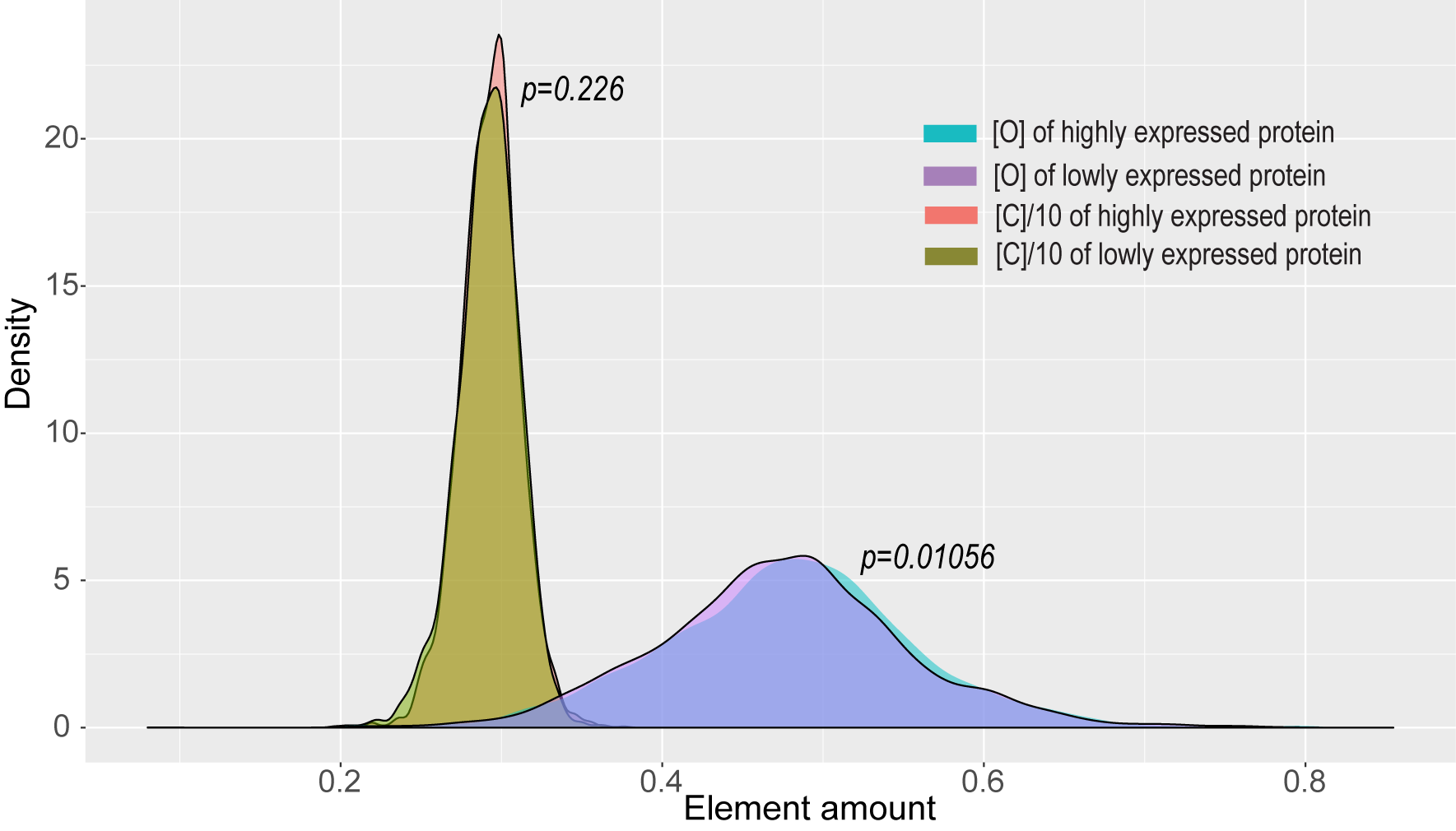
Figure S3: Distribution of oxygen and carbon contents of highly expressed and lowly expressed proteins with a preset threshold score of ≥12/≤0.1 in stomach 2-glandular cells.**
